# Comprehensive analysis of single cell ATAC-seq data with SnapATAC

**DOI:** 10.1038/s41467-021-21583-9

**Published:** 2021-02-26

**Authors:** Rongxin Fang, Sebastian Preissl, Yang Li, Xiaomeng Hou, Jacinta Lucero, Xinxin Wang, Amir Motamedi, Andrew K. Shiau, Xinzhu Zhou, Fangming Xie, Eran A. Mukamel, Kai Zhang, Yanxiao Zhang, M. Margarita Behrens, Joseph R. Ecker, Bing Ren

**Affiliations:** 1 Ludwig Institute for Cancer Research, La Jolla, CA USA; 2 Department of Chemistry and Chemical Biology, Harvard University, Cambridge, MA USA; 3 Center for Epigenomics, Department of Cellular and Molecular Medicine, University of California, San Diego, La Jolla, CA USA; 4 The Salk Institute for Biological Studies, La Jolla, CA USA; 5 Small Molecule Discovery Program, Ludwig Institute for Cancer Research, La Jolla, CA USA; 6 Biomedical Science Graduate Program, University of California San Diego, La Jolla, CA USA; 7 Department of Physics, University of California, San Diego, La Jolla, CA USA; 8 Howard Hughes Medical Institute, The Salk Institute for Biological Studies, La Jolla, CA USA; 9 Department of Cellular and Molecular Medicine, Institute of Genomic Medicine, UCSD Moores Cancer Center, La Jolla, CA USA

**Keywords:** Bioinformatics, Computational biology and bioinformatics, Epigenomics, Sequencing

## Abstract

Identification of the cis-regulatory elements controlling cell-type specific gene expression patterns is essential for understanding the origin of cellular diversity. Conventional assays to map regulatory elements via open chromatin analysis of primary tissues is hindered by sample heterogeneity. Single cell analysis of accessible chromatin (scATAC-seq) can overcome this limitation. However, the high-level noise of each single cell profile and the large volume of data pose unique computational challenges. Here, we introduce SnapATAC, a software package for analyzing scATAC-seq datasets. SnapATAC dissects cellular heterogeneity in an unbiased manner and map the trajectories of cellular states. Using the Nyström method, SnapATAC can process data from up to a million cells. Furthermore, SnapATAC incorporates existing tools into a comprehensive package for analyzing single cell ATAC-seq dataset. As demonstration of its utility, SnapATAC is applied to 55,592 single-nucleus ATAC-seq profiles from the mouse secondary motor cortex. The analysis reveals ~370,000 candidate regulatory elements in 31 distinct cell populations in this brain region and inferred candidate cell-type specific transcriptional regulators.

## Abstract (web summary)

Single cell analysis of transposase-accessible chromatin is deepening our understanding on the origins of cellular diversity, yet methods are limited by data sparsity. Here, the authors introduce SnapATAC, a pipeline to resolve cellular heterogeneity and reveal candidate regulatory elements across different cell populations.

## Introduction

A multicellular organism comprises diverse cell types, each highly specialized to carry out unique functions. Each cell lineage is established during development as a result of tightly regulated spatiotemporal gene expression programs^1^, which are driven in part by sequence-specific transcription factors that interact with *cis*-regulatory sequences in a cell-type specific manner^2^. Thus, identifying the *cis*-elements in the genome and their cellular specificity during development  is an essential step towards understanding the developmental programs encoded in the linear genome sequence.

Since the *cis*-regulatory elements are often marked by hypersensitivity to nucleases or transposases when they are active or poised to act, approaches to detect chromatin accessibility, such as ATAC-seq (Assay for Transposase-Accessible Chromatin using sequencing)^3^ and DNase-seq (DNase I hypersensitive sites sequencing)^4^ have been widely used to map candidate *cis*-regulatory sequences. However, conventional assays that use bulk tissue samples as input cannot resolve cell-type-specific usage of *cis*-elements and lacks the resolution to study their temporal dynamics. To overcome these limitations, a number of methods have been developed for measuring chromatin accessibility in single cells. One approach involves combinatorial indexing to simultaneously analyze tens of thousands of cells^5^. This strategy has been successfully applied to embryonic tissues in D. melanogaster^6^, developing mouse forebrains^7^ and adult mouse tissues^8^. A related method, scTHS-seq (single-cell transposome hypersensitive site sequencing), has also been used to study chromatin landscapes at single-cell resolution in the adult human brains^9^. A third approach relies on isolation of individual cells using microfluidic devices (Fluidigm, C1)^10^ or within individually indexable wells of a nano-well array (Takara Bio, ICELL8)^11^. More recently, single-cell ATAC-seq analysis has been demonstrated on droplet-based platforms^12,13^, enabling profiling of chromatin accessibility from hundreds of thousands cells in a single experiment^13^. Hereafter, these methods are referred to collectively as single-cell ATAC-seq (scATAC-seq).

The growing volume of scATAC-seq datasets coupled with the sparsity of signals in each individual profile due to low detection efficiency (5–15% of peaks detected per cell)^7^ present a unique computational challenge. To address this challenge, a number of unsupervised algorithms have been developed^14^. One approach, chromVAR^15^, groups similar cells together by dissecting the variability of transcription factor (TF) motif occurrence in the open chromatin regions in each cell. Another approach employs the natural language processing techniques such as Latent Semantic Analysis (LSA)^8^ and Latent Dirichlet Allocation (LDA)^16^ to group cells together based on the similarity of chromatin accessibility. A third approach analyzes the variability of chromatin accessibility in cells based on the k-mer composition of the sequencing reads from each cell^13,17^. A fourth approach, Cicero^18^, infers cell-to-cell similarities based on the gene activity scores predicted from their putative regulatory elements in each cell.

Because the current methods often require performing linear dimensionality reduction such as singular value decomposition (SVD) on a cell matrix of hundreds of thousands of dimensions, scaling the analysis to millions of cells remains very challenging or nearly impossible. In addition, the unsupervised identification of cell types or states in complex tissues using scATAC-seq dataset does not have the same degree of sensitivity as that from scRNA-seq^19^. One possibility is that the current methods rely on the use of predefined accessibility peaks based on the aggregate signals. There are several limitations to this choice. First, the cell-type identification could be biased toward the most abundant cell types in the tissues, and consequently lack the ability to reveal regulatory elements in the rare cell populations that could be underrepresented in the aggregate dataset. Second, a sufficient number of single-cell profiles would be required to create robust aggregate signal for creating the peak reference.

To overcome these limitations, we introduce a software package, Single Nucleus Analysis Pipeline for ATAC-seq—SnapATAC (https://github.com/r3fang/SnapATAC)—that does not require population-level peak annotation prior to clustering. Instead, it resolves cellular heterogeneity by directly comparing the similarity in genome-wide accessibility profiles between cells. We also adopt a technique, ensemble Nyström method^20,21^, that improves the computational efficiency and enables the analysis of scATAC-seq from up to a million cells on typical hardware. SnapATAC also incorporates many existing tools, such as integration of scATAC-seq and scRNA-seq dataset^19^, prediction of enhancer–promoter interaction, discovery of key transcription factors^22^, identification of differentially accessible elements^23^, construction of trajectories during cellular differentiation, correction of batch effect^24^ and classification of new dataset based on existing cell atlas^19^, into one single package to maximize its utility and functionalities. Thus, SnapATAC represents a comprehensive solution for scATAC-seq analysis.

Through extensive benchmarking using both simulated and empirical datasets from diverse tissues and species, we show that SnapATAC outperforms current methods in accuracy, sensitivity, scalability and reproducibility for cell-type identification from complex tissues. Furthermore, we demonstrate the utility of SnapATAC by building a high-resolution single-cell atlas of the mouse secondary motor cortex. This atlas comprises of ~370,000 candidate *cis*-regulatory elements across 31 distinct cell types, including rare neuronal cell types that account for less than 0.1% of the total population analyzed. Through motif enrichment analysis, we further infer potential key transcriptional regulators that control cell-type specific gene expression programs in the mouse brain.

## Results

### Overview of SnapATAC workflow

**Fig. 1:**
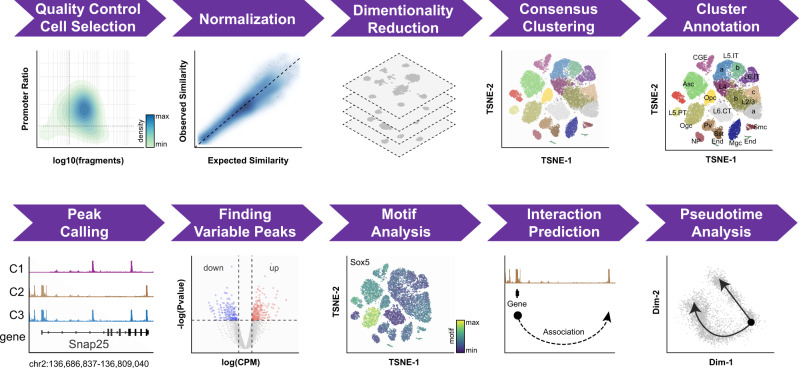
Schematic overview of SnapATAC analysis workflow. See Methods section for details of each step.

A schematic overview of SnapATAC workflow is displayed in Fig. 1. SnapATAC first performs preprocessing of sequencing reads including demultiplexing, reads alignments and filtering, duplicate removal and barcode selection using SnapTools (https://github.com/r3fang/SnapTools) (Methods section). The output of this preprocessing step is a snap (Single-Nucleus Accessibility Profiles) file specially formatted for storing single-cell ATAC-seq datasets (Supplementary Fig. 1a). A snap file contains numerous quality control metrics and users could select high-quality single-cell profiles for subsequent analysis based on metrics such as the number of unique fragments detected from the cell and percentage of promoter-overlapping fragments^25^.

Next, SnapATAC resolves the heterogeneity of cell population by assessing the similarity of chromatin accessibility between cells. To achieve this goal, each single-cell chromatin accessibility profile is represented as a binary vector, the length of which corresponds to the number of uniform-sized bins that segment the genome. Through systematic benchmarking, a bin size of 5 kb is chosen in this study (Supplementary Fig. 2b). A bin with value “1” indicates that one or more reads fall within that bin, and the value “0” indicates otherwise. The set of binary vectors from all the cells are converted into a Jaccard similarity matrix, with the value of each element calculated from the fraction of overlapping bins between every pair of cells. Because the value of Jaccard Index could be influenced by sequencing depth of a cell, a regression-based normalization method is developed to remove such confounding factor (Supplementary Figs. 3–4). Using the normalized similarity matrix, eigenvector decomposition is performed for dimensionality reduction. Finally, in the reduced dimension, SnapATAC uses Harmony^24^ to remove potential batch effect between samples introduced by technical variability (Methods section).

The computational cost of the algorithm scales quadratically with the number of cells. To improve the scalability of SnapATAC, an efficient technique to generate low-rank matrix approximations—the Nyström method^21^—is used to generate the low-rank embedding for large-scale datasets (Methods section). Nyström method contains two major steps: (1) it computes the low-dimension embedding for a subset of selected cells (also known as landmarks); (2) it projects the remaining cells to the embedding structure learned from the landmarks. This achieves significant speedup considering that the number of landmarks could be substantially smaller than the total number of cells. Through benchmarking, we further demonstrate that this approach will not sacrifice the performance once the landmarks are chosen appropriately (Supplementary Fig. 5a–c and Methods section) as reported before^20^.

Nyström method is stochastic and could yield different clustering results in each sampling. To overcome this limitation, a consensus approach is used that combines a mixture of low-dimensional manifolds learned from different sets of sampling (Methods section). Through benchmarking, we demonstrate that the ensemble approach can significantly improve the reproducibility of clustering outcome compared to the standard Nyström method (Supplementary Fig. 5d). In addition, this consensus algorithm naturally fits within the distributed computing environments where their computational costs are roughly the same as that of the standard single sampling method.

As a standalone software package, SnapATAC also provides a number of commonly used functions for scATAC-seq analysis by incorporating many existing useful tools, as described below:

First, to facilitate the annotation of resulting cell clusters, SnapATAC provides three different approaches: (i) SnapATAC annotates the clusters based on the accessibility score at the canonical marker genes (Methods section); (ii) it infers cell-type labels by integrating with corresponding single-cell RNA-seq datasets^19^ (Methods section); (iii) it allows supervised annotation of new single-cell ATAC-seq dataset based on an existing cell atlas (Methods section).

Second, SnapATAC allows identification of the candidate regulatory elements in each cluster by applying peak-calling algorithm^26^ to the aggregate chromatin profiles. Differential analysis is then performed to identify cell-type-specific regulatory elements^23^. Candidate master transcription factors in each cell cluster are discovered through motif enrichment analysis of the differentially accessible regions in each cluster^22^. SnapATAC further conducts Genomic Regions Enrichment of Annotation Tool (GREAT)^27^ analysis to identify the biological pathways active in each cell type.

**Fig. 2:**
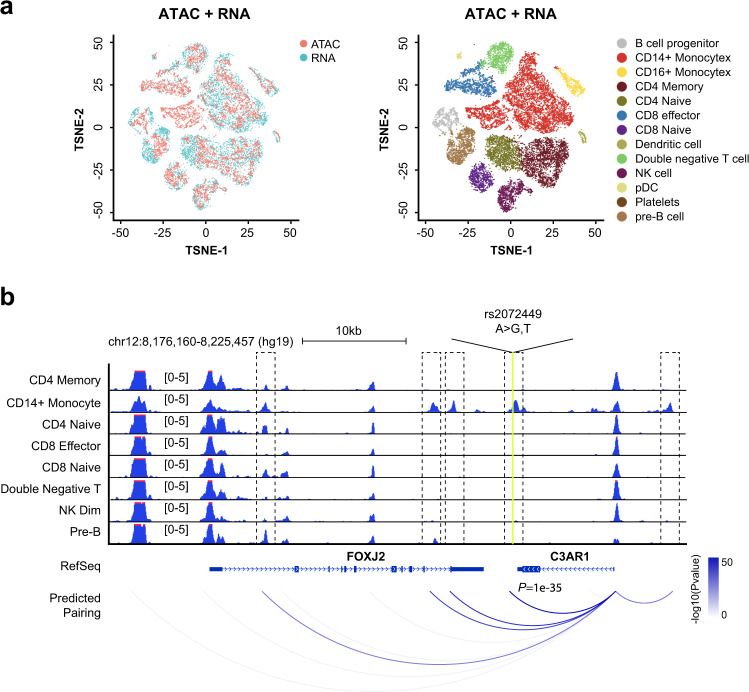
SnapATAC facilitates integration of single-cell ATAC-seq and RNA-seq data to link enhancers to putative target genes. **a** Joint t-SNE visualization of scATAC-seq and scRNA-seq datasets from peripheral blood mononuclear cells (PBMC). Cells are colored by modality (left) and predicted cell types (right). **b** Cell-type-specific chromatin landscapes are shown together with the association score between gene expression of C3AR1 and accessibility at its putative enhancers (logistic regression coefficient *p*-value; Methods section). Dash lines highlight the significant enhancer–promoter pairs. Yellow line represents the SNP (rs2072449) that is previously identified to be associated with C3AR1 expression.

Third, SnapATAC incorporates an approach to link candidate regulatory elements to their putative target genes. In contrast to previous method^18^ that relies on analysis of coaccessibility of putative enhancers and promoters, SnapATAC infers the linkage based on the association between gene expression and chromatin accessibility in single cells where scRNA-seq data is available (Methods section). First, SnapATAC integrates scATAC-seq and scRNA-seq^19^. Second, for each scATAC-seq profile, a corresponding gene expression profile is imputed based on the weighted average of its *k*-nearest neighboring cells (i.e., k = 15) in the scRNA-seq dataset. A “pseudo” multiomics cell is created that contains the information of both chromatin accessibility and gene expression. Finally, logistic regression is used to quantify the association between the gene expression and binarized accessibility state at putative enhancers (Methods section). This approach is used to integrate ~15 K peripheral blood mononuclear cells (PBMC) chromatin profiles and ~10 K PBMC transcriptomic profiles (Fig. 2a). These two datasets are represented in a joint t-SNE embedding space (Fig. 2a) with 98% of the single-cell ATAC-seq cells can be confidently assigned to a cell type defined in the scRNA-seq dataset (Supplementary Fig. 6a). Enhancer-gene pairs are predicted for 3000 genes differentially expressed between cell types in PBMC as determined by scRNA-seq. The validity of the prediction is supported by two lines of evidence. First, the association score exhibits a distance decay from the TSS, consistent with the distance decay of interaction frequency observed in chromatin conformation study^28^ (Supplementary Fig. 6b). Second, the predictions match well with the expression quantitative trait loci (*cis*-eQTLs) derived from interferon-γ and lipopolysaccharide stimulation of monocytes^29^ with reasonable prediction power (AUROC = 0.66, AUPRC = 0.68; Supplementary Fig. 6c–d and Methods section). It is important to note that while statistical association between scATAC-seq and scRNA-seq provides another approach to symmetrically link enhancers to their putative target genes, the predictions would require further experimental validation.

**Fig. 3:**
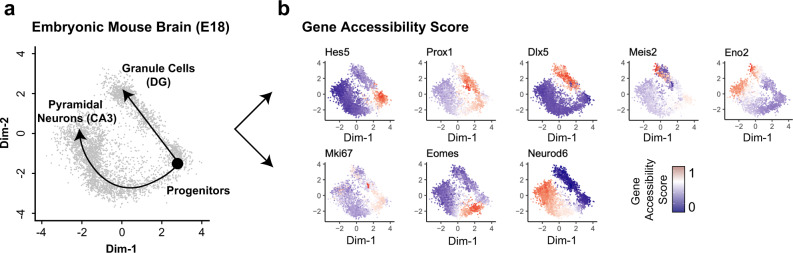
SnapATAC constructs cellular trajectories for the developing mouse brain. **a** Two-dimensional visualization of a dataset that contains 4259 single-cell chromatin profiles from the hippocampus and ventricular zone in embryonic mouse brain (E18) reveals two-branch differentiation trajectories from progenitor cells to Granule Cells (DG) and Pyramidal Neurons (CA3) (left). Data source is listed in Supplementary Table 1. The cellular trajectory is determined using Slingshot. **b** Gene accessibility score (Methods section) of canonical marker genes is projected onto the 2D embedding.

Fourth, SnapATAC has incorporated a function to construct cellular trajectories from single-cell ATAC-seq. As a demonstration of this feature, SnapATAC is used to analyze a dataset that contains 4259 cells from the hippocampus in the fetal mouse brain (E18) (data source listed in Supplementary Table 1). Immature granule cells originating in the dentate gyrus give rise to both mature granule cells (DG) and pyramidal neurons (CA3)^30^. Analysis of 4259 cells reveals a clear branching structure in the first two dimensions (Fig. 3a), the pattern of which is similar to the result previously obtained from single-cell transcriptomic analysis^31^. For instance, the DG-specific transcription factor Prox1 is exclusively accessible in one branch whereas Neurod6 that is known to be specific to CA3 are accessible in the other branch. Markers of progenitors such as Hes5 and Mki67, however, are differentially accessible before the branching point (Fig. 3b). Further using lineage inference tool such as Slingshot^32^, SnapATAC defines the trajectories of cell states for pseudo-time analysis (Fig. 3a). These results demonstrate that SnapATAC can also reveal lineage trajectories with high accuracy.

### Performance evaluation

To compare the accuracy of cell clustering between SnapATAC and published scATAC-seq analysis methods, a simulated dataset of scATAC-seq profiles is generated with varying coverages, from 10,000 (high coverage) to 1000 reads per cell (low coverage) by down sampling from 10 previously published bulk ATAC-seq datasets^22^ (Supplementary Table 2 and Methods section). Based on a recent summary of single-cell ATAC-seq methods^33^, LSA^8^ and *cis*-Topic^16^ outperforms the other methods in separating cell populations of different coverages and noise levels in both synthetic and real datasets. Therefore, we choose to compare SnapATAC with these two methods.

**Fig. 4:**
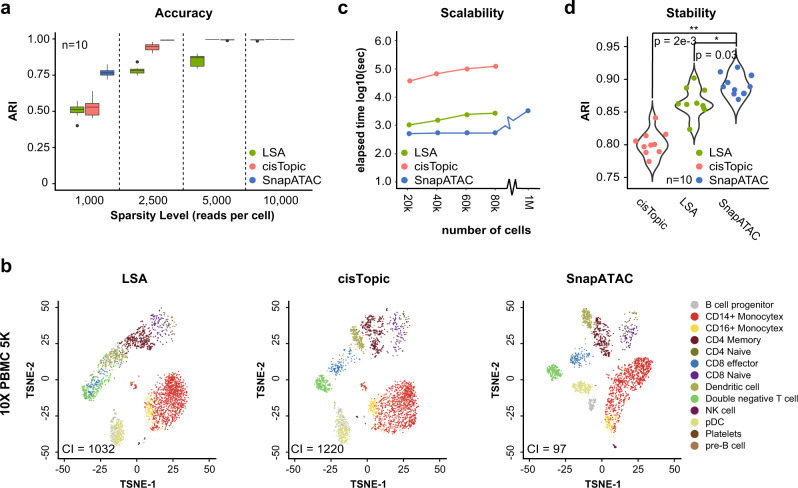
SnapATAC outperforms current methods in accuracy, sensitivity, scalability, and stability of identifying cell types in complex tissues. **a** A set of simulated datasets are generated with varying coverage ranging from 1000 to 10,000 reads per cell cells (Methods section). For each coverage, *n* = 10 random replicates are simulated, and clustering accuracy measurement is based on Adjusted Rank Index (ARI). Data are presented as median values ± 25% percentile. **b** T-SNE representation of PBMC single-cell ATAC-seq profiles analyzed by three methods. The cell-type identification was predicted by 10X PBMC single-cell RNA-seq (CI = connectivity index; Methods section). **c** A mouse dataset^8^ (data source listed in Supplementary Table 1) is sampled to different number of cells ranging from 20k to 1 M. For each sampling, we compared the CPU running time of different methods for dimensionality reduction. **d** A set of perturbations (*n* = 5) are introduced to the mouse atlas dataset by down sampling to 90% of the original sequencing depth. Clustering outcomes are compared between different downsampled datasets (*n* = 10) to estimate the reproducibility. One-tailed *t*-test was performed to estimate the significance level between SnapATAC and each of the other methods (*p < 0.05 and **p < 0.01).

The performance of each method in identifying the original cell types is measured by both Adjusted Rank Index (ARI) and Normalized Mutual Index (NMI). The comparison shows that SnapATAC is the most robust and accurate method across all ranges of data sparsity (Wilcoxon signed-rank test, *p* < 0.01; Fig. 4a; Supplementary Fig. 7). Next, a set of 1423 human cells corresponding to 10 distinct cell types generated using C1 Fluidigm platform, where the ground truth is known^15^, is analyzed by SnapATAC and other methods. Again, SnapATAC correctly identifies the cell types with high accuracy (Supplementary Fig. 8).

To compare the sensitivity of SnapATAC on detecting cell types to that of previously published methods, we analyzed two scATAC-seq datasets representing different types of bio-samples. First, to quantify the clustering sensitivity, we applied an existing integration method to predict the cell type of 4792 PBMC cells using corresponding 10× single-cell RNA-seq by following the tutorial (https://satijalab.org/seurat/v3.1/atacseq_integration_vignette.html). To obtain the most confident prediction, we only kept single-cell ATAC-seq profiles whose cell-type prediction score is greater than 0.9. Using the remaining cells, we calculated the connectivity index (CI; Methods section) in the low-dimension manifold for each of the methods (LSA, *cis*-Topic, and SnapATAC). Connectivity index estimates the degree of separation between clusters in an unbiased manner and a lower connectivity index represents a higher degree of separation between clusters. SnapATAC exhibits substantially higher sensitivity in distinguishing different cell types compared to the other two methods (Fig. 4b). The second is a newly produced dataset that contains 9529 single-nucleus open chromatin profiles generated from the mouse secondary motor cortex. Based on the gene accessibility score at canonical marker genes (Supplementary Fig. 9), SnapATAC uncovers 22 distinct cell populations (Supplementary Fig. 10) whereas alternative methods fail to distinguish the rare neuronal subtypes including Sst (Gad2+ and Sst+), Vip (Gad2+ and Vip+), L6b (Sulf1− and Tl4e+), and L6.CT (Sulf1+ and Foxp2+). These results suggest that SnapATAC outperforms existing methods in sensitivity of separating different cell types in both synthetic and real datasets.

To compare the scalability of SnapATAC to that of existing methods, a previous scATAC-seq dataset that contains over 80k cells from 13 different mouse tissues^8^ is used (data source listed in Supplementary Table 1). This dataset is downsampled to different number of cells, ranging from 20,000 to 80,000 cells. For each sampling, SnapATAC and other methods are performed, and the CPU running time of dimensionality reduction is monitored (Methods section). The running time of SnapATAC scales linearly and increases at a significantly lower slope than alternative methods (Fig. 4c). Using the same computing resource, when applied to 100k cells, SnapATAC is much faster than existing methods (Fig. 4c). For instance, when applied to 100k cells, SnapATAC is nearly 10 times faster than LSA and more than 100 times faster than *cis*-Topic. More importantly, because SnapATAC avoids the loading of the full cell matrix in the memory and can naturally fit within the distributed computing environments (Methods section), the running time and memory usage for SnapATAC plateau after 20,000 cells, making it possible for analyzing datasets of even greater volumes. To test this, we simulate 1 million cells of the same coverage with the above dataset (Methods section) and process it with SnapATAC, LSA, and *cis*-Topic. Using the same computing resource, SnapATAC is able to process up to one million cells with regular hardwire configuration (Fig. 4c and Methods section). These results demonstrate that SnapATAC provides a highly scalable approach for analyzing large-scale scATAC-seq dataset.

To evaluate the clustering reproducibility, the above mouse scATAC-seq dataset is downsampled to 90% of the original sequencing depth in five different iterations. Each downsampled dataset is clustered using SnapATAC and other methods. Clustering results are compared between sampled datasets to estimate the stability. SnapATAC has a substantially higher reproducibility of clustering results between different downsampled datasets than other methods (Fig. 4d).

The improved performance of SnapATAC likely results from the fact that it considers all reads from each cell, not just the fraction of reads within the peaks defined in the population. To test this hypothesis, clustering is performed after removing the reads overlapping the predefined peak regions. Although the outcome is worse than the full dataset as expected, it still recapitulates the major cell types obtained from the full dataset (Supplementary Fig. 11). This holds true for all three datasets tested (Supplementary Fig. 11a–c). One possibility is that the off-peak reads may be enriched for the euchromatin (or compartment A) that strongly correlates with active genes^28^ and varies considerably between cell types^34,35^. Consistent with this hypothesis, the density of the nonpeak reads in scATAC-seq library is highly enriched for the euchromatin (compartment A) as defined using genome-wide chromatin conformation capture analysis (i.e., Hi–C) in the same cell type^28^ (Supplementary Fig. 12). These observations suggest that the nonpeak reads discarded by existing methods can actually contribute to distinguish different cell types.

Including the off-peak reads, however, raises a concern regarding whether SnapATAC is sensitive to technical variations (also known as batch effect). To test this, SnapATAC is applied to four datasets generated using different technologies (data source listed in Supplementary Table 1). Each dataset contains at least two biological replicates produced by the same technology. In all cases, the biological replicates are well mixed in the t-SNE embedding space showing no obvious batch effect (Supplementary Fig. 13), suggesting that SnapATAC is robust to the technical variations.

To test whether SnapATAC is robust to technical variation introduced by different technological platforms, it is used to integrate two mouse brain datasets generated using plate and droplet-based scATAC-seq technologies (data source listed in Supplementary Table 1). In the joint t-TSNE embedding space, these two datasets are separated based on the technologies (Supplementary Fig. 14a). To remove the platform-to-platform variations, Harmony^24^, a single-cell batch effect correction tool, is incorporated into the SnapATAC pipeline (Methods section). After correction by Harmony^24^, these two datasets are well mixed in the joint t-SNE embedding (Supplementary Fig. 14b) and clusters are fairly represented by both datasets (Supplementary Fig. 14c).

### A high-resolution *cis*-regulatory atlas of the mouse secondary motor cortex

To demonstrate the utility of SnapATAC in resolving cellular heterogeneity of complex tissues and identify candidate *cis*-regulatory elements in diverse cell type, it is applied to a new single-nucleus ATAC-seq dataset generated from the secondary mouse motor cortex in the adult mouse brain as part of the BRAIN Initiative Cell Census Consortium^36^ (Supplementary Fig. 15a). This dataset includes two biological replicates, each pooled from 15 mice to minimize potential batch effects. The aggregate signals show high reproducibility between biological replicates (Pearson correlation = 0.99; Supplementary Fig. 15b–d), a significant enrichment for transcription start sites (TSS) and less than 1% of mitochondria DNA (3), indicating a high signal-to-noise ratio (Supplementary Fig. 15e). After filtering out the low-quality nuclei (Supplementary Fig. 16a) and removing putative doublets using Scrublet^37^ (Methods section; Supplementary Fig. 16b), a total of 55,592 nuclear profiles with an average of ~5000 unique fragments per nucleus remain and are used for further analysis (Supplementary Data 1).

**Fig. 5:**
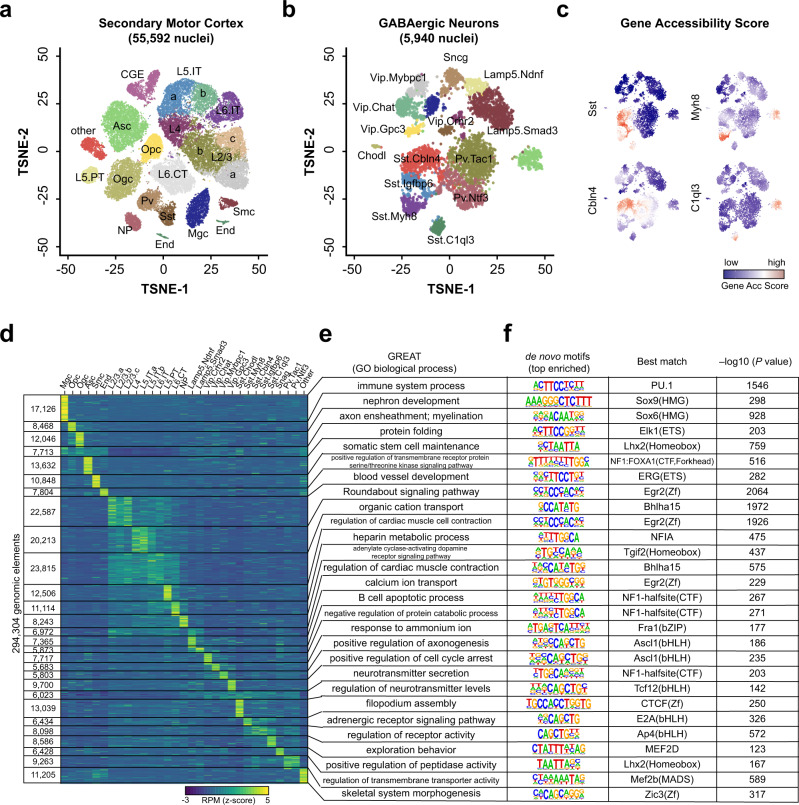
A high-resolution *cis*-regulatory atlas of mouse secondary motor cortex (MOs). **a** T-SNE visualization of 20 major cell types in MOs identified using SnapATAC. **b** Fourteen GABAergic subtypes revealed by iterative clustering of 5940 GABAergic neurons (Sst, Pv, and CGE in **a**). **c** Gene accessibility score of canonical marker genes for GABAergic subtypes is projected onto the t-SNE embedding. **d** *k*-means clustering of 294,304 differentially accessible elements based on chromatin accessibility. **e** Gene ontology analysis of each cell-type predicted by GREAT analysis^27^. **f** Transcription factor motif enriched in each cell group identified using Homer^22^ (Poisson *p*-value).

SnapATAC identifies initially a total of 20 major clusters using the consensus clustering approach (Supplementary Fig. 17). The clustering result is highly reproducible between biological replicates (Pearson correlation = 0.99; Supplementary Fig. 18a) and is resistant to sequencing depth effect (Supplementary Fig. 18b). Based on the gene accessibility score at the canonical marker genes (Supplementary Fig. 19), these clusters are classified into 10 excitatory neuronal subpopulations (Snap25+, Slc17a7+, Gad2−; 52% of total nuclei), three inhibitory neuronal subpopulations (Snap25+, Gad2+; 10% of total nuclei), one oligodendrocyte subpopulation (Mog+; 8% of total nuclei), one oligodendrocyte precursor subpopulation (Pdgfra+; 4% of total nuclei), one microglia subpopulation (C1qb+; 5% of total nuclei), one astrocyte subpopulation (Apoe+; 12% of total nuclei), and additional populations of endothelial, and smooth muscle cells accounting for 6% of total nuclei (Fig. 5a).

In mammalian brain, GABAergic interneurons exhibit spectacular diversity that shapes the spatiotemporal dynamics of neural circuits underlying cognition^38^. To examine whether iterative analysis could help tease out various subtypes of GABAergic neurons, SnapATAC is applied to the 5940 GABAergic nuclei (CGE, Sst, and Vip) identified above, finding 17 distinct subpopulations (Supplementary Fig. 20a) that are highly reproducible between biological replicates (Pearson correlation = 0.99; Supplementary Fig. 20b). Based on the chromatin accessibility at the marker genes (Supplementary Fig. 21), these 17 clusters are classified into five Sst subtypes (Chodl+, Cbln4+, Igfbp6+, Myh8+, and C1ql3+), two Pv subtypes (Tac1+ and Ntf3+), two Lamp5 subtypes (Smad3+ and Ndnf+), four Vip subtypes (Mybpc1+, Chat+, Gpc3+, Crhr2+), Sncg and putative doublets (Fig. 5b). These clusters include a rare type Sst-Chodl (0.1%) previously identified in single-cell RNA^39^ and single-cell ATAC-seq analysis^40^. While the identity and function of these subtypes require further experimental validation, our results demonstrate the exquisite sensitivity of SnapATAC in resolving distinct neuronal subtypes with only subtle differences in the chromatin landscape.

A key utility of single-cell chromatin accessibility analysis is to identify regulatory sequences in the genome. By pooling reads from nuclei in each major cluster (Fig. 5a), cell-type-specific chromatin landscapes can be obtained (Supplementary Fig. 22 and Methods section). Peaks are determined in each cell type, resulting in a total of 373,583 unique candidate *cis*-regulatory elements. Most notably, 56% (212,730/373,583) of these open chromatin regions cannot be detected from bulk ATAC-seq data of the same brain region (Methods section). The validity of these additional open chromatin regions identified from scATAC-seq data are supported by several lines of evidence. First, these open chromatin regions are only accessible in minor cell populations (Supplementary Fig. 23a) that are undetectable in the bulk ATAC-seq signal. Second, these sequences show significantly higher conservation than randomly selected genomic sequences with comparable mappability scores (Supplementary Fig. 23c). Third, these open chromatin regions display an enrichment for transcription factor (TF) binding motifs corresponding to the TFs that play important regulatory roles in the corresponding cell types. For example, the binding motif for Mef2c is highly enriched in novel candidate *cis*-elements identified from Pvalb neuronal subtype (*P*-value = 1e-363; Supplementary Fig. 23d), consistent with previous report that Mef2c is upregulated in embryonic precursors of Pv interneurons^41^. Finally, the new open chromatin regions tend to test positive in transgenic reporter assays. Comparison to the VISTA enhancer database^42^ shows that enhancer activities of 256 of the newly identified open chromatin regions have been previously tested using transgenic reporter assays in e11.5 mouse embryos. Sixty five percent (167/256; 65%) of them drive reproducible reporter expression in at least one embryonic tissue, which was substantially higher than background rates (9.7%) estimated from regions in the VISTA database that lack canonical enhancer mark^43^. Four examples are displayed (Supplementary Fig. 23e).

SnapATAC identifies 294,304 differentially accessible elements between cell types (Methods section; Fig. 5d). GREAT analysis (Fig. 5e) and motif inference (Fig. 5f) identify the master regulators and transcriptional pathways active in each of the cell types. For instance, the binding motif for ETS-factor PU.1 is highly enriched in microglia-specific candidate CREs, motifs for SOX proteins are enriched in Ogc-specific elements, and bHLH motifs are enriched in excitatory neurons-specific CREs (Fig. 5f). Interestingly, motifs for candidate transcriptional regulators, including NUCLEAR FACTOR 1 (NF1), are also enriched in candidate CREs detected in two inhibitory neuron subtypes (Lamp5.Ndnf and Lamp5.Smad3). Motif for CTCF, a multifunctional protein in genome organization and gene regulation^44^, is highly enriched in Sst-Chodl. Finally, motifs for different basic-helix-loop-helix (bHLH) family transcription factors, known determinants of neural differentiation^45^, show enrichment for distinct Sst subtypes. For instance, E2A motif is enriched in candidate CREs found in Sst.Myh8 whereas AP4 motif is specifically enriched in peaks found in Sst.Cbln4, suggesting specific role that different bHLH factors might play in different neuronal subtypes.

### SnapATAC enables reference-based annotation of new scATAC-seq datasets

**Fig. 6:**
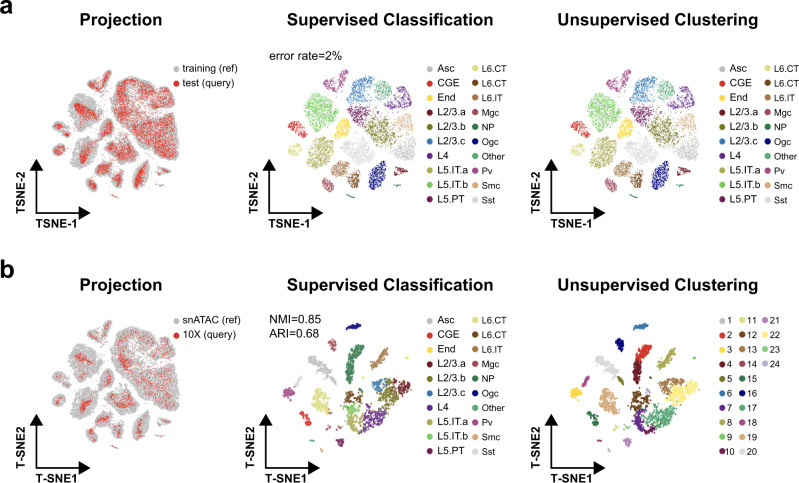
SnapATAC enables supervised annotation of new scATAC-seq dataset using reference cell atlas. **a** MOs snATAC-seq dataset is split into 80% and 20% as training and test dataset. A predictive model learned from the training dataset predicts cell types on the test dataset of high accuracy (left) as compared to the original cell-type labels (right). **b** A predictive model learned from the reference dataset—MOs (snATAC)—accurately predicts the cell types on a query dataset from mouse brain generated using a different technological platform, the 10× scATAC-seq. The t-SNE embedding is inferred from the reference cell atlas (left) or generated by SnapATAC in an unbiased manner from 10× mouse brain dataset (middle and right). Cells are visualized using t-SNE and are colored by the cell types predicted by supervised classification (middle) compared to the cluster labels defined using unsupervised clustering (right).

Unsupervised clustering of scATAC-seq datasets frequently requires manual annotation, which is labor-intensive and limited to prior knowledge. To overcome this limitation, SnapATAC provides a function to project new single-cell ATAC-seq datasets to an existing cell atlas to allow for supervised annotation of cells. First, the Nyström method is used to project the query cells to the low-dimension manifold precomputed from the reference cells (Methods section). In the joint manifold, a neighborhood-based classifier is used to determine the cell type of each query cell based on the label of its *k*-nearest neighboring cells in the reference dataset (Methods section). The accuracy of this method is determined by five-fold cross validation using the mouse motor cortex atlas. On average, 98% (±1%) of the cells can be correctly classified, suggesting a high accuracy of the method (Fig. 6a).

To demonstrate that SnapATAC could be applied to datasets generated from distinct technical platforms, it is used to annotate 4,098 scATAC-seq profiles from mouse brain cells generated using a droplet-based platform (data source listed in Supplementary Table 1). After removing batch effect introduced by different platforms using Harmony^24^, the query cells are well mixed with the reference cells in the joint embedding space (Supplementary Fig. 24). The predicted cluster labels are also consistent with the cell types defined using unbiased clustering analysis (NMI = 0.85, ARI = 0.68; Fig. 6b).

To investigate whether SnapATAC could recognize cell types in the query dataset that are not present in the reference atlas, multiple query datasets are sampled from the above mouse motor cortex dataset and a perturbation is introduced to each sampling by randomly dropping a cell cluster. When this resulting query dataset is analyzed by SnapATAC against the original cell atlas, the majority of the cells that are left out from the original atlas are filtered out due to the low prediction score (Supplementary Fig. 25), again suggesting that our method is not only accurate but also robust to the novel cell types in the query dataset.

## Discussion

In summary, SnapATAC is a comprehensive bioinformatic solution for single-cell ATAC-seq analysis. The open-source software runs on standard hardware, making it accessible to a broad spectrum of researchers. Through extensive benchmarking, we have demonstrated that SnapATAC outperforms existing tools in sensitivity, accuracy, scalability, and robustness of identifying cell types in complex tissues.

SnapATAC differs from previous methods in at least seven aspects. First, SnapATAC incorporates many useful tools and represents the most comprehensive solution for single-cell ATAC-seq data analysis to date. In addition to clustering analysis, SnapATAC provides preprocessing, annotation, trajectory analysis, peak calling^26^, differential analysis^23^, batch effect correction^24^, and motif discovery^22^ all in one package. Second, SnapATAC identifies cell types in an unbiased manner without the need for population-level peak annotation, leading to superior sensitivity for identifying rare cell types in complex tissues. Third, with Nyström method^46^, SnapATAC significantly reduces both CPU and memory usage, enabling analysis of large-scale dataset of a million cells or more. Fourth, SnapATAC not only incorporates existing method to integrate scATAC-seq with scRNA-seq dataset^19^ but also provides a new method to predict promoter–enhancer pairing relations based on the statistical association between gene expression and chromatin accessibility in single cells. Fifth, our method achieves high clustering reproducibility using a consensus clustering approach. Finally, SnapATAC also enables supervised annotation of a new scATAC-seq dataset based on an existing reference cell atlas.

It is important to note that a different strategy has been used to overcome the bias introduced by population-based peak annotation^8^. This approach involves iterative clustering, with the first round defining the crude clusters in complex tissues followed by identifying peaks in these clusters, which are then used in subsequent round(s) of clustering. However, several limitations still exist. First, the strategy of iterative clustering requires multiple rounds of clustering, aggregation, and peak calling, thus hindering its application to large-scale datasets. Second, the crude clusters represent the most dominant cell types in the tissues; therefore, peaks in the rare populations may still be underrepresented. Finally, peak-based methods hinder multi-sample integrative analysis where each sample has its own unique peak reference.

Finally, SnapATAC is applied to a newly generated scATAC-seq dataset including 55,592 high-quality single-nucleus ATAC-seq profiles from the mouse secondary motor cortex, resulting in a single-cell atlas consisting of >370,000 candidate *cis*-regulatory elements across 31 cell types in this mouse brain region. The cellular diversity identified by chromatin accessibility is at a high resolution and is consistent with mouse neurogenesis and taxonomy revealed by single-cell transcriptome data^39,47^. Besides characterizing the constituent cell types, SnapATAC identifies candidate *cis*-regulatory sequences in each of the major cell types and infers the likely transcription factors that regulate cell-type specific gene expression programs. Importantly, a large fraction (56%) of the candidate *cis*-elements identified from the scATAC-seq data are not detected in bulk analysis. While further experiments to thoroughly validate the function of these additional open chromatin regions are needed, the ability for SnapATAC to uncover *cis*-elements from rare cell types of a complex tissue will certainly help expand the catalog of *cis*-regulatory sequences in the genome.

## Methods

### SnapATAC pipeline workflow

#### Fastq file demultiplexing

Using a custom python script, we first perform FASTQ file demulticomplexing by integrating the cell barcode into the read name in the following format: @+barcode+:+original_read_name.

#### Sequencing reads alignment

Demultiplexed sequencing reads are aligned to the corresponding reference genome (i.e., mm10 or hg19) using bwa (0.7.13-r1126) in pair-end mode with default parameter settings. Aligned reads are then sorted based on the read name using samtools (v1.9) to group together reads originating from the same barcodes.

#### Quality control and reads filtering

Pair-end reads are converted into fragments and only those that meet the following criteria are kept: (1) properly paired (according to SMA flag value); (2) uniquely mapped (MAPQ > 30); (3) insert distance within [50–1000 bp]. PCR duplicates (fragments sharing the same genomic coordinates) are removed for each cell separately. Given that Tn5 introduces a 9 bp staggered, reads mapping to the positive and negative strand were shifted by +4/−5 bp, respectively^48^.

#### Barcode filtering

We identify the high-quality cells based on two criteria: (1) total number of unique fragment count [>1000]; (2) fragments in promoter ratio—the percentage of fragments overlapping with annotated promoter regions [0.2–0.8]. The promoter regions used in this study are downloaded from 10× genomics for hg19 and mm10.

#### Snap-file generation

Using the remaining fragments, we next generate a snap-format (Single-Nucleus Accessibility Profiles) file using snaptools (https://github.com/r3fang/SnapTools). A snap file is a hierarchically structured hdf5 file that contains the following sections: header (HD), cell-by-bin matrix (BM), cell-by-peak matrix (PM), cell-by-gene matrix (GM), barcode (BD), and fragment (FM). HD session contains snap-file version, date, alignment and reference genome information. BD session contains all unique barcodes and corresponding meta data. BM session contains cell-by-bin matrices of different bin sizes. PM session contains cell-by-peak count matrix. GM session contains cell-by-gene count matrix. FM session contains all usable fragments for each cell. Fragments are indexed based on barcodes that enables fast retrieval of reads belonging to the same barcodes. Detailed information about snap file can be found here: https://github.com/r3fang/SnapTools/blob/master/docs/snap_format.docx.

#### Creating cell-by-bin count matrix

Using the resulting snap file, we next create cell-by-bin count matrix. The genome is segmented into uniform-sized bins and single-cell ATAC-seq profiles are represented as cell-by-bin matrix with each element indicating number of sequencing fragments overlapping with a given bin in a certain cell.

#### Choosing bin size

To evaluate the effect of bin size to clustering performance, we apply SnapATAC to three datasets namely 5 K PBMC (10×), Mouse Brain (10×), and MOs-M1 (snATAC) (data source listed in Supplementary Table 1). These datasets are generated by both plate and droplet platforms using either cell or nuclei with considerably different depth, allowing us to systematically evaluate the effect of bin size.

For each dataset, we define the “landmark” cell types in a supervised manner. First, we perform *cis*-Topic^16^ for dimensionality reduction and identify cell clusters using graph-based algorithm Louvain^49^ with k = 15. Second, we manually define the major cell types in each dataset by examining the gene accessibility score at the canonical marker genes (see Supplementary Fig. 9 as an example for MOs-M1). Third, clusters sharing the same marker genes are manually merged and those failing to show unique signatures are discarded. In total, we define nine most convincing cell types in PBMC 5 K (10×), 14 types in Mouse Brain 5 K (10×) and 14 types in MOs-M1 (snATAC). Among these cell types, 14 cell populations that account for less than 2% of the total population are considered as rare cell populations (Supplementary Fig. 2a).

We next evaluate the performance of each bin size selection using three metrics: (1) cluster connectivity index (CI), which estimate the degree of connectedness of the landmark cell types; a lower CI represents a better separation. The connectivity index is computed in the following manner. For each cell *i*, the *K* (*K* = 15) nearest neighbors are found and sorted from the closest to furthest. The algorithm checks if those neighbors are assigned to the same cluster with cell *i*. At the beginning, connectivity value equals 0 and increases with value 1/*i* when the *i*th nearest neighbors is not assigned to the same cluster with cell *i*. This procedure is repeated for all cells in the dataset. In general, the higher the connectivity index is, the less separated the defined landmark cell types are. The connectivity index is computed using “connectivity” function implemented in R package clv. (2) coverage bias, which estimates the read depth distribution in the two-dimensional embedding space; (3) sensitivity to identify rare populations. Through systematic benchmarking, we found that bin size in the range from 1 to 10 kb appeared to work well on the three benchmarks, we selected 5 kb as the default bin width for all the analysis in this work (Supplementary Fig. 2 and Methods section).

#### Matrix binarization

We found that the vast majority of the elements in the cell-by-bin count matrix is “0”, indicating either closed chromatin or missing value. Among the non-zero elements, some has abnormally high coverage (>200) perhaps due to the alignment errors or other unknown reasons. These items usually account for less than 0.1% of total non-zero items in the matrix. Thus, we change the top 0.1% elements in the matrix to “0” to eliminate potential alignment errors. We next convert the remaining non-zero elements to “1”.

#### Bin filtering

We next filter out any bins overlapping with the ENCODE blacklist downloaded from http://mitra.stanford.edu/kundaje/akundaje/release/blacklists/ for corresponding reference genome. Second, we remove reads mapped to the X/Y chromosomes to eliminate sex effect. Third, we remove mitochondrial DNA to get rid of potential contamination. We next sort the bins based on the coverage and filter out the top 5% to remove the invariant features. Please note that we do not perform coverage-based bin filtering for a dataset that has low coverage (average fragment number less than 5000) where the ranking of bin may be fluctuated by the noise.

#### Dimensionality reduction

We next apply the following dimensionality reduction procedure to project the high-dimension data to a low-dimension manifold for clustering and visualization. Let ${\mathbf{X}} \in {\mathbf{{\cal{R}}}}^{n \times m}$ be a dataset with *n* cells and *m* bins and ${\mathbf{X}} = \{ 0,1\}$. The first step is to compute a similarity matrix between the *m* high-dimensional data points to construct the *n*-by-*n* pairwise similarity matrix using a kernel function kn that is an appropriate similarity metric. A popular choice is gaussian kernel:

1 $${\mathrm{kn}}\left( {{\mathbf{x}}_{\mathbf{i}},{\mathbf{x}}_{\mathbf{j}}} \right) = {\mathrm{exp}}( { - \frac{{{\mathrm{d}}({\mathbf{x}}_{\mathbf{i}},{\mathbf{x}}_{\mathbf{j}})^2}}{{\it{\epsilon }}}})$$

where ${\mathrm{d}}({\mathbf{x}}_{\mathbf{i}},{\mathbf{x}}_{\mathbf{j}})$ is the Euclidean distance between observations *i* and *j*.

Due the binarization nature of single-cell ATAC-seq dataset, in this case, we replace the Gaussian kernel with Jaccard coefficient, which estimates the similarity between cells simply based on ratio of overlap over the total union:

2 $${\mathrm{jaccard}}( {{\mathbf{x}}_{\mathbf{i}},{\mathbf{x}}_{\mathbf{j}}}) = \frac{{\left| {{\mathbf{x}}_{\mathbf{i}} \,{\cap}\, {\mathbf{x}}_{\mathbf{j}}} \right|}}{{\left| {{\mathbf{x}}_{\mathbf{i}}{\cup} {\mathbf{x}}_{\mathbf{j}}} \right|}}$$

The Jaccard coefficient, which is symmetric and positivity preserving meets the requirement of being a kernel function.

Using jaccard as a kernel function, we next form a symmetric kernel matrix ${\mathbf{J}} \in {\mathbf{{\cal{R}}}}^{n \times n}$ where each entry is obtained as $J_{i,j} = {\mathrm{jaccard}}({\mathbf{x}}_{\mathbf{i}},{\mathbf{x}}_{\mathbf{j}})$.

Theoretically, the similarity *J*_*i,j*_ would reflect the true similarity between cell **x**_***i***_ and **x**_***j***_, but unfortunately, due to the high-dropout rate, this is not the case. If there is a high sequencing depth for cell **x**_**i**_ or **x**_**j**_, then *J*_*i,j*_ tend to have higher values, regardless whether cell **x**_**i**_ and **x**_**j**_ is actually similar or not.

This can be proved theoretically. Given 2 cells **x**_**i**_ and **x**_**j**_ and corresponding coverage (number of “1”s) $C_i = \mathop {\sum }\nolimits_k^m x_{ik}$ and $C_j = \mathop {\sum }\nolimits_k^m x_{jk}$, let $P_i = C_i/m$ and ${P_j = C_j/m}$ be the probability of observing a signal in cell **x**_**i**_ and **x**_**j**_ where *m* is the length of the vector. Assuming **x**_**i**_ and **x**_**j**_ are two “random” cells without any biological relevance, in another word, the “1”s in **x**_**i**_ and **x**_**j**_ are randomly distributed, then the ratio of expectation between cell **x**_**i**_ and **x**_**j**_ can be calculated as:

3 $$E_{ij} = \frac{{P_iP_j}}{{P_i + P_j - P_iP_j}}$$

Although the ratio of expectations does not in general equal the expectation of the ratio, the two are approximately equal in this case because the coefficient of variation is much less than 1 for both the numerator and the denominator. The increase of either *P*_*i*_ or *P*_*j*_ will result in an increase of *E*_*ij*_, which suggests the Jaccard similarity between cells is highly affected by the read depth. Such observation prompts us to develop an ad hoc normalization method to eliminate the read depth effect.

To learn the relationship between the *E*_*ij*_ and *J*_*ij*_ from the data, we next fit a curve to predict the observed Jaccard coefficient *J*_*ij*_ as a function of its expected value *E*_*ij*_ by fitting a polynomials regression of degree 2 using R function lm. Theoretically, *E*_*ij*_ should be linear with *J*_*ij*_ if cells are completely random, but in real dataset, we have observed a nonlinearity between *E*_*ij*_ and *J*_*ij*_ especially among the high-coverage cells. We suspect, to some extent, the degree of randomness of fragment distribution in a single cell is associated with the coverage. To better model the nonlinearity, we include a second order polynomial in our model:

4 $$J_{ij} = \beta _0 + \beta _1E_{ij} + \beta _2E_{ij}^2$$

This fitting provided estimators of parameters $\{ \widehat {\beta _0},\,\widehat {\beta _1},\,\widehat {\beta _2}\}$. As such, we next use it to normalize the observed Jaccard coefficient by:

5 $$N_{ij} = J_{ij}/(\widehat {\beta _0} + \widehat {\beta _1}E_{ij} + \widehat {\beta _2}E_{ij}^2)$$

The fitting of the linear regression, however, can be time consuming with a large matrix. Here we test the possibility of performing this step on a random subset of *y* cells in lieu of the full matrix. When selecting a subset of *y* cells to speed up the first step, we do not select cells at random with a uniform sampling probability. Instead, we set the probability of selecting a cell *i* to

6 $$\frac{1}{{d({\mathrm{log}}_{10}(C_i))}}$$

where d is the density estimate of all log10-transformed cell fragment count and *C*_*i*_ is the number of fragments in cell *i* and $C_i = \mathop {\sum }\nolimits_k^m x_{ik}$. Similar approach was first introduced in SCTranscform^50^ to speed up the normalization of single-cell RNA-seq.

We then proceed to normalize the full Jaccard coefficient matrix ${\mathbf{J}} \in {\mathbf{{\cal{R}}}}^{n \times n}$ using the regression model learned from *y* cells and compared the results to the case where all cells are used in the initial estimation step as well. We use the correlation of normalized Jaccard coefficient to compare this partial analysis to the full analysis. We observe that using as few as 2000 cells in the estimation gave rise to virtually identical estimates. We therefore use 2000 cells in the initial model-fitting step. To remove outliers in the normalized similarity, we use the 0.99 quantile to cap the maximum value of the normalized matrix.

Next, using normalized Jaccard coefficient matrix **N**, we normalize the matrix by:

7 $${\mathbf{A}} = {\mathbf{D}}^{ - 1/2}{\mathbf{ND}}^{ - 1/2}$$

where ${\mathbf{D}} \in {\mathbf{{\cal{R}}}}^{n \times n}$ is a diagonal matrix, which is composed as $D_{i,i} = \mathop {\sum }\nolimits_j N_{i,j}$. We next perform eigenvector decomposition against **A**.

8 $${\mathbf{A}} = {\mathbf{U}}{\mathbf{\Lambda }}{\mathbf{U}}^{\mathrm{T}}$$

The columns ${\mathbf{\varphi }}_{\mathbf{i}} \in {\mathbf{{\cal{R}}}}^n$ of ${\mathbf{U}} \in {\mathbf{{\cal{R}}}}^{n \times n}$ are the eigenvectors. The diagonal matrix ${\mathbf{\Lambda }} \in {\mathbf{{\cal{R}}}}^{n \times n}$ has the eigenvalues $\lambda _1 \ge \lambda _2 \ge \ldots \ge 0$ in descending order as its entries. Finally, we report the first *r* eigenvectors as the final low-dimension manifold.

#### Evaluation of ad hoc normalization method

To assess the performance of normalization of SnapATAC we processed three datasets. As shown in Supplementary Fig. 3, before normalization, SnapATAC exhibits a strong gradient that is correlated with sequencing depth within the cluster (Supplementary Fig. 3a). Although the sequencing depth effect is still observed in some of the small clusters, it is clear that the normalization has largely eliminated the read depth effect as compared to the unnormalized ones (Supplementary Fig. 3b).

To better quantify the coverage bias, we next computed the Shannon entropy that estimates the “uniformness” of the distribution of cell coverage in the UMAP embedding space. In detail, we first chose the top 10% cells of the highest coverage as “high-coverage” cells. Second, in the 2D UMAP embedding space, we discretize “high-coverage” cells from a continuous random coordinate (umap1, umap2) into bins (*n* = 50) and returns the corresponding vector of counts. This is done using a function called “discretize2d” in the “entropy” R package. Third, we estimated the Shannon entropy of the random variable from the corresponding observed counts. This is done using function “entropy” in the “entropy” R package. A higher entropy indicates that the “high-coverage” cells are more uniformly distributed in the UMAP embedding space, overall suggesting a better normalization performance.

We next examine another eight possible sources of biases by projecting to the UMAP embedding space, some metrics show cluster specificity for all three methods perhaps due to biological relevance, but all three methods can reveal significant biological heterogeneity without exhibiting substantial intracluster bias for any metrics examined (Supplementary Fig. 4).

#### Removing batch effects using harmony

When the technical variability is at a larger scale than the biological variability, we apply batch effect corrector—Harmony^24^—to eliminate such confounding factor. Given two datasets generated using different technologies, we first calculate the joint low-dimension manifold as described above. We next apply Harmony to regress out the batch effect, resulting in a new harmonized coembedding. This is implemented as a function “runHarmony” in SnapATAC package.

#### Selection of eigenvector and eigenvalues

We next determine how many eigenvectors to include for the downstream analysis. Here we use an ad hoc approach for choosing the optimal number of components. We look at the scatter plot between every two pairs of eigenvectors and choose the number of eigenvectors that start exhibiting “blob”-like structure in which no obvious biological structure is revealed.

#### Nyström method

The computational cost of the dimensionality reduction scales quadratically with the increase of number of cells. For instance, calculating and normalizing the pairwise kernel Matrix *N* becomes computationally infeasible for large-scale dataset. To overcome this limitation, here we apply Nyström method^21,51^ to calculate the low-dimensional embedding for large-scale dataset.

A Nyström algorithm can be divided into three major steps: (i) sampling: sample a subset of *K* ($K \ll N$) cells from *N* total cells as “landmarks”. Instead of random sampling, here we adopt a density-based sampling approach^50^ to preserve the density distribution of the *N* original points; (ii) embedding: compute the low-dimension embedding for *K* landmarks; (iii) extension: project the remaining *N*–*K* cells onto the low-dimensional embedding as learned from the landmarks to create a joint embedding space for all cells.

This approach significantly reduces the computational complexity and memory usage given that *K* is considerably smaller than *N*. The out-of-sample extension (step iii) further enables projection of new single-cell ATAC-seq datasets to the existing reference single-cell atlas. This allows us to further develop a supervised approach to predict cell types of a new single-cell ATAC-seq dataset based on an existing reference atlas.

A key aspect of this method is the procedure according to which cells are sampled as landmark cells, because different sampled landmark cells give different approximations of the original embedding using full matrix. Here we employ the density-based sampling as described above, which preserves the density distribution of the original points.

Let ${\mathbf{X}} \in {\mathbf{{\cal{R}}}}^{n \times m}$ be a dataset with *n* cells and *m* variables (bins) and ${\mathbf{N}} \in {\mathbf{{\cal{R}}}}^{n \times n}$ be a symmetric kernel matrix calculated using normalized Jaccard coefficient. To avoid calculating the pairwise kernel matrix and performing eigen-decomposition against a big matrix ${\mathbf{N}} \in {\mathbf{{\cal{R}}}}^{n \times n}$, we first sample $k\,(k \ll n)$ landmarks without replacement. This breaks down the original kernel matrix ${\mathbf{N}} \in {\mathbf{{\cal{R}}}}^{n \times n}$ into four components.

9 $${\mathbf{N}} = \left( {\begin{array}{*{20}{c}} {{\mathbf{N}}^{kk}} & {{\mathbf{N}}^{kv}} \\ {{\mathbf{N}}^{vk}} & {{\mathbf{N}}^{vv}} \end{array}} \right)$$

in which ${\mathbf{N}}^{kk} \in {\mathbf{{\cal{R}}}}^{k \times k}$ is the pairwise kernel matrix between *k* landmarks and ${\mathbf{N}}^{vk} \in {\mathbf{{\cal{R}}}}^{(n - k) \times k}$ is the similarity matrix between $(n - k)$ cells and *k* landmarks. Using **N**^*kk*^, we perform dimensionality reduction to obtain the *r*-rank manifold ${\mathbf{U}}^{kk} \in {\mathbf{{\cal{R}}}}^{k \times r}$ as described above.

Using **N**^v*k*^, which estimates the similarity between *n*–*k* cells and *k* landmark cells, we project the rest of *n*–*k* cells to the embedding previously obtained using *k* landmark:

10 $${\mathbf{A}}^{vk} = ({\mathbf{D}}^{vv})^{ - \frac{1}{2}}({\mathbf{N}}^{vk})({\mathbf{D}}^{kk})^{ - \frac{1}{2}}$$

where ${\mathbf{D}}^{vv} \in {\mathbf{{\cal{R}}}}^{(n - k) \times (n - k)}$ is a diagonal matrix which is composed as $D_{i,i}^{vk} = \mathop {\sum }\nolimits_j N_{i,j}^{vk}$. The projected coordinates of the new points onto the r-dimensional intrinsic manifold defined by the landmarks are then given by

11 $${\mathbf{U}}^{vk} = {\mathbf{A}}^{vk}{\mathbf{U}}^{kk}/{\mathbf{\Lambda }}^{kk}$$

The resulting ${\mathbf{U}}^{vk} \in {\mathbf{{\cal{R}}}}^{(n - k) \times r}$ is the approximate *r*-rank low-dimension representation of the rest *n*–*k* cells. Combing ***U***^*kk*^ and ***U***^v*k*^ creates a joint embedding space for all cells:

12 $${\tilde{\mathbf{U}}} = \left[ {\begin{array}{*{20}{c}} {{\mathbf{U}}^{kk}} \\ {{\mathbf{U}}^{vk}} \end{array}} \right]$$

In the approximate joint *r*-rank embedding space ${\tilde{\mathbf{U}}}$, we next create a *k*-nearest neighbor (KNN) graph in which every cell is represented as a node and edges are drawn between cells within *k*-nearest neighbors defined using Euclidean distance. Finally, we apply community finding algorithm such as Louvain (implemented by igraph package in R) to identify the ‘communities’ in the resulting graph, which represents groups of cells sharing similar profiles, potentially originating from the same cell type.

#### Choosing the number of landmarks

To evaluate the effect of the number of landmarks, we apply our method to a complex dataset that contains over 80k cells from 13 different mouse tissues. We employ the following three metrics to evaluate the performance. First, using different number of landmarks (*k*) ranging from 1000 to 10,000, we compare the clustering outcome to the cell-type label defined in the original study. The goal of this is to identify the “elbow” point that performance drops abruptly. Second, for each sampling, we repeat for five times using different set of landmarks to evaluate stability between sampling. Third, we spiked-in 1% Patski cells to assess the sensitivity of identifying rare cell types. We choose Patski cells because these cells were profiled using the same protocol by the same group (Data source listed in Supplementary Table 1) to minimize the batch effect.

We observe that using as few as 5000 landmarks can largely recapitulate the result obtained using 10,000 landmarks (Supplementary Fig. 5a), and 10,000 landmarks can achieve highly robust embedding between sampling (Supplementary Fig. 5b) and successfully recover spiked-in rare populations (Supplementary Fig. 5c). To obtain a reliable low-dimensional embedding, we use 10,000 landmarks for all the analysis performed in this study.

#### Ensemble Nyström method

Nyström is stochastic in its nature, different sampling will result in different embedding and clustering outcome. To improve the robustness of the clustering method, we next employ Ensemble Nyström Algorithm which combines a mixture of Nyström approximation to create an ensemble representation^52^. Supported by theoretical analysis, this Ensemble approach has been demonstrated to guarantee a convergence and in a faster rate in comparison to standard Nyström method^52^. Moreover, this ensemble algorithm naturally fits within distributed computing environments, where their computational costs are roughly the same as that of the standard Nyström single sampling method.

We treat each approximation generated by the Nyström method using *k* landmarks as an expert and combined *p* ≥ 1 such experts to derive an improved approximation, typically more accurate than any of the original experts^52^.

The ensemble setup is defined as follows. Given a dataset ${\mathbf{X}} \in {\mathbf{{\cal{R}}}}^{n \times m}$ of *n* cells. Each expert *S*_*j*_ receives *k* landmarks randomly selected from matrix **X** using density-based sampling approach without replacement. Each expert *S*_*r*_, $r \in [1,p]$ is then used to define the low-dimension embedding ${\tilde{\mathbf{U}}}_{\mathbf{j}} \in {\mathbf{{\cal{R}}}}^{n \times r}$ as described above. For each low-dimension embedding ${\tilde{\mathbf{U}}}_{\mathbf{j}} \in {\mathbf{{\cal{R}}}}^{n \times r}$, we create a KNN-graph as ${\tilde{\mathbf{G}}}_{\mathbf{j}}$. Thus, the general form of the approximation, ${\tilde{\mathbf{G}}}^{{\mathbf{en}}}$, generated by the ensemble Nyström method is

13 $${\tilde{\mathbf{G}}}^{{\mathbf{en}}} = \mathop {\sum }\limits_{j = 1}^p \mu ^j{\tilde{\mathbf{G}}}^{\mathbf{j}}$$

Here we choose to use the most straightforward method by assigning an equal weight to each of the KNN-graph obtained from different samplings, $\mu ^j = 1/p,r \in [1,p]$. While this choice ignores the relative quality of each Nyström approximation, it is computational efficient and already generates a solution superior to any one of the approximations used in the combination. Using the ensemble weighted KNN-graph ${\tilde{\mathbf{G}}}^{{\mathbf{en}}}$, we next apply community finding algorithm to identify cell clusters. By testing on the mouse atlas dataset^8^, we demonstrate that the clustering stability of the ensemble approach is significantly higher than the standard Nyström method (Supplementary Fig. 5d).

#### Visualization

We use the t-SNE implemented by FI-tsne, Rtsne or UMAP (umap_0.2.0.0) to visualize and explore the dataset.

#### Gene accessibility score

To annotate the identified clusters, SnapATAC calculated the gene-body accessibility matrix **G** using “calGmatFromMat” function in SnapATAC packge where *G*_*i,j*_ is the number of fragments overlapping with jth genes in i-th cell. *G*_*i,j*_ is then normalized to CPM (count-per-million reads) as ${\tilde{\mathbf{G}}}$. The normalized accessibility score is then smoothed using Markov affinity-graph-based method:

14 $${\hat{\mathbf{G}}} = {\tilde{\mathbf{GA}}}^t$$

where **A** is the adjacent matrix obtained from *K*-nearest neighbor graph and *t* is number of steps taken for Markov diffusion process. We set *t* = 3 in this study. Please note that the gene accessibility score is only used to guide the annotation of cell clusters identified using cell-by-bin matrix. The clusters are identified using cell-by-bin matrix in prior.

#### Read aggregation and peak calling

After annotation, cells from the same cluster are pooled to create aggregated signal for each of the identified cell types. This allows for identifying *cis*-elements from each cluster. MACS2 (version 2.1.2) is used for generating signal tracks and peak calling with the following parameters: “–nomodel–shift 100–ext 200–qval 1e-2 -B –SPMR”. This can be done by “runMACS” function in SnapATAC package.

#### Motif analysis

SnapATAC incorporates chromVAR^15^ to estimate the motif variability and Homer^22^ for de novo motif discovery. This is implemented as function “runChromVAR” and “runHomer” in SnapATAC package.

#### Identification of differentially accessible peaks

For a given group of cells **C**_**i**_, we first look for their neighboring cells **C**_**j**_ ($|{\mathbf{C}}_{\mathbf{i}}| = |{\mathbf{C}}_{\mathbf{j}}|$) in the low-dimension manifold as “background” cells to compare to. If **C**_**i**_accounts for more than half of the total cells, we use the remaining cells as local background. Next, we aggregate **C**_**i**_ and **C**_**j**_ to create two raw-count vectors as **V**_**ci**_ and **V**_**cj**_. We then perform differential analysis between *V*_**ci**_ and **V**_**cj**_ using exact test as implemented in R package edgeR (v3.18.1) with BCV = 0.1. *P*-value is then adjusted into False Discovery Rate (FDR) using Benjamini–Hochberg correction. Peaks with FDR less than 0.05 are selected as significant DARs.

#### GREAT analysis

SnapATAC incorporates GREAT analysis^27^ to infer the candidate biological pathway active in each cell populations. This is implemented as function “runGREAT” SnapATAC package.

### Integration with single-cell RNA-seq

We use canonical correlation analysis (CCA) embedded in Seurat V3^19^ to integrate single-cell RNA-seq and single-cell ATAC-seq. We first calculate the gene accessibility account at variable genes identified using single-cell RNA-seq dataset. This can be done using a function called “createGmatFromMat” in SnapATAC package. Next, SnapATAC converts the snap object to a Seurat v3 object using a function called “SnapToSeurat” in preparation for integration. Different from integration method in Seurat, we use the low-dimension manifold as the dimensionality reduction method in the Seurat object. We next follow the vignette in Seurat website (https://satijalab.org/seurat/v3.0/atacseq_integration_vignette.html) to integrate these two modalities. The cell type for scATAC-seq is predicted using function “TransferData” in Seurat V3.

Finally, for each single-cell ATAC profile, we infer its gene expression profile by calculating the weighted average expression profile of its nearest neighboring cells in the single-cell RNA-seq dataset^19^. By doing so, we create pseudo-cells that contain information of both chromatin accessibility and gene expression profiles. The imputation of gene expression profile is done by “TransferData” function in Seurat V3.

### Linking enhancers to putative target genes

Using the “pseudo” cells, we next sought to predict the putative target genes for regulatory elements based on the association between expression of a gene and chromatin accessibility at its enhancer elements. Given a gene *G*, we first identify its surrounding regulatory elements within 1MB window flanking *G*. Let **Y**^**G**^ be the imputed gene expression value for gene *G* among *n* cells. We perform logistic regression using **Y**^**G**^ as variable to predict the binary state for each of peaks surrounding *G*. The idea behind using logistic regression is that if there is a relationship between the gene expression (continuous variable) and chromatin accessibility (categorical variable), we should be able to predict chromatin accessibility from the gene expression. Logistic regression does not make many of the key assumptions such as normality of the continuous variables. In addition, since we only have one variable (gene expression) for prediction every time, there is no problem of multicollinearity.

We next fit logistic regression between each of flanking peak and gene expression using “glm” function in R with binomial(link = ‘logit’) as the family function. By doing so, we obtain the regression coefficient *β*_1_ and its corresponding P-value for each peak separately. Here we used 5e-8, a standard *P*-value cutoff for human genome-wise association study to determine the significant association. While this cutoff is less sample or gene specific compared to more complicated methods such as permutation test, it is computational efficient and already generates a reasonable set of gene–enhancer pairings.

To evaluate the performance of our methods, we compare our prediction with *cis*-eQTL derived from interferon-γ and lipopolysaccharide stimulation of monocytes^29^. Significant *cis*-eQTL associations are downloaded from supplementary material in Fairfax (2014)^29^. We filter *cis*-eQTL based on two criteria: (1) only *cis*-eQTLs that overlap with the peaks identified in PBMC dataset are considered; (2) In addition, we only keep the *cis*-eQTLs whose genes overlap with the variable genes determined by scRNA-seq. This filtering reduced the *cis*-eQTL list to 456 candidates.

Next, we estimate the association for each of *cis*-eQTLs by preforming logistic regression test as described above. To make a comparison, we derive a set of negative pairs matched for the distance. The negative control pairs for *cis*-eQTL are chosen in the following manner to control for both distance and chromatin accessibility: for each positive eQTL pair *p*_*ij*_ which connects gene *i* and enhancer *j* with a distance of *d*_*ij*_, we look for the enhancer *k* on the opposite direction of the gene *i* that minimizes | $d_{ij} - d_{iz}$*|*. By doing so, the negative sets are controlled for distance, chromatin accessibility level and gene expression level.

### Simulation of scATAC-seq datasets

First, we download the alignment files (bam files) for ten bulk ATAC-seq experiment from ENCODE (data source listed in Supplementary Table 2). From each bam file, we simulate 1000 single-cell ATAC-seq datasets by randomly down sampling to a variety of coverages ranging from 1000 to 10,000 reads per cells. We next create a cell-by-bin matrix of 5 kb which is used for SnapATAC clustering. Merging peaks identified from each bulk experiment, we create cell-by-peak matrix used for LSA, *Cis*-Topic, Cicero, and chromVAR for clustering. We repeat the sampling for *n* = 10 times to estimate the variability of the clustering.

### Comparison of scalability

To compare the scalability between SnapATAC to other methods, we next simulate multiple datasets of different number of cells ranging from 20k to 1 M. These datasets are simulated in the following manner. Using the 80k mouse atlas dataset, we randomly sample this dataset to different number of cells ranging from 20k t0 1 M cells. For the sampling that has cells more than 80 K, we sample with replacement and introduce perturbation to each cell by randomly removing 1% of the “1”s in each of the cells. This removes the duplicate cells and largely maintains the density of the matrix.

For each sampling, we then perform dimensionality reduction using LSA and *cis*-Topic and compare their CPU running time. Specifically, we monitor the running time for (1) TF-IDF transformation and Singular Value Decomposition (SVD) for LSA, (2) function “runModels” with topics = c(2, 5, 10, 15, 20, 25, 30, 35, 40) and “selectModel” function in *cis*-Topic. The time for matrix loading is not counted.

All the comparisons were tested on a machine with 5 AMD Operon (TM) Processor 6276 CPUs.

### Doublets detection using scrublet

To identify doublets from secondary motor cortex single-nucleus ATAC-seq datasets, we use single-cell RNA-seq doublets detection algorithm Scrublet^37^. Briefly, Scrublet identifies doublets in the following manner: (1) Scrublet performs normalization, gene filtering, and principal components analysis (PCA) to project the high-dimension data to a low-dimension space; (2) Scrublet simulates doublets by adding the unnormalized counts from randomly sampled observed transcriptomes; (3) the simulated doublets are projected to the low-dimension embedding computed in step 1. The more neighbors of a cell are the simulated doublets, the more likely this cell is a “doublet”. Based on this idea, a KNN classifier was then used to estimate the doublet score for each cell.

Since Scrublet was designed for detecting doublets in single-cell RNA-seq, it is unclear whether it can be used for single-cell ATAC-seq. To examine this, we applied Scrublet to a single-cell ATAC-seq dataset of mixed human and mouse cells where the “ground-truth” doublets can be identified based on the alignment ratio to human and mouse genome. Compared to the ground truth, Scrubet can identify over 90% of the doublets in this dataset with ~90% accuracy (Supplementary Fig. 26). This result suggests that although Scrubet was not developed for detecting doublets in single-cell ATAC-seq, it can find the doublets in scATAC-seq dataset with reasonable accuracy and sensitivity.

### Projection of single-cell ATAC-seq datasets to reference atlas

We reason that landmark-extension algorithm can provide a mean to project new single-cell ATAC-seq datasets to a reference atlas. Given a query dataset ${\mathbf{Y}} \in {\mathbf{{\cal{R}}}}^{l \times m}$ that contains *l* query cells with *m* bins and a reference dataset ${\mathbf{X}} \in {\mathbf{{\cal{R}}}}^{n \times m}$ with *n* reference cells of *m* bins. We first randomly sample *k* = 10,000 landmarks from X as described above. Next, we compute the pairwise similarity using normalized Jaccard coefficient for *k* landmarks as ${\mathbf{N}}^{kk} \in {\mathbf{{\cal{R}}}}^{k \times k}$ and obtain the low-dimension manifold ${\mathbf{U}}^k \in {\mathbf{{\cal{R}}}}^{k \times r}$. We then compute ${\mathbf{N}}^{lk} \in {\mathbf{{\cal{R}}}}^{l \times k}$ which estimates the similarity between *l* query cells and *k* landmark cells, and then project the *l* query cells to the embedding precomputed for *k* landmark cells as following:

15 $${\mathbf{A}}^l = ({\mathbf{D}}^l)^{ - \frac{1}{2}}({\mathbf{U}}^k)({\mathbf{D}}^k)^{ - \frac{1}{2}}$$

where ${\mathbf{D}}^l \in {\cal{R}}^{l \times l}$ is a diagonal matrix, which is composed as $D_{i,i}^l = \mathop {\sum }\nolimits_j N_{i,j}^l$ and ${\mathbf{D}}^k \in {\mathbf{{\cal{R}}}}^{k \times k}$ is a diagonal matrix which is composed as $D_{i,i}^k = \mathop {\sum }\nolimits_j N_{i,j}^k$

16 $${\mathbf{U}}^l = {\mathbf{A}}^l{\mathbf{U}}^k/{\mathbf{\Lambda }}^k$$

The resulting ${\mathbf{U}}^l \in {\mathbf{{\cal{R}}}}^{l \times r}$ is the predicted low-dimension manifold for *l* query cells.

In the joint embedding space [${\mathbf{U}}^k,{\mathbf{U}}^l$], we next identify the mutual nearest neighbors between query and landmark cells. For each cell $i_1 \in {\mathbf{X}}^k$ belonging to the landmarks, we find the *k.nearest* (5) cells in the query dataset with the smallest distances to *i*_1_. We do the same for each cell in query cell dataset to find its *k.nearest* (5) neighbors in the landmark dataset. If a pair of cells from each dataset is contained in each other’s nearest neighbors, those cells are considered to be mutual nearest neighbors or MNN pairs (or “anchors”). We interpret these pairs as containing cells that belong to the same cell type or state despite being generated in both landmark and query cells. Thus, any differences between cells in MNN pairs should theoretically represent the nonoverlapping cell types. Here we removed any query cells that failed to identify an MNN pair correspondence in the reference dataset.

To make a classification of the remaining query cells according to the reference dataset, we next apply the neighborhood-based classifier and wish to highlight the pioneering work by Seurat V3^19^. First, we score each anchor (or MNN pair) using shared nearest neighbor (SNN) graph by examining the consistency of edges between cells in the same local neighborhood as described in the original study^19^. Second, we define a weight matrix that estimates the strength of association between each query cell ***c***, and each landmark ***i***. For each query cell ***c***, we identify the nearest ***s*** landmarks in the reference dataset in the joint embedding space. Nearest anchors are then weighted based on their distance to the cell ***c*** over the distance to the *s*-th anchor cell. For each cell *c* and anchor *i*, we compute the weighted similarities as:

17 $$D_{c,i} = \left( {1 - \frac{{{\mathrm{dist}}(c,\,a_i)}}{{{\mathrm{dist}}(c,\,a_s)}}} \right)S_{ai}$$

where ${\mathrm{dist}}(c,i)$ is the Euclidean distance in the joint embedding space and *S*_*ai*_ is the weight for the corresponding MNN pair (anchor). We then normalize the similarity using exponential function:

18 $$\widetilde {D_{c,i}} = 1 - e^{\frac{{ - D_{c,i}}}{{\left( {\frac{2}{{sd}}} \right)^2}}}$$

where sd is set to 1 by default. Finally, we normalize across all ***s*** anchors:

19 $$W_{c,i} = \frac{{\widetilde {D_{c,i}}}}{{\mathop {\sum }\nolimits_1^{j = s} \widetilde {D_{c,j}}}}$$

Here we set s = 50. Please note that the similarity to cells beyond the *s*th anchor neighbor is set to be zero.

Let ${\mathbf{L}} \in {\mathbf{{\cal{R}}}}^{k \times t}$ be the binary label matrix for *k* landmarks with *t* clusters. $L_{i,j} = 1$ indicates the class label for *i*th landmark cell is *j*-th cluster. The row sum of **L** must be 1, suggesting each landmark cell can only be assigned to one cluster label. We then compute label predictions for query cells as ***P***^*l*^:

20 $${\mathbf{P}}^l = {\mathbf{WL}}$$

The resulting ***P***^*l*^ is a probability matrix within 0 and 1, $P_{i,j}^l$ indicates the probability of a cell *i* belong to *j* cluster. Similarly, we infer the t-SNE position of query cells by replacing **L** with t-SNE coordinates of reference points. It is important to note that the distance between cells in the inferred t-SNE coordinate does not neccessarily reflect the cell-to-cell relationship.

### Tissue collection and nuclei isolation

Adult C57BL/6 J male mice were purchased from Jackson Laboratories. Brains were extracted from P56-63 old mice and immediately sectioned into 0.6-mm coronal sections, starting at the frontal pole, in ice-cold dissection media. The secondary motor cortex (MOs) region was dissected from the first three slices along the anterior-posterior axis according to the Allen Brain reference Atlas (http://mouse.brain-map.org/, see Supplementary Fig. 15a for depiction of posterior view of each coronal slice; dashed line highlights the MOs regions on each slice). Slices were kept in ice-cold dissection media during dissection and immediately frozen in dry ice for posterior pooling and nuclei production. For nuclei isolation, the MOs dissected regions from 15–23 animals were pooled, and two biological replicates were processed for each slice. Nuclei were isolated as described in previous studies^53,54^, except no sucrose gradient purification was performed. Flow cytometry analysis of brain nuclei was performed as described in Luo et al.^53^. Animal protocols were approved by the Salk Institute Institutional Animal Care and Use Committee. Animal ethics approval has been obtained for the mouse study.

### Tn5 transposase purification and loading

Tn5 transposase was expressed as an intein chitin-binding domain fusion and purified using an improved version of the method first described by Picelli et al.^55^. T7 Express lysY/I (C3013I, NEB) cells were transformed with the plasmid pTXB1-ecTn5 E54K L372P (#60240, Addgene)^55^. An LB Ampicillin culture was inoculated with three colonies and grown overnight at 37 °C. The starter culture was diluted to an OD of 0.02 with fresh media and shaken at 37 °C until it reached an OD of 0.9. The culture was then immediately chilled on ice to 10 °C and expression was induced by adding 250 μM IPTG (Dioxane Free, CI8280-13, Denville Scientific). The culture was shaken for 4 h at 23 °C after which cells were harvested in 2 L batches by centrifugation, flash frozen in liquid nitrogen and stored at −80 °C. Cell pellets were resuspended in 20 ml of ice-cold lysis buffer (20 mM HEPES 7.2-KOH, 0.8 M NaCl, 1 mM EDTA, 10% Glycerol, 0.2% Triton X-100) with protease inhibitors (Complete, EDTA-free Protease Inhibitor Cocktail Tablets, 11873580001, Roche Diagnostics) and passed three times through a Microfluidizer (lining covered with ice water, Model 110 L, Microfluidics) with a 5 min cool down interval in between each pass. Any remaining sample was purged from the Microfluidizer with an additional 25 ml of ice-cold lysis buffer with protease inhibitors (total lysate volume ~50 ml). Samples were spun down for 20 min in an ultracentrifuge at 125K × *g* (L-80XP, 45 Ti Rotor, Beckman Coulter) at 4 °C. ~45 ml of supernatant was combined with 115 ml ice-cold lysis buffer with protease inhibitors in a cold beaker (total volume = 160 ml) and stirred at 4 °C. 4.2 ml of 10% neutralized polyethyleneimine-HCl (pH 7.0) was then added dropwise. Samples were spun down again for 20 min in an ultracentrifuge at 125K × *g* (L-80XP, 45 Ti Rotor, Beckman Coulter) at 4 °C. The pooled supernatant was loaded onto ~10 ml of fresh Chitin resin (S6651L, NEB) in a chromatography column (Econo-Column (1.5 × 15 cm), Flow Adapter: 7380015, Bio-Rad). The column was then washed with 50–100 ml lysis buffer. Cleavage of the fusion protein was initiated by flowing ~20 ml of freshly made elution buffer (20 mM HEPES 7.2-KOH, 0.5 M NaCl, 1 mM EDTA, 10% glycerol, 0.02% Triton X-100, 100 mM DTT) onto the column at a speed of 0.8 ml/min for 25 min. After the column was incubated for 63 h at 4 °C, the protein was recovered from the initial elution volume and a subsequent 30 ml wash with elution buffer. Protein-containing fractions were pooled and diluted 1:1 with buffer [20 mM HEPES 7.2-KOH,1 mM EDTA, 10% glycerol, 0.5 mM TCEP) to reduce the NaCl concentration to 250 mM. For cation exchange, the sample was loaded onto a 1 ml column HiTrap S HP (17115101, GE), washed with Buffer A (10 mM Tris 7.5, 280 mM NaCl, 10% glycerol, 0.5 mM TCEP) and then eluted using a gradient formed using Buffer A and Buffer B (10 mM Tris 7.5, 1 M NaCl, 10% glycerol, 0.5 mM TCEP) (0% Buffer B over 5 column volumes, 0–100% Buffer B over 50 column volumes, 100% Buffer B over 10 column volumes). Next, the protein-containing fractions were combined, concentrated via ultrafiltration to ~1.5 mg/mL and further purified via gel filtration (HiLoad 16/600 Superdex 75 pg column (28989333, GE)) in Buffer GF (100 mM HEPES-KOH at pH 7.2, 0.5 M NaCl, 0.2 mM EDTA, 2 mM DTT, 20% glycerol). The purest Tn5 transposase-containing fractions were pooled and 1 volume 100% glycerol was added to the preparation. Tn5 transposase was stored at −20 °C.

To generate Tn5 transposomes for combinatorial barcoding assisted single-nucleus ATAC-seq, barcoded oligos were first annealed to pMENTs oligos (95 °C for 5 min, cooled to 14 °C at a cooling rate of 0.1 °C/s) separately. Next, 1 µl barcoded transposon (50 µM) was mixed with 7 ul Tn5 (~7 µM). The mixture was incubated on the lab bench at room temperature for 30 min. Finally, T5 and T7 transposomes were mixed in a 1:1 ratio and diluted 1:10 with dilution buffer (50% Glycerol, 50 mM Tris-HCl (pH = 7.5), 100 mM NaCl, 0.1 mM EDTA, 0.1% Triton X-100, 1 mM DTT). For combinatorial barcoding, we used eight different T5 transposomes and 12 distinct T7 transposomes, which eventually resulted in 96 Tn5 barcode combinations per sample^7^ (Supplementary Table 4). Library quality control for single-nucleus ATAC-seq can be found in Supplementary Table 3.

### Bulk ATAC-seq data generation and analysis

ATAC-seq was performed on 30,000–50,000 nuclei. Nuclei were thawed on ice and pelleted for 5 min at 500 × *g* at 4 °C. Nuclei pellets were resuspended in 30 µl tagmentation buffer (36.3 mM Tris-acetate (pH = 7.8), 72.6 mM K-acetate, 11 mM Mg-acetate, 17.6% DMF) and counted on a hemocytometer. 30,000–50,000 nuclei were used for tagmentation and the reaction volume was adjusted to 19 µl using tagmentation buffer. After addition of 1 µl TDE1 (Illumina FC-121-1030), tagmentation was performed at 37 °C for 60 min with shaking (500 rpm). Tagmented DNA was purified using MinElute columns (Qiagen), PCR-amplified for eight cycles with NEBNext® High-Fidelity 2X PCR Master Mix (NEB, 72 °C 5 min, 98 °C 30 s, [98 °C 10 s, 63 °C 30 s, 72 °C 60 s] × 8 cycles, 12 °C held). Amplified libraries were purified using MinElute columns (Qiagen) and SPRI Beads (Beckmann Coulter). Sequencing was carried out on a NextSeq500 using a 150-cycle kit (75 bp PE, Illumina).

Bulk ATAC-seq reads were mapped to reference genome mm10 using BWA and samtools version 1.2 to eliminate PCR duplicates and mitochondrial reads. The paired-end read ends were converted to fragments. Using fragments, MACS2^26^ version 2.1.2 was used for generating signal tracks and peak calling with the following parameters:–nomodel–shift 100–ext 200–qval 1e-2 -B –SPMR. Library quality control for bulk ATAC-seq can be found in Supplementary Table 5.

### Single-nucleus ATAC-seq data generation

Combinatorial barcoding single nucleus ATAC-seq was performed as described previously with modifications^5,7^. In detail, for each sample two biological replicates were processed. Nuclei were pelleted with a swinging bucket centrifuge (500 × *g*, 5 min, 4 °C; 5920 R, Eppendorf). Nuclei pellets were resuspended in 1 ml nuclei permeabilization buffer (5% BSA, 0.2% IGEPAL-CA630, 1 mM DTT and cOmpleteTM, EDTA-free protease inhibitor cocktail (Roche) in PBS) and pelleted again (500 × *g*, 5 min, 4 °C; 5920 R, Eppendorf). Nuclei were resuspended in 500 µL high salt tagmentation buffer (36.3 mM Tris-acetate (pH = 7.8), 72.6 mM potassium-acetate, 11 mM Mg-acetate, 17.6% DMF) and counted using a hemocytometer. Concentration was adjusted to 4500 nuclei/9 µl, and 4500 nuclei were dispensed into each well of a 96-well plate. Glycerol was added to the leftover nuclei suspension for a final concentration of 25% and nuclei were stored at −80 °C. For tagmentation, 1 µL barcoded Tn5 transposomes^7,55^ (Supplementary Table 4) were added using a BenchSmart™ 96 (Mettler Toledo), mixed five times and incubated for 60 min at 37 °C with shaking (500 rpm). To inhibit the Tn5 reaction, 10 µL of 40 mM EDTA were added to each well with a BenchSmart™ 96 (Mettler Toledo) and the plate was incubated at 37 °C for 15 min with shaking (500 rpm). Next, 20 µL 2 × sort buffer (2% BSA, 2 mM EDTA in PBS) were added using a BenchSmart™ 96 (Mettler Toledo). All wells were combined into a FACS tube and stained with 3 µM Draq7 (Cell Signaling) (Supplementary Fig. 27). Using a SH800 (Sony), 20 nuclei were sorted per well into eight 96-well plates (total of 768 wells) containing 10.5 µL EB (25 pmol primer i7, 25 pmol primer i5, 200 ng BSA (Sigma)^7^. Preparation of sort plates and all downstream pipetting steps were performed on a Biomek i7 Automated Workstation (Beckman Coulter). After addition of 1 µL 0.2% SDS, samples were incubated at 55 °C for 7 min with shaking (500 rpm). We added 1 µL 12.5% Triton-X to each well to quench the SDS and 12.5 µL NEBNext High-Fidelity 2× PCR Master Mix (NEB). Samples were PCR-amplified (72 °C 5 min, 98 °C 30 s, (98 °C 10 s, 63 °C 30 s, 72 °C 60 s) × 12 cycles, held at 12 °C). After PCR, all wells were combined. Libraries were purified according to the MinElute PCR Purification Kit manual (Qiagen) using a vacuum manifold (QIAvac 24 plus, Qiagen) and size selection was performed with SPRI Beads (Beckmann Coulter, 0.55× and 1.5×). Libraries were purified one more time with SPRI Beads (Beckmann Coulter, 1.5×). Libraries were quantified using a Qubit fluorimeter (Life technologies) and the nucleosomal pattern was verified using a Tapestation (High Sensitivity D1000, Agilent). The library was sequenced on a HiSeq2500 sequencer (Illumina) using custom sequencing primers, 25% spike-in library and following read lengths: 50 + 43 + 40 + 50 (Read1 + Index1 + Index2 + Read2)^7^.

### Reporting summary

Further information on research design is available in the Nature Research Reporting Summary linked to this article.

## Supplementary information

Supplementary Infomation

Supplementary Data 1

Reporting Summary

## Data Availability

Raw and processed data generated in this study have been deposited to NCBI Gene Expression Omnibus with the accession number GSE126724. Any data that support the findings of this study beyond what is included in the Supplementary Information are available from the corresponding author upon request. Please note that the information on where previously published data used within this study can be found in Supplementary Table 1. Source data are provided with this paper.

## Source data

Source Data

## Code availability

The scripts and pipeline for the analysis can be found at https://github.com/r3fang/SnapATAC.
