# Supplementary material for: Comprehensive analysis of single cell ATAC-seq data with SnapATAC
Source: Nat Commun. 2021 Feb 26;12:1337. doi: 10.1038/s41467-021-21583-9 (PMC7910485; doi:10.1038/s41467-021-21583-9)
Supplement: Supplementary file 1 — Supplementary Infomation [file 41467_2021_21583_MOESM1_ESM.pdf]

Comprehensive analysis of single cell ATAC-seq data with SnapATAC

Rongxin Fang[1,2], Sebastian Preissl[3], Yang Li[1], Xiaomeng Hou[3], Jacinta Lucero[4], Xinxin Wang[3], Amir Motamedi[5], Andrew K. Shiau[5], Xinzhu Zhou[6], Fangming Xie[7], Eran A. Mukamel[7], Kai Zhang[1], Yanxiao Zhang[1], M. Margarita Behrens[4], Joseph R. Ecker[4,8], and Bing Ren[1,3,9*]

1. Ludwig Institute for Cancer Research, La Jolla, CA 92093, USA
2. Department of Chemistry and Chemical Biology, Harvard University, Cambridge, MA 02138, USA
3. Center for Epigenomics, Department of Cellular and Molecular Medicine, University of California, San Diego, La Jolla, CA 92093, USA
4. The Salk Institute for Biological Studies, La Jolla, CA 92037, USA
5. Small Molecule Discovery Program, Ludwig Institute for Cancer Research, La Jolla, CA 92093, USA
6. Biomedical Science Graduate Program, University of California San Diego, La Jolla, CA 92093, USA
7. Department of Cognitive Science, University of California, San Diego, La Jolla, CA 92037, USA
8. Howard Hughes Medical Institute, The Salk Institute for Biological Studies, La Jolla, CA 92037, USA
9. Department of Cellular and Molecular Medicine, Institute of Genomic Medicine, UCSD Moores Cancer Center, La Jolla, CA 92093, USA

*Correspondence to: biren@ucsd.edu

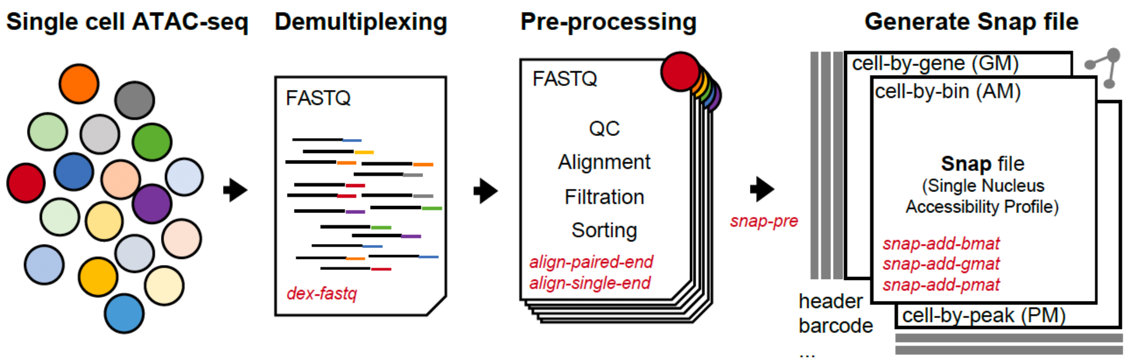

Supplementary Fig. 1. SnapTools preprocessing workflow. Demultiplexing: SnapTools first de-multiplexes the fastq files by adding the cell barcodes to the beginning of each read name; Pre-processing: raw sequencing reads are aligned to the reference genome using BWA[1] followed by filtration of erroneous alignments. A snap file is generated to store indexed reads and multiple cell matrices including cell-by-peak, cell-by-gene and cell-by-bin matrix for subsequent analysis.

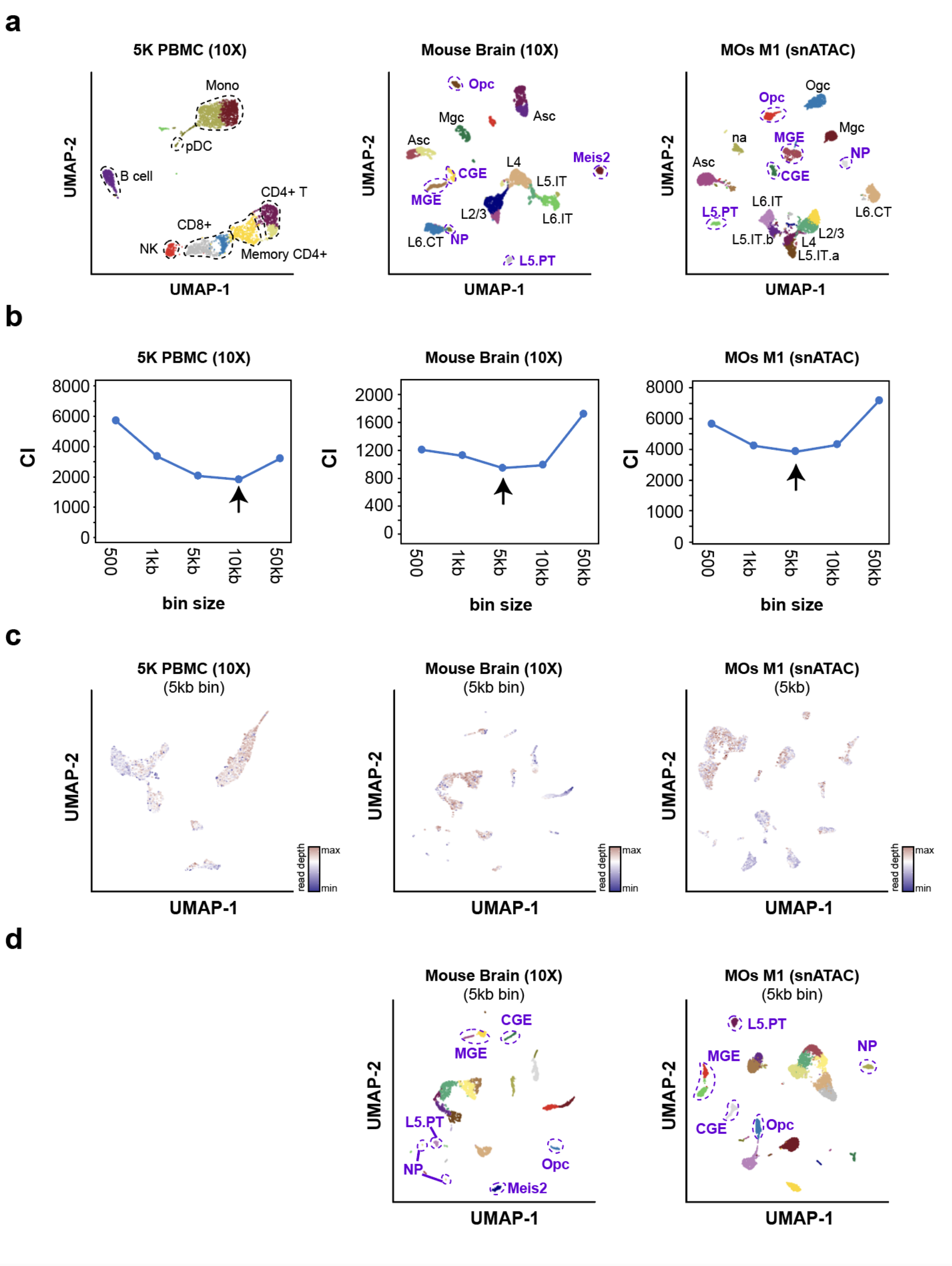
a
5K PBMC (10X)
Mono
pDC
B cell
CD4+ T
CD8+
NK
Memory CD4+
UMAP-1
UMAP-2
Mouse Brain (10X)
Opc
Asc
Mgc
Asc
Meis2
CGE
L4
L5.IT
MGE
L2/3
L6.IT
L6.CT
NP
L5.PT
MOs M1 (snATAC)
Opc
Ogc
na
MGE
Mgc
NP
Asc
CGE
L6.IT
L5.PT
L6.CT
L2/3
L5.IT.b
L4
L5.IT.a
b
5K PBMC (10X)
Mouse Brain (10X)
MOs M1 (snATAC)
CI
bin size
500
1kb
5kb
10kb
50kb
8000
6000
4000
2000
1000
0
2000
1600
1200
800
400
c
5K PBMC (10X)
(5kb bin)
Mouse Brain (10X)
(5kb bin)
MOs M1 (snATAC)
(5kb)
read depth
max
min
d
Mouse Brain (10X)
(5kb bin)
CGE
MGE
L5.PT
NP
Opc
Meis2
MOs M1 (snATAC)
(5kb bin)
L5.PT
NP
MGE
CGE
Opc

Supplementary Fig. 2. Choosing the optimal bin size. a UMAP visualization of landmark cell types identified in three benchmarking datasets (Methods section). UMAP embedding was computed using cisTopic[2] and cell types were manually annotated based on the gene accessibility score at canonical marker genes (Methods section). Blue dash line highlights the annotated rare cell populations that account for less than 2% of the total population. **b** Relationship between connectivity index (CI) and bin sizes. Connectivity index were calculated between landmark cell types in the low dimension manifold using function "connectivity" in R package "clv". A lower CI indicates a better separation of landmark cell types. **c** UMAP representation of three benchmarking datasets generated using SnapATAC with bin size of 5kb. Cells colored by read depth to illustrate the sequencing depth effect. **d** Cells are colored by cluster labels identified by SnapATAC. Data source is listed in Supplementary Table 1.

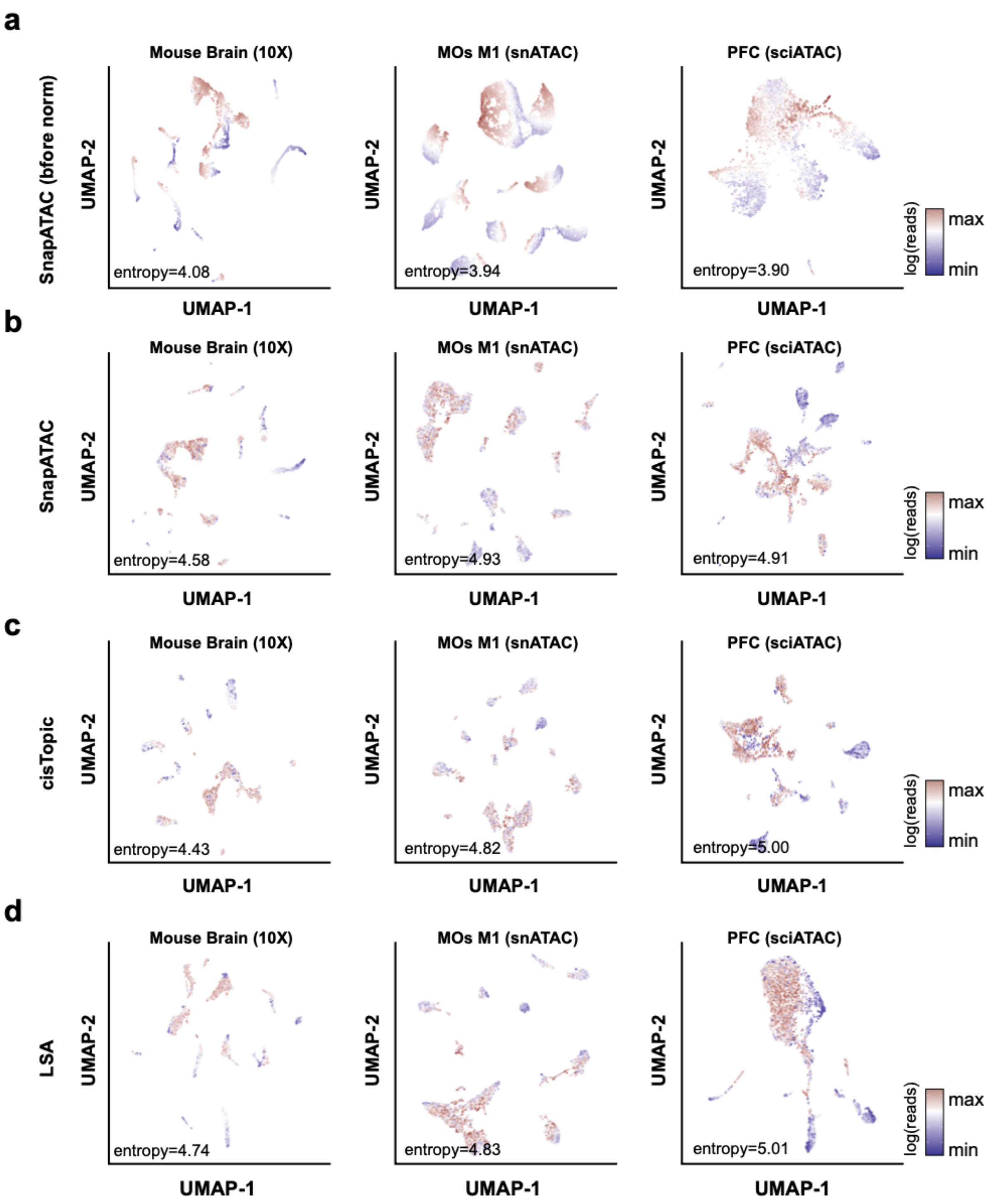
a
SnapATAC (bfore norm)
Mouse Brain (10X)
entropy=4.08
MOs M1 (snATAC)
entropy=3.94
PFC (sciATAC)
entropy=3.90
UMAP-1
UMAP-2
log(reads)
max
min
b
SnapATAC
Mouse Brain (10X)
entropy=4.58
MOs M1 (snATAC)
entropy=4.93
PFC (sciATAC)
entropy=4.91
c
cisTopic
Mouse Brain (10X)
entropy=4.43
MOs M1 (snATAC)
entropy=4.82
PFC (sciATAC)
entropy=5.00
d
LSA
Mouse Brain (10X)
entropy=4.74
MOs M1 (snATAC)
entropy=4.83
PFC (sciATAC)
entropy=5.01

Supplementary Fig. 3. SnapATAC is robust to sequencing depth. Two dimensional UMAP representation of three benchmarking datasets analyzed by four methods (**a**) SnapATAC without normalization; (**b**) SnapATAC with normalization; (**c**) cisTopic and (**d**) Latent Sematic Analysis (LSA)[3]. Cells are colored by log-scaled read depth. Read depth bias is quantified by entropy as described in the Methods section. Data source is listed in Supplementary Table 1.

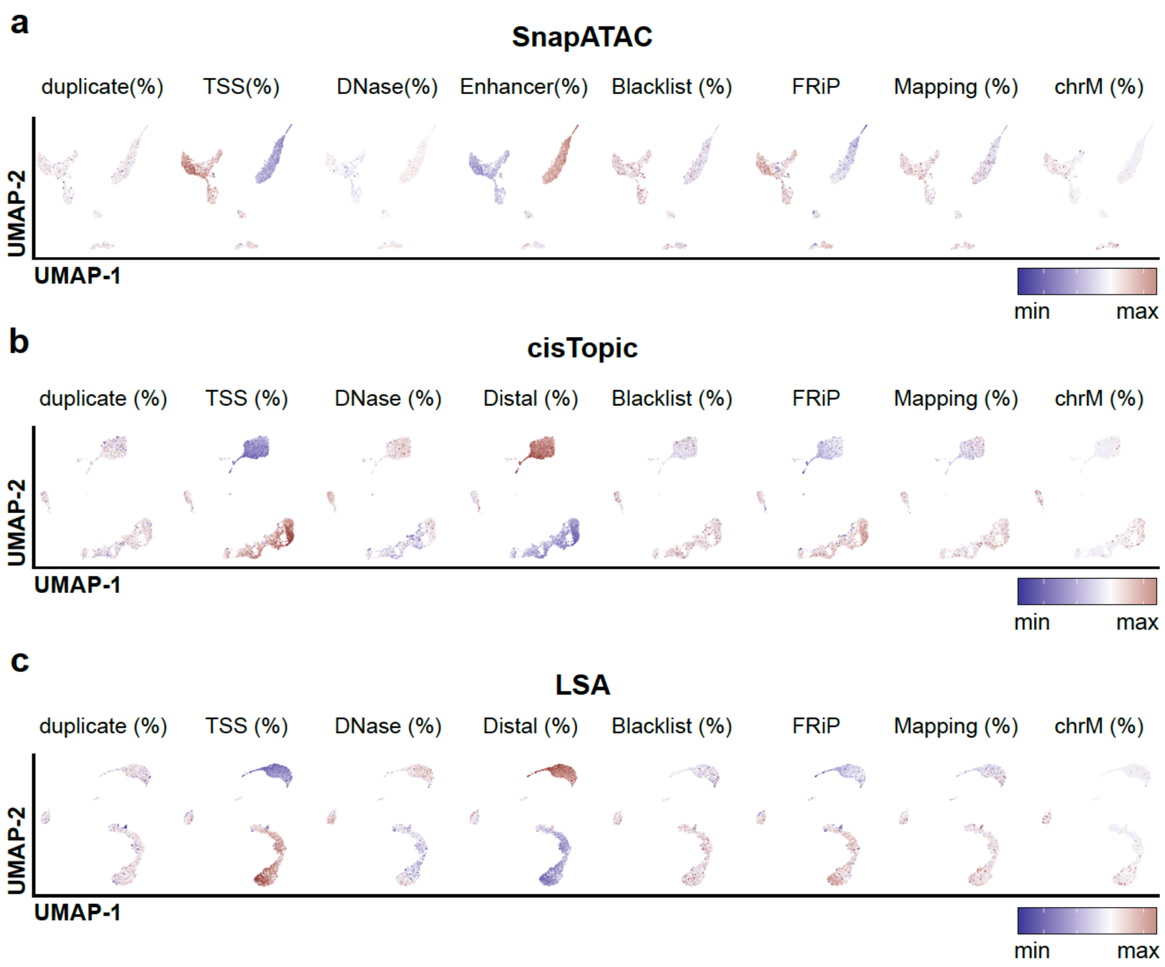

Supplementary Fig. 4. SnapATAC is robust to multiple sources of potential biases. Potential biases in single cell ATAC-seq dataset are projected onto the UMAP visualization generated using different analysis methods (**a**) SnapATAC (**b**) cisTopic[2] and (**c**) LSA[3]. Duplicate: percentage of fragments that are PCR duplicates. TSS: percentage of fragments overlapping or are within 1kb of a TSS. TSS position is based on the GENECODE V28 (Ensemble 92). DNase: the percentage of fragments overlapping a master DNase peak list. The DNase peak list is created by combining all ENCODE DNase peaks from hg19. Blacklist: the percentage of fragments overlapping with the ENCODE blacklist. FRiP: the percentage of fragments overlapping with the peaks defined from the aggregate signal. Mapping: the percentage of fragments that are uniquely mapped. chrM: the percentage of fragments mapped to mitochondria DNA. The source of the dataset used in this plot (5k PBMC 10X) is listed in Supplementary Table 1.

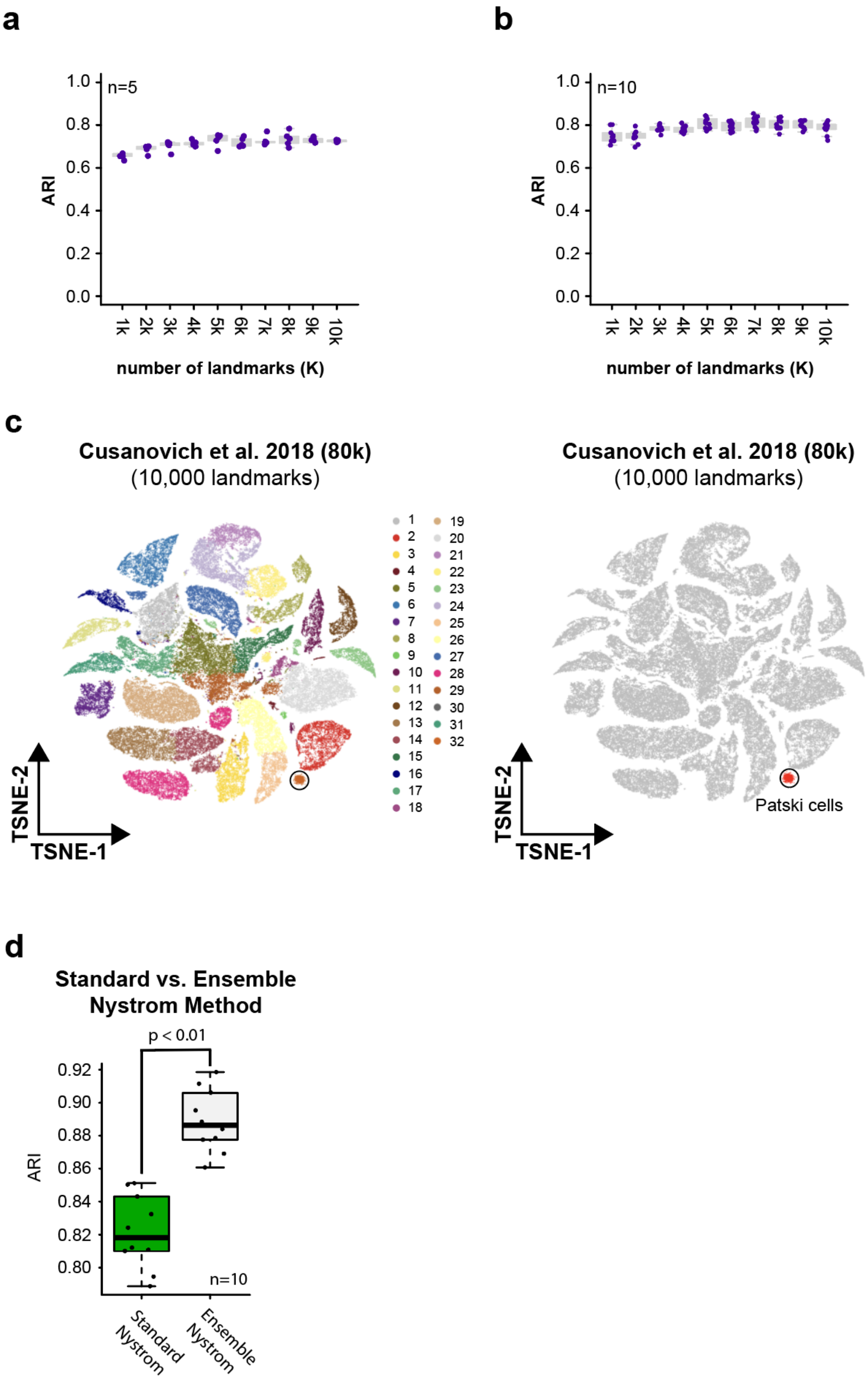
a
n=5
ARI
1.0
0.8
0.6
0.4
0.2
0.0
1k 2k 3k 4k 5k 6k 7k 8k 9k 10k
number of landmarks (K)
b
n=10
ARI
number of landmarks (K)
c
Cusanovich et al. 2018 (80k)
(10,000 landmarks)
1 2 3 4 5 6 7 8 9 10 11 12 13 14 15 16 17 18 19 20 21 22 23 24 25 26 27 28 29 30 31 32
TSNE-2
TSNE-1
Patski cells
d
Standard vs. Ensemble
Nystrom Method
p < 0.01
ARI
0.92
0.90
0.88
0.86
0.84
0.82
0.80
n=10
Standard Nystrom
Ensemble Nystrom

Supplementary Fig. 5. Ensemble Nyström method improves the scalability and stability without sacrificing the performance. a SnapATAC was applied to the single cell ATAC-seq dataset[3] that contained over 80k cells using different number of landmark cells (*k*) ranging from 1k to 10k. For each *k*, we performed clustering for n=5 times using different sets of randomly selected landmarks. A box plot comparing the performance of clustering using different *k*. The performance is evaluated using Adjusted Rank Index (ARI). Data are presented as boxplot as median values +/- 25% percentile**. b** A box plot comparing the stability of clustering results between five samplings (pairwise comparison n=10). Data are presented as boxplot as median values +/- 25% percentile**. c** To evaluate the sensitivity of identifying rare cell types, we spiked in 1% mouse Pastki cells generated using the same protocol in Cusanovich 2015[4] and this rare cell population was recapitulated using 10,000 landmarks (right). **d** To compare the clustering reproducibility between standard and ensemble Nyström[5] sampling method, we performed clustering using both methods on Cusanovich 2018[3] for five times with different randomly selected landmark cells. The clustering reproducibility quantified by ARI (adjusted rank index) between random trails is significantly higher for the ensemble Nyström method than the standard Nyström method (two-tailed t-test p = 1e-4). Data are presented as 1.5 times interquartile range below 25% percentile, 25% percentile, 50% percentile, 75% percentile and 1.5 times interquartile range above 75% percentile.

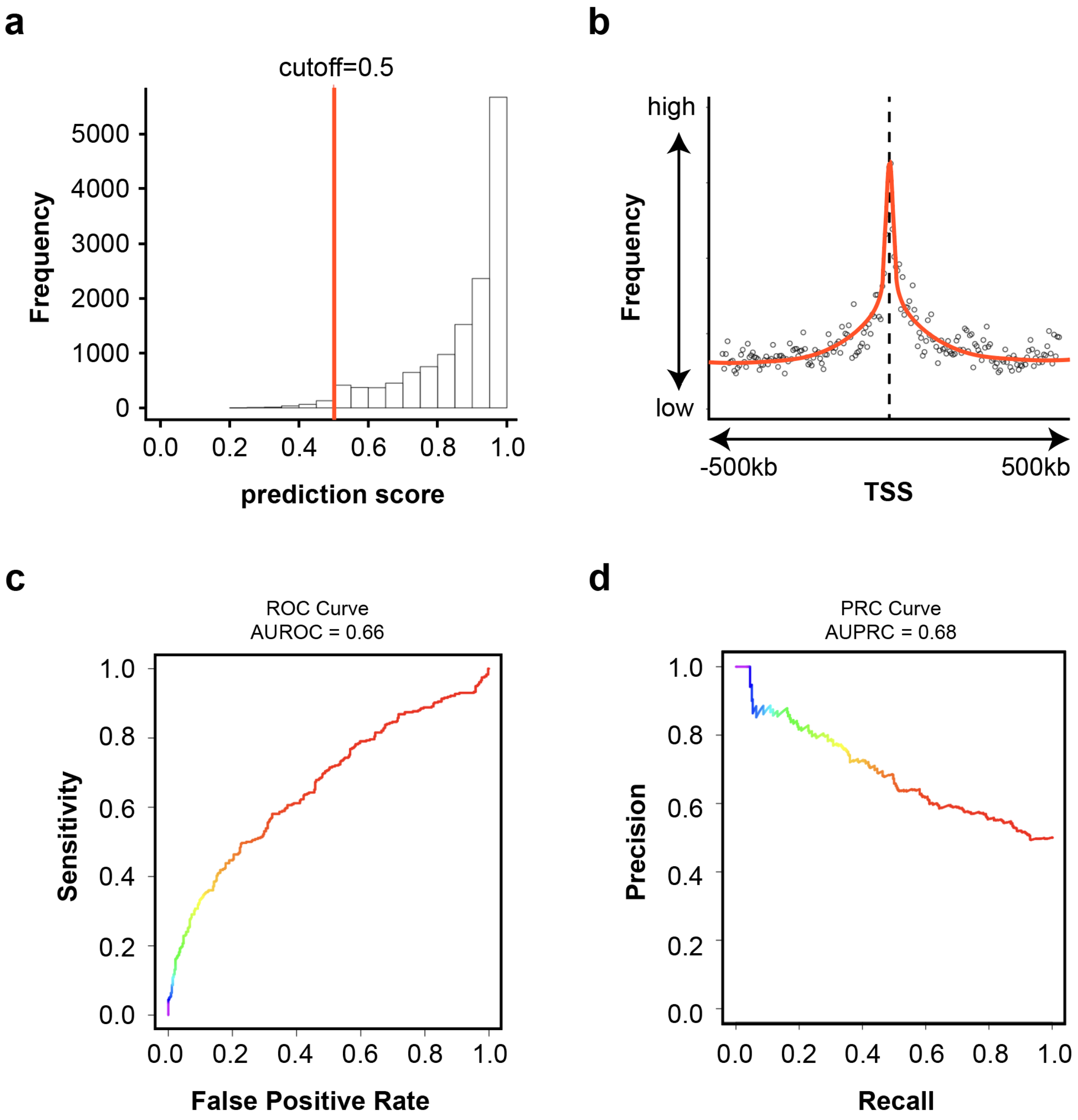
a
cutoff=0.5
Frequency
5000
4000
3000
2000
1000
0
0.0 0.2 0.4 0.6 0.8 1.0
prediction score
b
high
Frequency
low
-500kb
500kb
TSS
c
ROC Curve
AUROC = 0.66
Sensitivity
False Positive Rate
1.0 0.8 0.6 0.4 0.2 0.0
0.0 0.2 0.4 0.6 0.8 1.0
d
PRC Curve
AUPRC = 0.68
Precision
Recall
1.0 0.8 0.6 0.4 0.2 0.0
0.0 0.2 0.4 0.6 0.8 1.0

Supplementary Fig. 6. SnapATAC predicts gene and enhancer pairing by integrating scATAC-seq and scRNA-seq. a Distribution of cell type prediction score for single cell ATAC-seq (5K PBMC 10X) using matched single cell RNA-seq. When predicting the cell type for scATAC-seq using corresponding scRNA-seq dataset (10X PBMC scRNA-seq), each cell in scATAC-seq was assigned with a prediction score indicating the confidence of the prediction. It ranges from 0 to 1, a higher score indicates a higher confidence. Using 0.5 as cutoff as suggested in Seurat[6], over 98% of cells in scATAC-seq are confidently assigned to a cell type defined in scRNA-seq. **b** Distance decay curve for the association (-logPvalue) between regulatory elements and the TSS of their putative target genes. (**c-d**) AUROC and AUPRC between cis-eQTL pairs and negative control sets. See Methods section for how the control sets are selected.

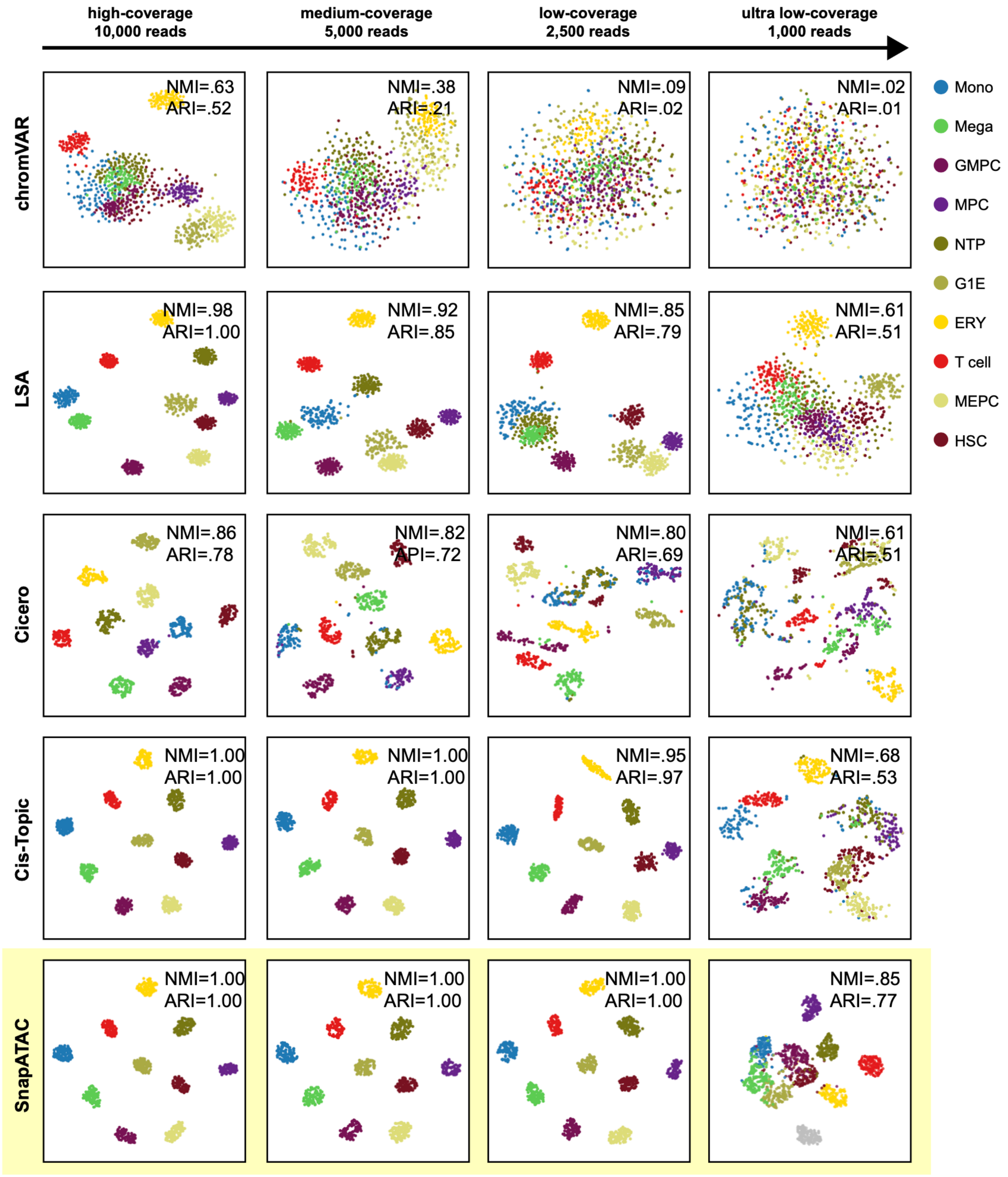

high-coverage
10,000 reads
medium-coverage
5,000 reads
low-coverage
2,500 reads
ultra low-coverage
1,000 reads
chromVAR
NMI=.63
ARI=.52
NMI=.38
ARI=.21
NMI=.09
ARI=.02
NMI=.02
ARI=.01
LSA
NMI=.98
ARI=1.00
NMI=.92
ARI=.85
NMI=.85
ARI=.79
NMI=.61
ARI=.51
Cicero
NMI=.86
ARI=.78
NMI=.82
ARI=.72
NMI=.80
ARI=.69
NMI=.61
ARI=.51
Cis-Topic
NMI=1.00
ARI=1.00
NMI=1.00
ARI=1.00
NMI=.95
ARI=.97
NMI=.68
ARI=.53
SnapATAC
NMI=1.00
ARI=1.00
NMI=1.00
ARI=1.00
NMI=1.00
ARI=1.00
NMI=.85
ARI=.77
Mono
Mega
GMPC
MPC
NTP
G1E
ERY
T cell
MEPC
HSC

Supplementary Fig. 7. Evaluation of clustering accuracy of SnapATAC relative to alternative methods on simulated datasets. T-SNE visualization of clustering results on 1,000 simulated cells sampled from 10 bulk ATAC-seq datasets (see Methods section for the simulation) analyzed by five different methods – chromVAR[7], LSA[3], Cicero[8], Cis-Topic[2] and SnapATAC. Clustering results are compared to the original cell type label and the accuracy is estimated using Normalized Mutual Index (nmi). Mono: monocyte; Mega: megakaryocyte; GMPC: granulocyte monocyte progenitor cell; MPC: megakaryocyte progenitor cell; NPT: neutrophil; G1E: G1E; T cell: regulatory T cell; MEPC: megakaryocyte-erythroid progenitor cell; HSC: hematopoietic stem cell.

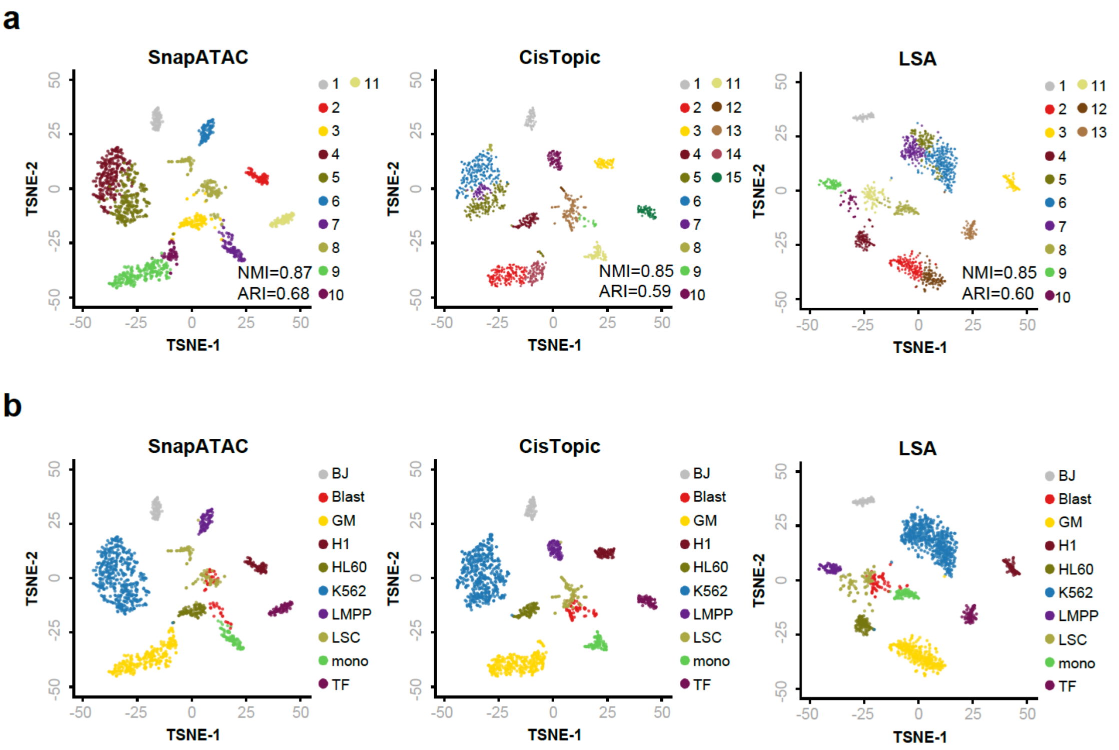

Supplementary Fig. 8. Evaluation of clustering accuracy relative to alternative methods on published single cell ATAC-seq datasets. SnapATAC (left), CisTopic (middle) and LSA (right) clustering performance on single cell ATAC-seq dataset from ten human cell lines generated using Fluidigm C1 platform[7,9]. **a** Clustering results are visualized using t-SNE and cells are colored by cluster labels identified by each of analysis methods. **b** T-SNE visualization of the human cells colored by the cell type labels. Clustering accuracy of each method is estimated by comparing the predicted clustering labels to the cell type labels. Blast: acute myeloid leukemia blast cells; LSC: acute myeloid leukemia leukemic stem cells; LMPP: lymphoid-primed multipotent progenitors; Mono: monocyte; HL60: HL-60 promyeloblast cell line; TF1: TF-1 erythroblast cell line; GM: GM12878 lymphoblastoid cell line; BJ: human fibroblast cell line; H1: H1 human embryonic stem cell line.

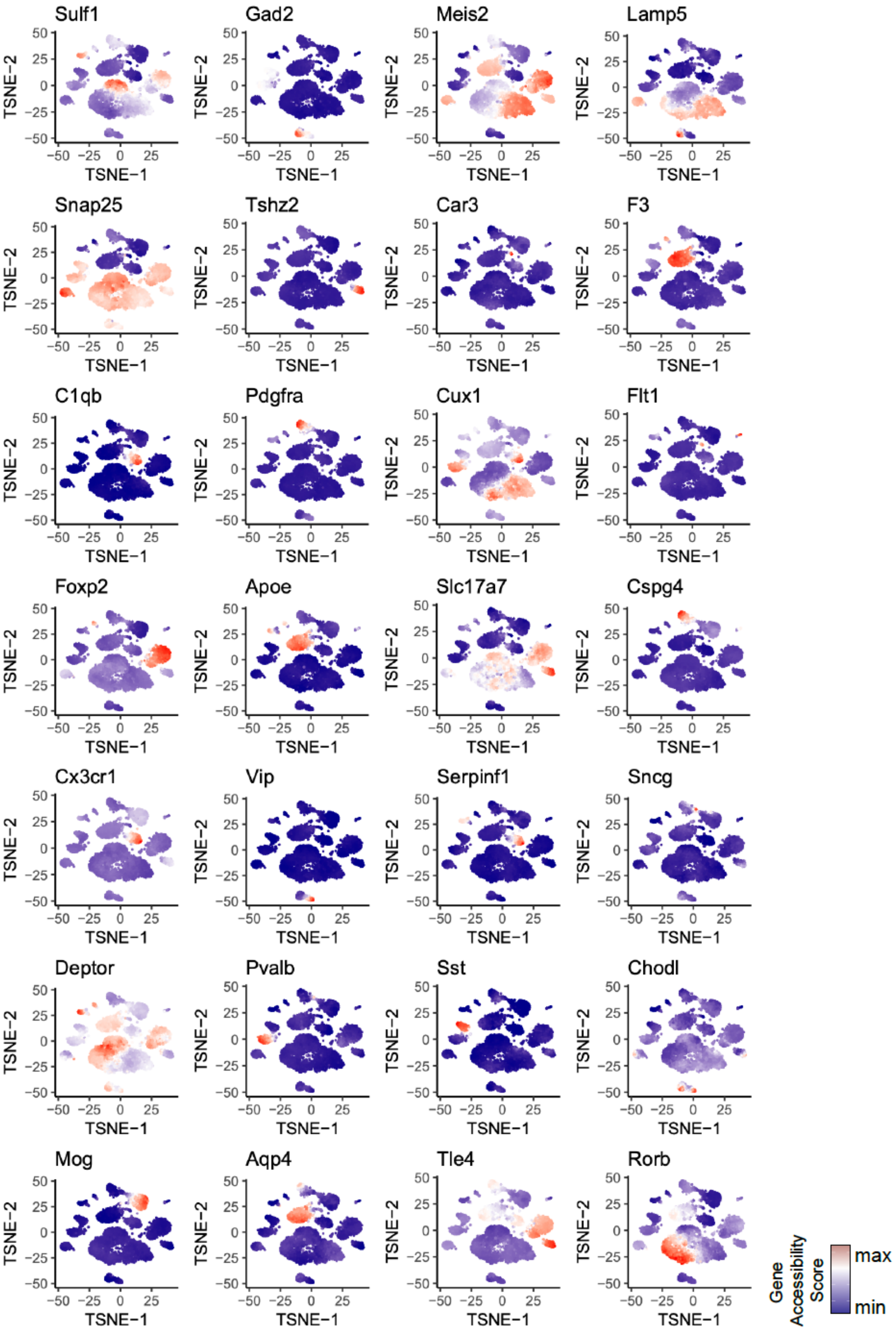

Sulf1
Gad2
Meis2
Lamp5
Snap25
Tshz2
Car3
F3
C1qb
Pdgfra
Cux1
Flt1
Foxp2
Apoe
Slc17a7
Cspg4
Cx3cr1
Vip
Serpinf1
Sncg
Deptor
Pvalb
Sst
Chodl
Mog
Aqp4
Tle4
Rorb
TSNE-1
TSNE-2
Gene Accessibility Score
max
min

Supplementary Fig. 9. Gene accessibility score of canonical marker genes projected onto t-SNE embedding for snATAC-seq dataset from mouse secondary motor cortex. T-SNE is generated using SnapATAC; cell type specific marker genes were defined from previous single cell transcriptomic analysis in the adult mouse brain[10]; gene accessibility score is calculated using SnapATAC (Methods section). Data source is listed in Supplementary Table 1.

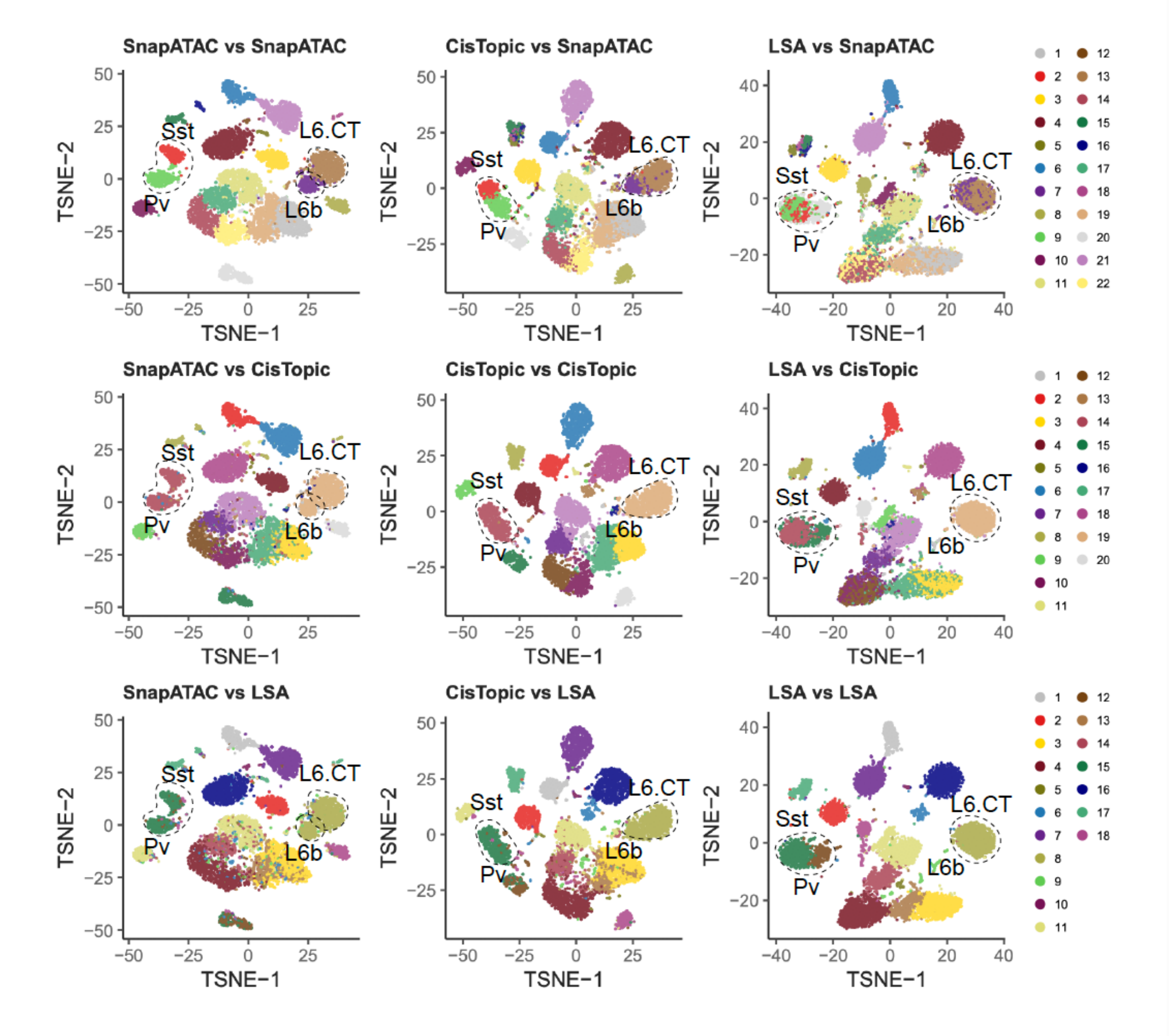

Supplementary Fig. 10. *Ad hoc* evaluation of clustering sensitivity of SnapATAC relative to alternative methods on mouse secondary motor cortex snATAC-seq. Three methods (cisTopic, LSA and SnapATAC) were used to analyze a dataset that contained ~10k single nucleus ATAC-seq profiles from the mouse secondary motor cortex generated in this study. Pairwise comparison of the clustering results is shown by projecting the cluster label identified using one method onto the t-SNE visualization generated by another method (cluster vs. visualization). Black dash line circles highlight the rare pollutions (Sst, Pv, L6b and L6.CT) that were only identified by SnapATAC. Data source is listed in Supplementary Table 1.

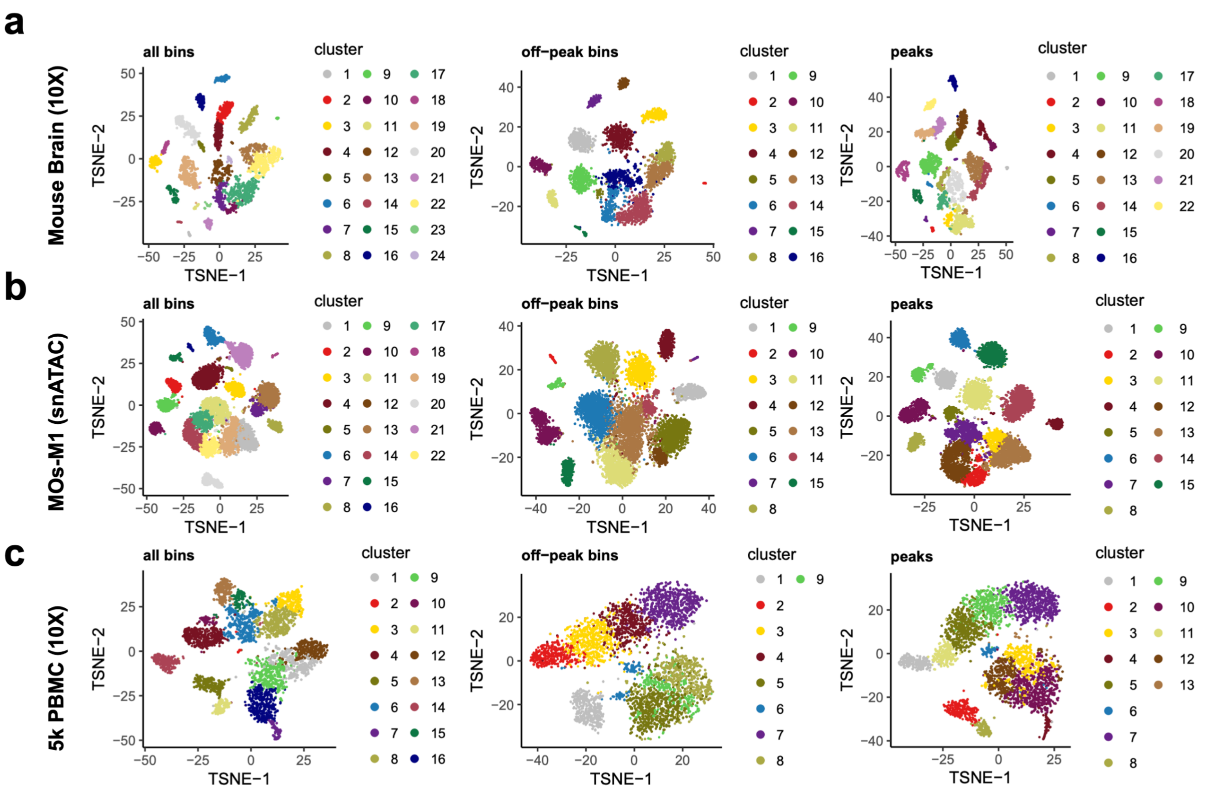

Supplementary Fig. 11. Off-peak reads distinguish major cell types in heterogenous samples. (**a-c**) SnapATAC clustering result on three benchmarking datasets using all bins versus clustering result only using bins that are not overlapped with peaks. Data source is listed in Supplementary Table 1.

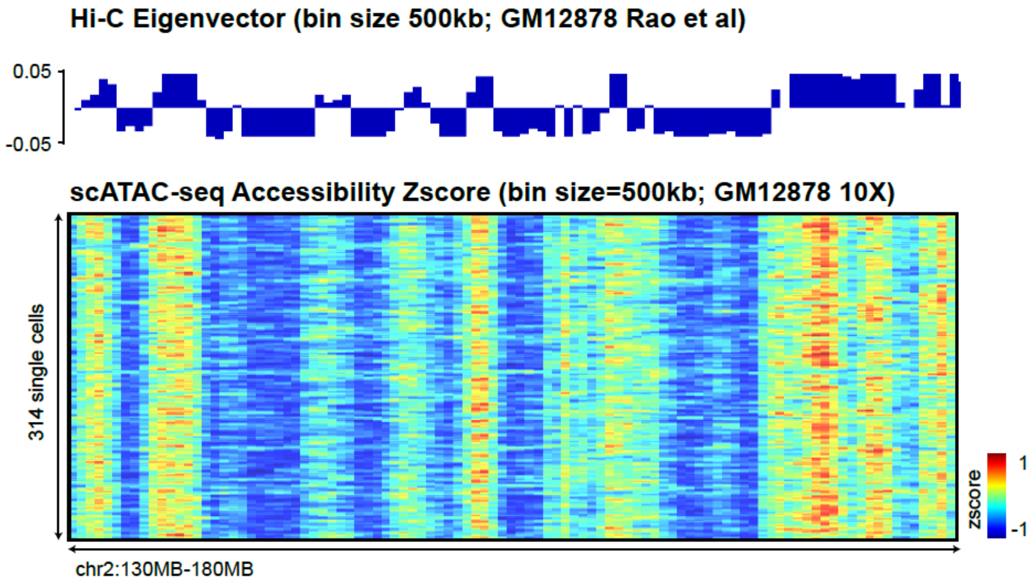

Supplementary Fig. 12. Off-peak reads reflect higher-order chromatin structure. At 500kb bin resolution, profile of compartments identified using Hi-C[11] in GM12878 overlaid the density of "off-peak" reads for 314 cells from GM12878 10X scATAC-seq library. Source of the data used for generating this plot is listed in Supplementary Table 1.

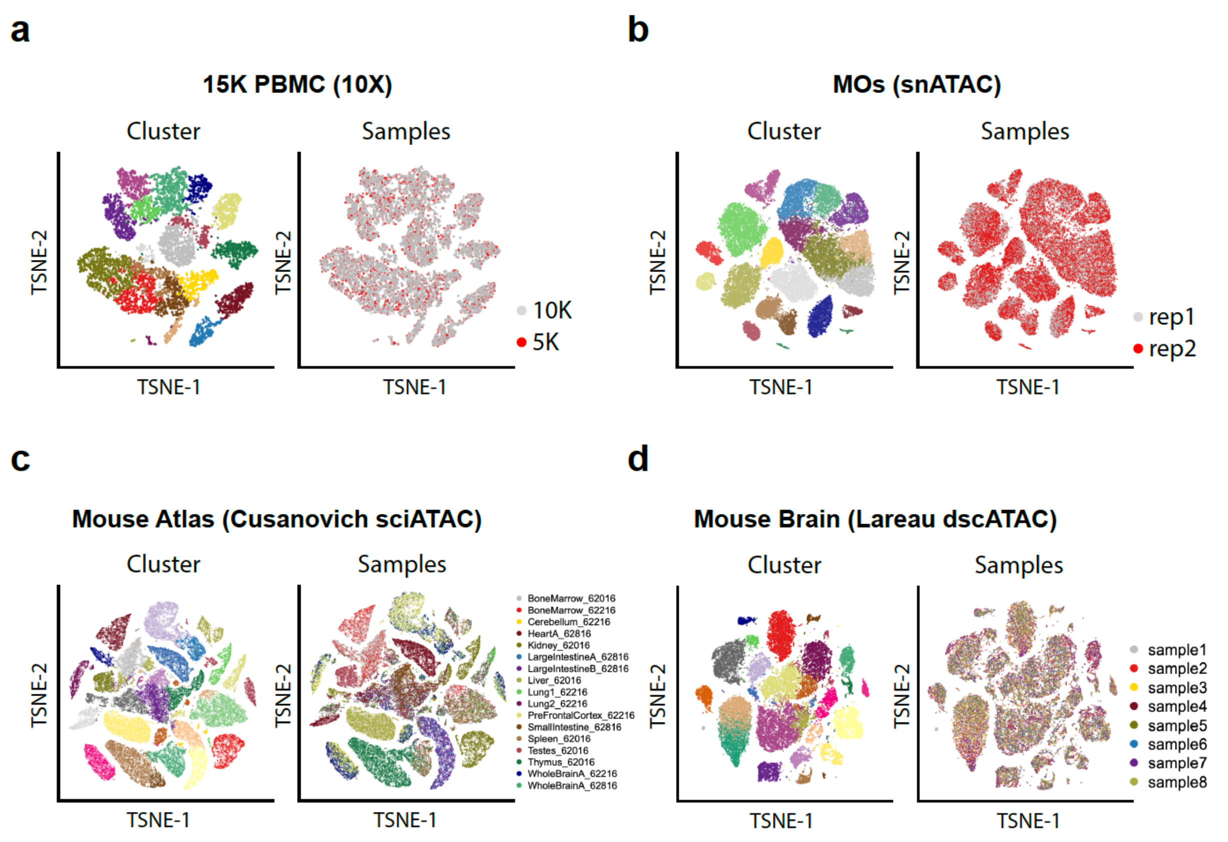

Supplementary Fig. 13. SnapATAC is robust to technical variation. Two-dimensional t-SNE visualization of four benchmarking datasets generated using SnapATAC. Cells are color by cluster label (left) and sample label (right). **a** 15k PBMC (10X) – a combination of two datasets (PBMC 5k and 10k) publicly available from 10X genomics. **b** MOs (snATAC) – an in-house dataset that contains two biological replicates from secondary motor cortex in the adult mouse brain generated using single nucleus ATAC-seq. **c** Mouse Atlas (Cusanovich 2018)[3] – a published dataset that contains over 80K cells from 13 different mouse tissues generated using multiplexing single cell ATAC-seq. **d** Mouse Brain (Lareau dscATAC)[12] – a published dataset that contains 46,652 cells from 8 samples in the adult mouse brain generated using BioRad droplet-based single cell ATAC-seq. Source of the datasets used for generating this plot is listed in Supplementary Table 1.

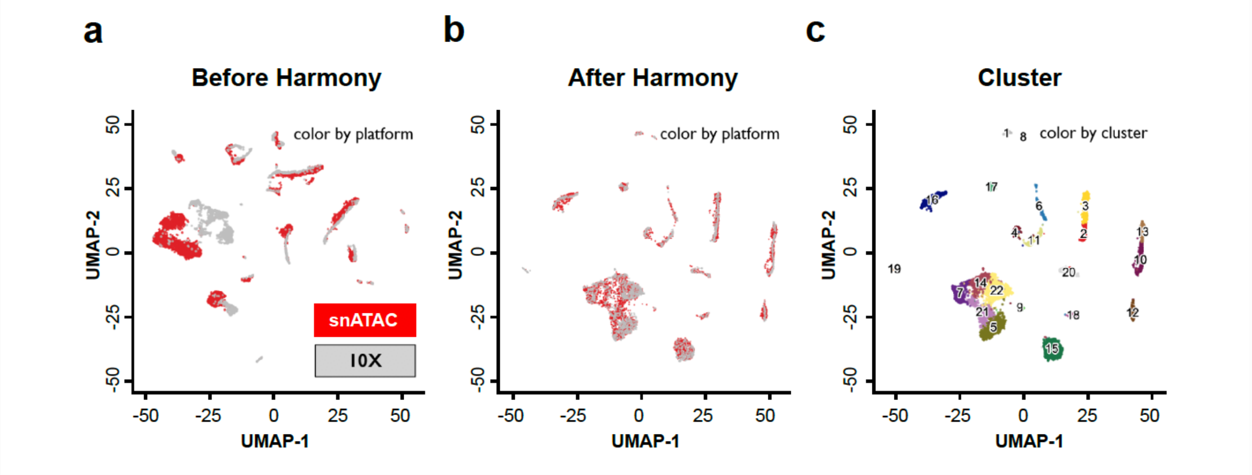

Supplementary Fig. 14. SnapATAC eliminates batch effect using Harmony[13]. The joint UMAP visualization of two datasets of mouse brain generated using combinatorial indexing single nucleus ATAC-seq (MOs-M1 snATAC) and droplet-based platform (Mouse Brain 10X) before (**a**) and after (**b**) performing batch effect correction using Harmony. Data source is listed in Supplementary Table 1.

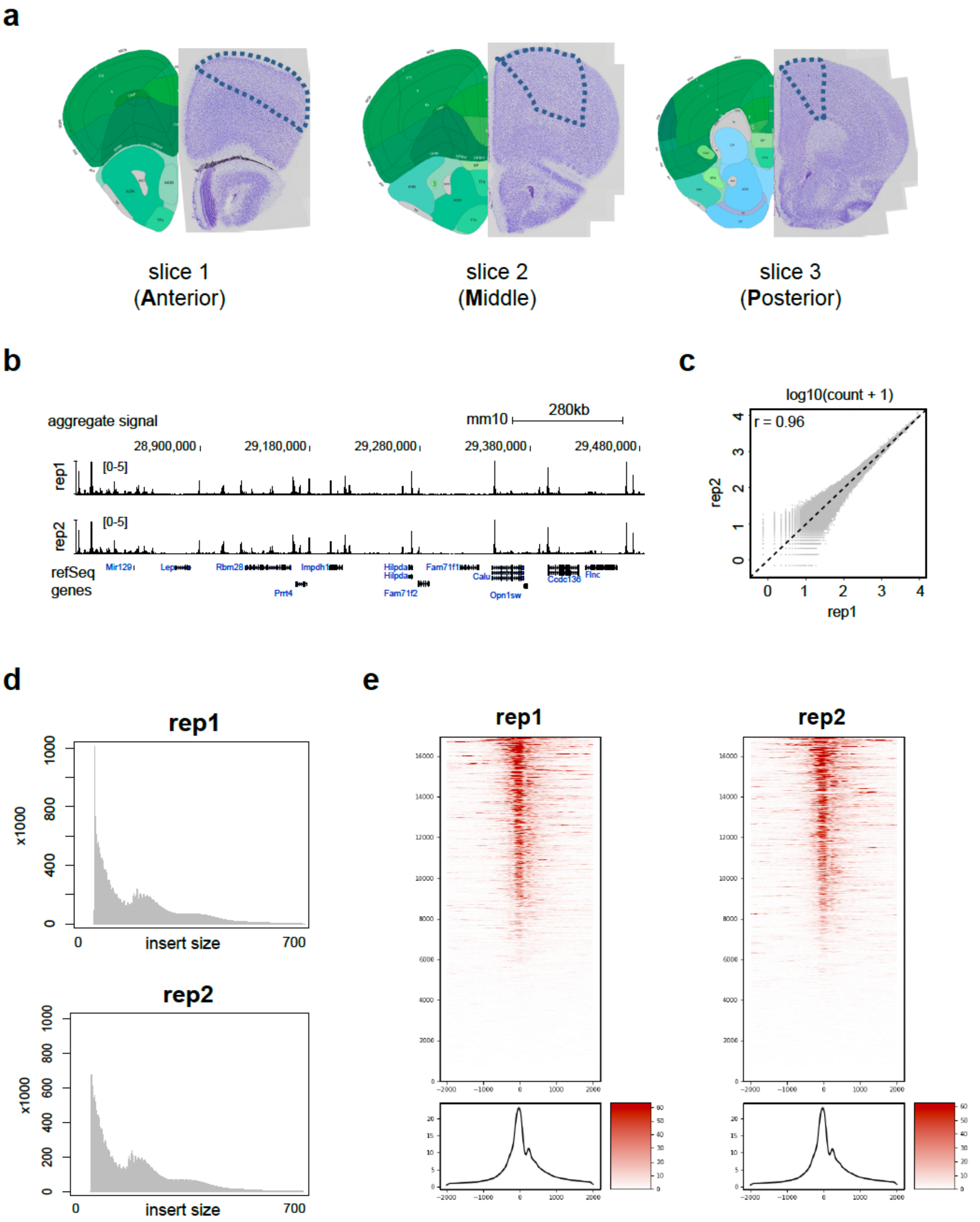
a
slice 1
(Anterior)
slice 2
(Middle)
slice 3
(Posterior)
b
aggregate signal
mm10
280kb
28,900,000
29,180,000
29,280,000
29,380,000
29,480,000
rep1
[0-5]
rep2
[0-5]
refSeq genes
Mir129
Lep
Rbm28
Impdh1
Prrt4
Hilpda
Fam71f1
Calu
Fam71f2
Opn1sw
Ccdc136
Flnc
c
log10(count + 1)
r = 0.96
rep2
rep1
d
rep1
x1000
insert size
0
700
rep2
x1000
insert size
0
700
e
rep1
rep2

Supplementary Fig. 15. Single nucleus ATAC-seq datasets are reproducible between biological replicates. a Illustration of dissection. Posterior view of three 0.6 mm coronal slices from which the secondary motor cortex (MOs) was dissected. The right side on each image depicts the corresponding view from the Allen Brain Atlas. The left side correspond to the Nissl staining of the posterior side of each slice. The MOs region was manually dissected according to the dashed lines on each slice and following the MOs as depicted in plates 27, 33, and 39 of the Allen Brain Atlas (left side images in figure). Each slice contains two biological replicates named as A1, A2, M1, M2, P1 and P2 (A: Anterior; M: Middle; P: Posterior). In this study, A1, M1 and P1 is combined as replicate 1 and A2, M2 and P2 are combined as replicate 2. **b** Genome-browser view of aggregate signal for two biological replicates. **c** Pearson correlation of count per million (CPM) at peaks between two replicates. **d** Insert size distribution and **e** TSS enrichment score for two biological replicates.

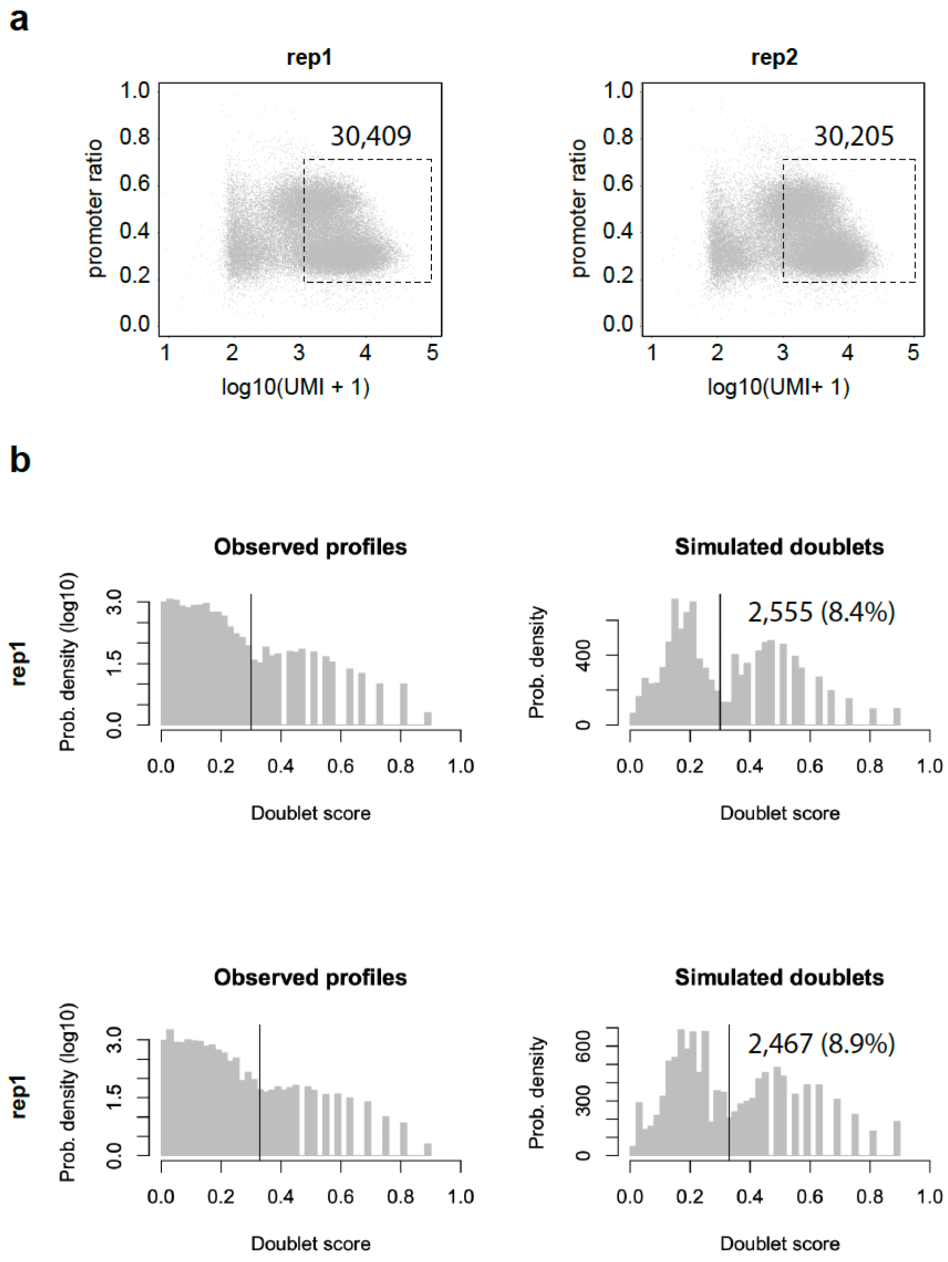

Supplementary Fig. 16. Barcode selection of MOs. a Cells of unique fragments within the range of 1,000-100,000 and fragments in promoter ratio within the range of 0.2-0.7 were selected. This resulted in 30,409 and 30,205 nuclei for two replicates. **b** With 5kb cell-by-bin matrix as input matrix, putative doublets were identified using Scrublets[14], which predicted 2,555 (8.4%) and 2,467 (8.9%) nuclei to be doublets for each replicate. The predicted doublet ratio is similar to the theoretical calculation of doublet ratio for multiplexing single cell ATAC-seq experiment[4,15].

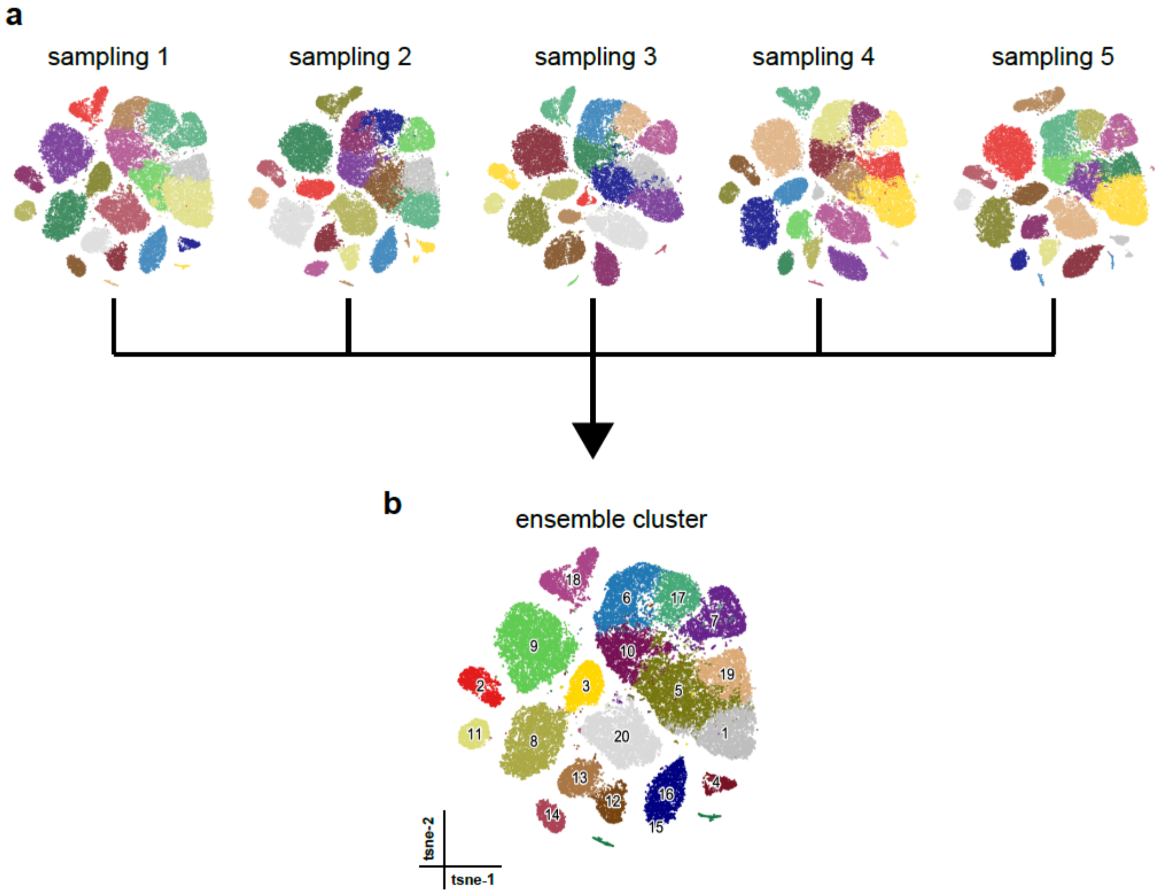

Supplementary Fig. 17. Consensus clustering of MOs. a Five clustering results were generated using SnapATAC with different set of landmarks (10,000). **b** These five clustering solutions were combined to create a consensus clustering which identified 20 clusters in MOs (Methods section).

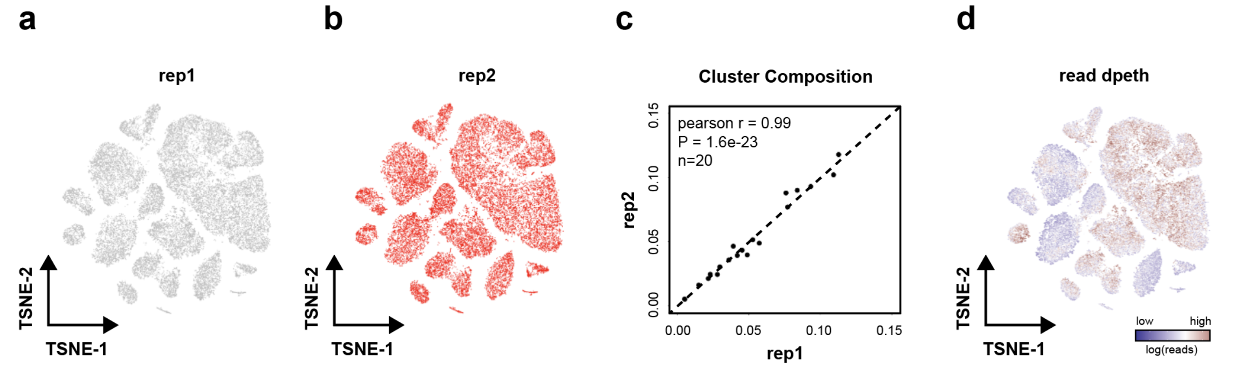

Supplementary Fig. 18. MOs clustering result is reproducible between biological replicates. (**a-b**) T-SNE visualization of cells from two biological replicates. **c** Percentage of 20 major clusters is highly reproducible between two biological replicates (Spearman's Rank Correlation Coefficient r=0.99 and P-value = 1.6e-23; n = 20 major clusters as shown in Fig. 5a). **d** T-SNE visualization of cells with color scaled by sequencing depth.

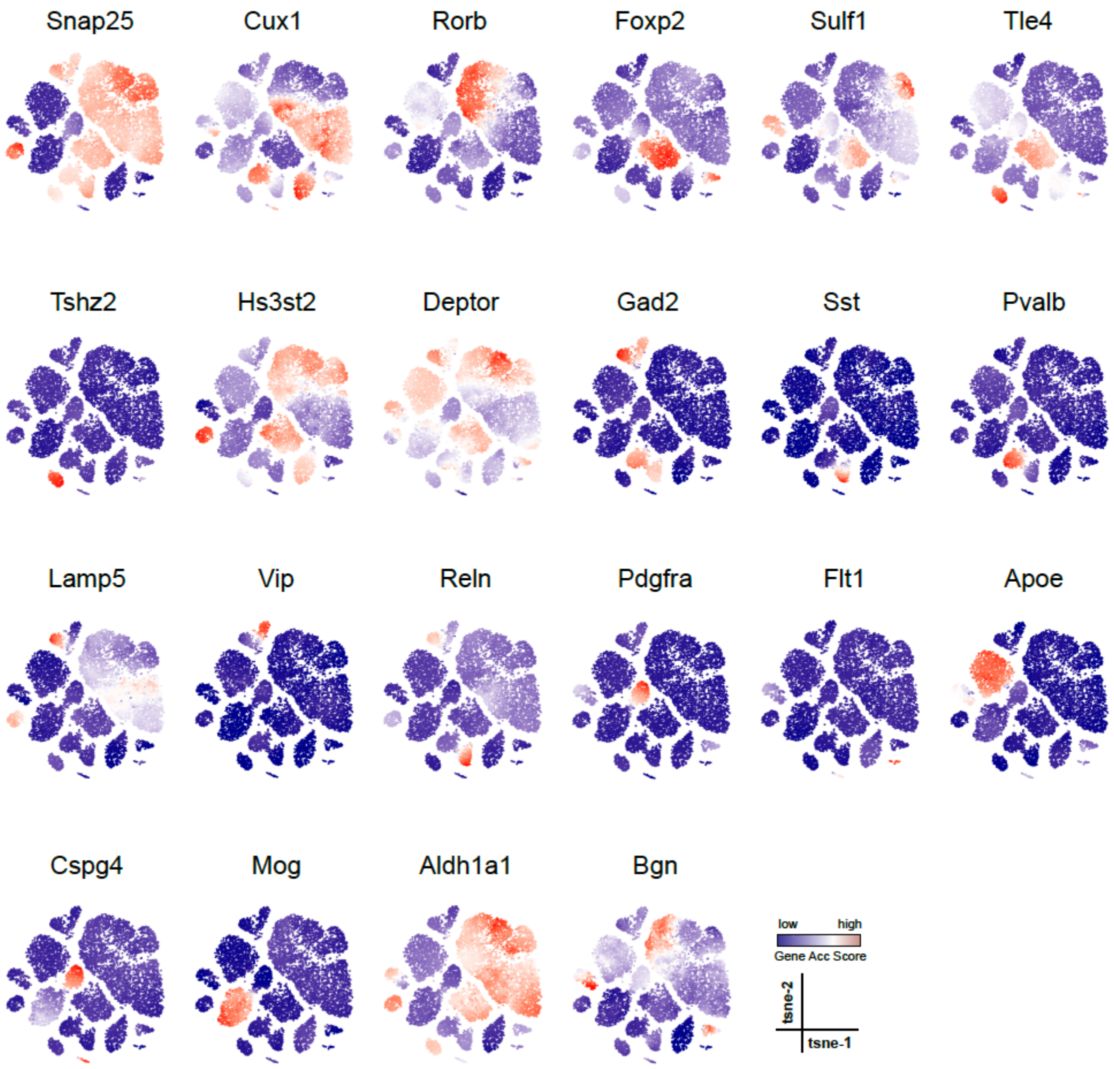

Supplementary Fig. 19. Gene accessibility score of canonical marker genes projected onto MOs t-SNE embedding to guide the cluster annotation. T-SNE is generated using SnapATAC for MOs; cell type specific marker genes was defined from previous single cell transcriptomic analysis in adult mouse brain[10]; gene accessibility score is calculated using SnapATAC (Methods section) and projected to the t-SNE embedding.

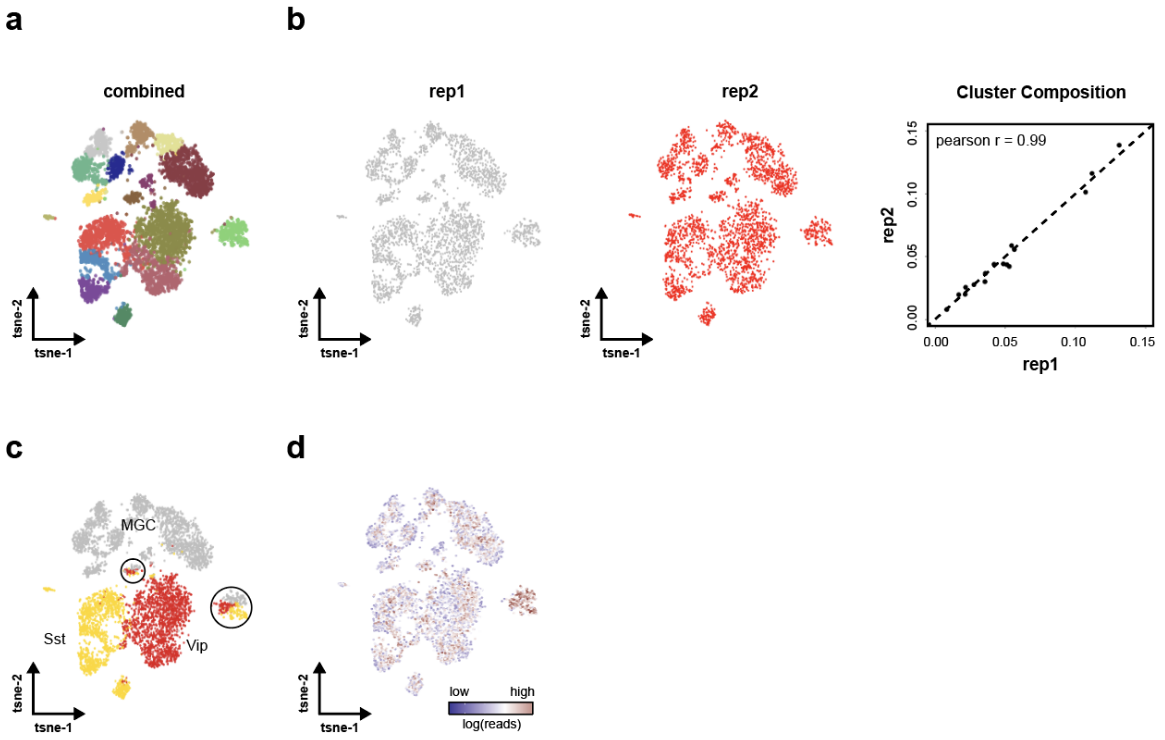

Supplementary Fig. 20. Iterative clustering identifies 17 GABAergic neuronal subtypes. a Iterative clustering of 5,940 GABAergic neurons identified 17 distinct cell clusters. **b** Cluster composition was highly reproducible between two biological replicates (n=17). **c** TSNE visualization of 5,940 GABAergic neurons colored by cell types identified in the initial clustering. Black circles mark clusters that are potential doublets, a mixture of multiple cell types. **d** TSNE plot of GABAergic neurons colored by sequencing depth.

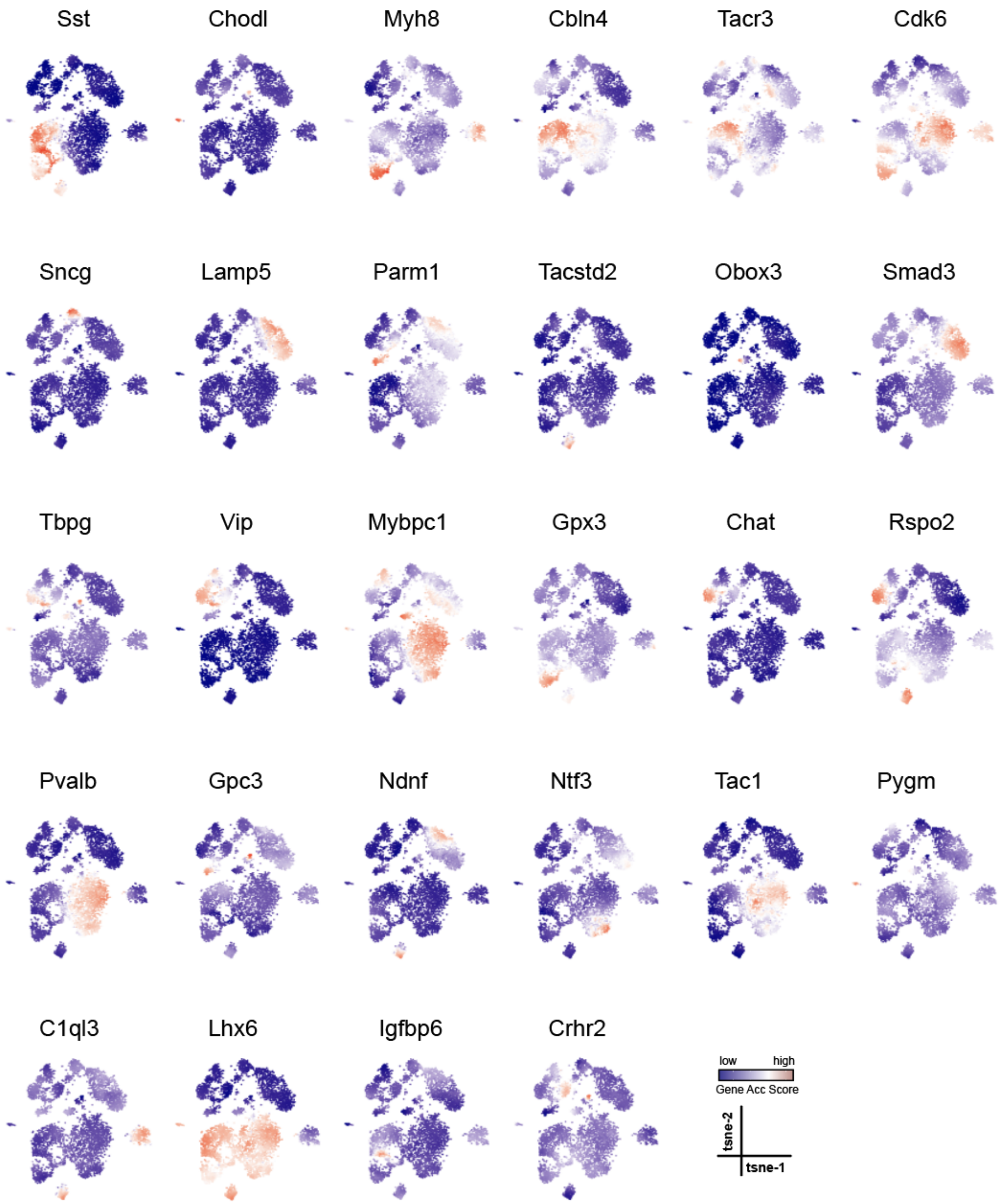
Sst
Chodl
Myh8
Cbln4
Tacr3
Cdk6
Sncg
Lamp5
Parm1
Tacstd2
Obox3
Smad3
Tbpg
Vip
Mybpc1
Gpx3
Chat
Rspo2
Pvalb
Gpc3
Ndnf
Ntf3
Tac1
Pygm
C1ql3
Lhx6
Igfbp6
Crhr2
low
high
Gene Acc Score
tsne-2
tsne-1

Supplementary Fig. 21. Gene accessibility score of marker genes projected onto t-SNE embedding from GABAergic neurons to guide the cluster annotation. Iterative clustering is performed against GABAergic neurons to identify subtypes. Twenty eight cell type specific marker genes were defined from previous single cell transcriptomic analysis in adult mouse brain[10]; gene accessibility score is calculated using SnapATAC (Methods section).

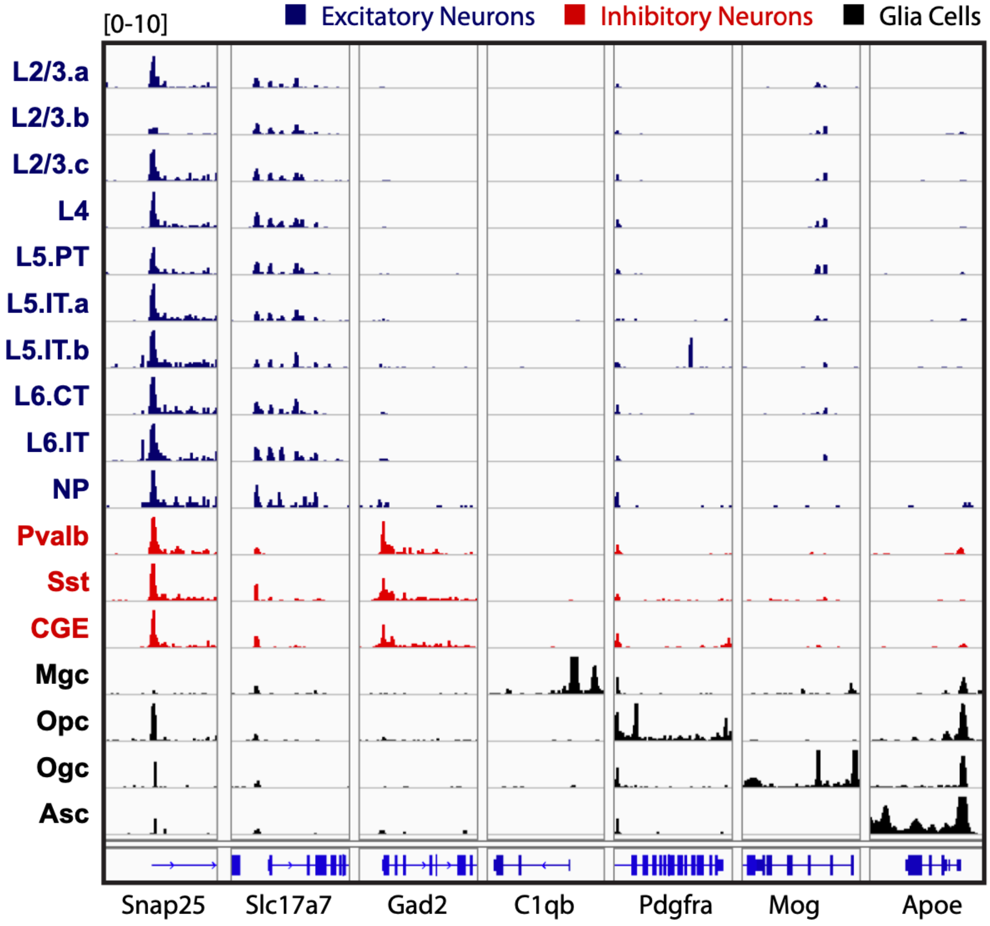

Supplementary Fig. 22. Genome browser view of aggregate signal for each of the major cell populations identified in the adult mouse brain.

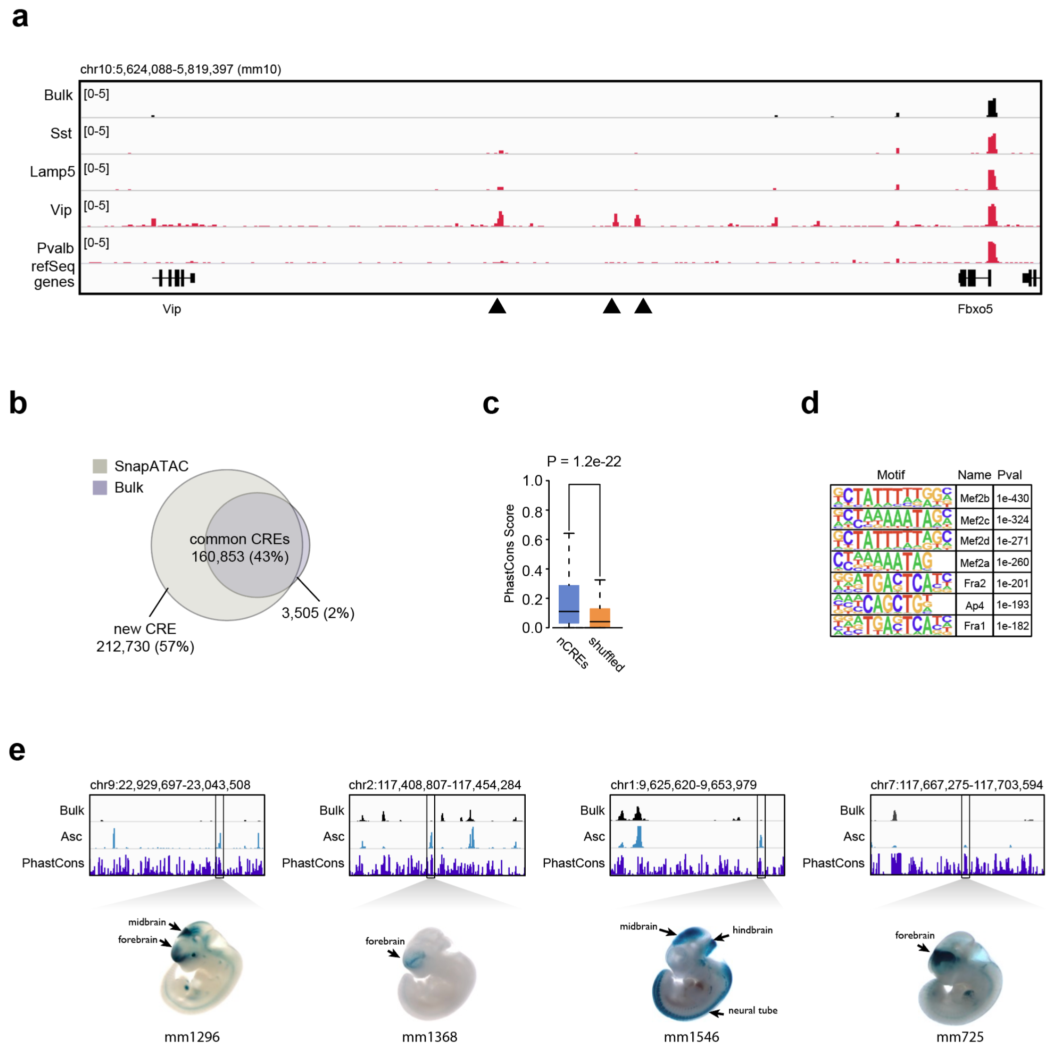

Supplementary Fig. 23. SnapATAC uncovers novel candidate *cis*-regulatory elements in rare cell types. a Genome browser view of 20Mb region flanking gene *Vip*. Dash lines highlight five regulatory elements specific to Vip subtypes that are under-represented in the conventional bulk ATAC-seq signal. **b** Over fifty percent of the regulatory elements identified from 20 major cell populations are not detected from bulk ATAC-seq data. **c** Sequence conservation comparison between the new elements and randomly chosen genomic regions (n=212,730; Wilcoxon Rank Sum p < 1e-22). Data are presented as 1.5 times interquartile range below 25% percentile, 25% percentile, 50% percentile, 75% percentile and 1.5 times interquartile range above 75% percentile. **d** Top seven motifs enriched in Pv-specific new elements (poisson p computed by HOMER[16]). **e** Examples of four new elements that were previously tested positive in transgenic mouse assays (image from VISTA database[17]) with enhancer ID below. Bulk: Bulk ATAC-seq; Asc: aggregated signal from astrocyte population (ASC) in the adult mouse brain as shown in Fig. 5.

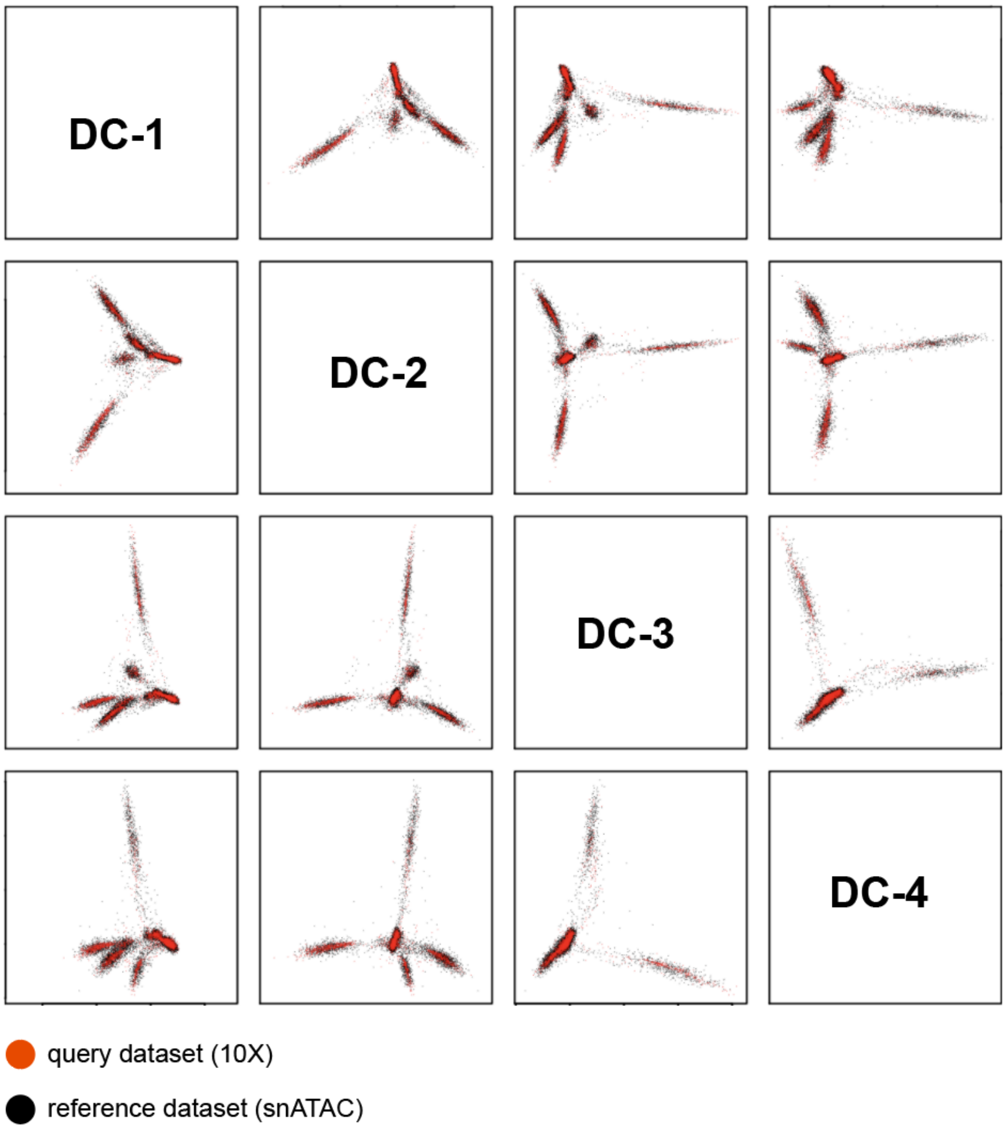

Supplementary Fig. 24. Joint embedding for query (Mouse Brain 10X) and reference dataset (MOs snATAC). The query dataset (10X) is projected onto the low dimension embedding space precomputed for the reference dataset (snATAC). Batch effect is corrected using Harmony. Pairwise plot of the first four dimentions in which cells are colored by dataset - red for query cells (Mouse Brain 10X) and black for reference cells (MOs snATAC). Data source as listed in Supplementary Table 1.

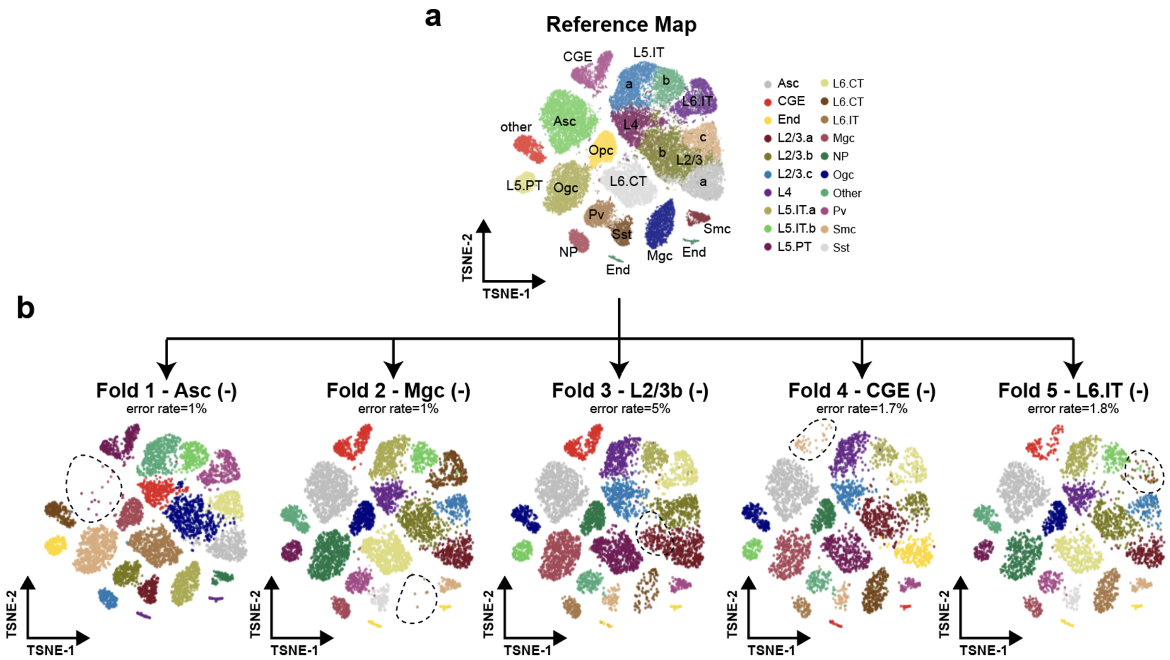

Supplementary Fig. 25. SnapATAC is robust for supervised annotation of datasets containing cell types missing in the reference atlas. a Two-dimensional t-SNE visualization of the reference dataset MOs (snATAC). **b** A five-fold cross validation is performed to this reference dataset. For each fold, we introduce perturbation to the 80% training dataset by randomly dropping one cell type (Asc, Mgc, L2/3b, CGE and L6.IT). We then predict on the 20% test dataset using the model learned from the perturbed training dataset. The prediction accuracy for each fold is shown in **b** and cell type removed from the training dataset are highlighted by the dash-line circles.

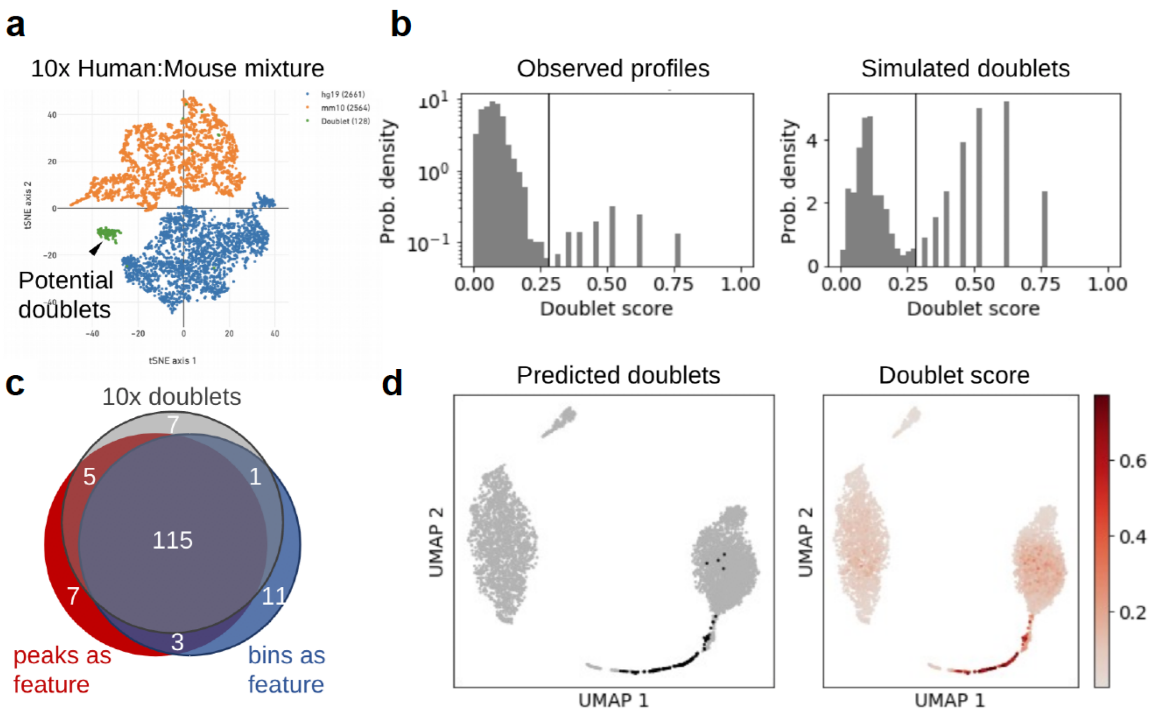

Supplementary Fig. 26. Doublets detection using Scrublet[14]. a T-SNE representation of a dataset (hgmm_1k 10X) that contained 1,000 human (GM12878) and mouse (A20) cells. Cells are colored by species determined based on the alignment ratio between human and mouse genome. Orange: A20; blue: GM12878; green: putative doublets. **b** Distribution of doublet score for putative doublets and simulated doublets estimated using Scrublet[14]. **c** Doublets are predicted using cell-by-peak and cell-by-bin matrix separately. Venn diagram shows the overlap between Scrublet-predicted doublets using peak or bin matrix and doublets identified based on alignment ratio. **d** Doublets scores projected onto the UMAP embedding.

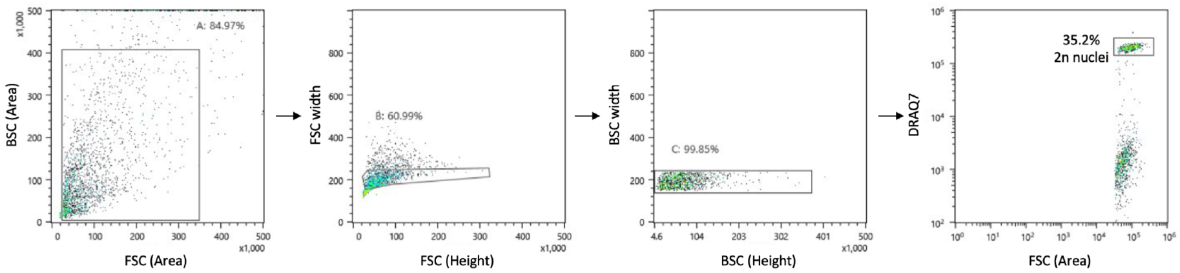

Supplementary Fig. 27. Nuclei sorting strategy. Gating strategy for nuclei sorting in snATAC-seq after tagmentatioon.

**Abbreviation**	**Data Source**	**URL**
5K PBMC (10X)	10X genomics	https://support.10xgenomics.com/single-cell-atac/datasets/1.2.0/atac_pbmc_5k_nextgem
15K PBMC (10X)	10X genomics	https://support.10xgenomics.com/single-cell-atac/datasets/1.2.0/atac_pbmc_10k_nextgem
10K PBMC (10X scRNA)	10X genomics	http://cf.10xgenomics.com/samples/cell-exp/3.0.0/pbmc_10k_v3/pbmc_10k_v3_filtered_feature_bc_matrix.h5
Embryonic Mouse Brain (10X)	10X genomics	https://support.10xgenomics.com/single-cell-atac/datasets/1.2.0/atac_v1_E18_brain_fresh_5k
Schep 2017 (C1)	Schep 2018[7]	https://www.ncbi.nlm.nih.gov/geo/query/acc.cgi?acc=GSE99172
Mos-A1 (snATAC)	this study	https://www.ncbi.nlm.nih.gov/geo/query/acc.cgi?acc=GSE126724
Mos-A2 (snATAC)	this study	https://www.ncbi.nlm.nih.gov/geo/query/acc.cgi?acc=GSE126724
Mos-M1 (snATAC)	this study	https://www.ncbi.nlm.nih.gov/geo/query/acc.cgi?acc=GSE126724
Mos-M2 (snATAC)	this study	https://www.ncbi.nlm.nih.gov/geo/query/acc.cgi?acc=GSE126724
Mos-P1 (snATAC)	this study	https://www.ncbi.nlm.nih.gov/geo/query/acc.cgi?acc=GSE126724
Mos-P2 (snATAC)	this study	https://www.ncbi.nlm.nih.gov/geo/query/acc.cgi?acc=GSE126724
Mos (snATAC)	this study	A combination of Mos-A1, Mos-A2, Mos-M1, Mos-M2, Mos-P1, Mos-P2
Mouse Brain (10X)	10X genomics	https://support.10xgenomics.com/single-cell-atac/datasets/1.1.0/atac_v1_adult_brain_fresh_5k
Mouse Atlas (Cusanovich sciATAC)	Cusanovich Cell 2018	https://www.ncbi.nlm.nih.gov/geo/query/acc.cgi?acc=GSE111586
Mouse Brain (Lareau BioRad)	Lareau Nature Biotechnology 2019	https://www.ncbi.nlm.nih.gov/geo/query/acc.cgi?acc=GSE123581
Pastki cells (Cusanovich sciATAC)	Cusanovich Science 2015	https://www.ncbi.nlm.nih.gov/geo/query/acc.cgi?acc=GSE67446
Human mouse mixture 1k (10X)	10X genomics	https://support.10xgenomics.com/single-cell-

		atac/datasets/1.2.0/atac_hgmm_1k_nextgem
Human Bonne Marrow (Lareau 2019)	Lareau et al. Nature Biotechnology (2019)	https://www.ncbi.nlm.nih.gov/geo/query/acc.cgi?acc=GSE123581
BCC TME (Satpathy 2019)	Satpathy et. Al. Nature Biotechnology (2019)	https://www.ncbi.nlm.nih.gov/geo/query/acc.cgi?acc=GSE129785

Supplementary Table 1. Source of single cell datasets used in this study.

ENCODE INDEX	Cell Type Name
ENCFF199ZJX	erythroblast
ENCFF655PLY	granulocyte monocyte progenitor cell
ENCFF250YAL	hematopoietic stem cell
ENCFF647NFF	megakaryocyte
ENCFF471OUM	megakaryocyte progenitor cell
ENCFF901IMU	megakaryocyte-erythroid progenitor cell
ENCFF437VEJ	monocyte
ENCFF606PRC	G1E
ENCFF366SLO	neutrophil
ENCFF053CGD	regulatory T cell

Supplementary Table 2. Source of ENCODE bulk ATAC-seq used for generating the simulated single cell ATAC-seq datasets.

name	Total Fragments	Uniquely Mapped	Properly Paired	Unique Fragments	ChrM fragments	Library Efficiency
**A1**	68,822,246	64,182,717 (93%)	64,100,432 (99%)	49,557,725 (77%)	524,164 (1.0%)	72%
**A2**	55,030,523	51,348,532 (93%)	51,291,913 (99%)	40,270,010 (78%)	468,994 (1.1%)	73%
**M1**	73,706,903	68,778,680 (93%)	68,628,077 (99%)	49,250,682 (71%)	416,721 (0.8%)	66%
**M2**	88,766,084	83319732 (94%)	83,318,989 (99%)	60,990,045 (74%)	394,825 (0.6%)	68%
**P1**	85,104,213	79,249,561 (93%)	79,010,287 (99%)	58,341,065 (74%)	477,236 (0.8%)	68%
**P2**	79,673,741	74,065,766 (93%)	73,869,579 (99%)	49,283,602 (66%)	565,863 (1.1%)	61%

Supplementary Table 3. Quality control metric for single nucleus ATAC-seq libraries. A1= anterior rep1; A1= anterior rep1; M1= middle rep1; M2 = middle rep1; P1 = posterior rep1; P2 = posterior rep 2.

ID	Index Sequence
T5_9	TTCGGAAG
T5_10	AGGCTGGT
T5_11	GGAAGACT
T5_12	GAACGCAT
T5_13	TGCTGGTA
T5_14	CATTCAGT
T5_15	ACAAGGAT
T5_16	TGTCAGCT
T7_12	CTATTAGG
T7_13	CATGTCAG
T7_14	CTCATACA
T7_15	AGATCTTC
T7_16	AGAGCAGT
T7_17	AGACGGAG
T7_18	TGTGCAAC
T7_19	ACATTGGC
T7_20	TGTTACCA
T7_21	GCTCTAAG
T7_22	TATCGGTT
T7_23	CAGTTGCA

Supplementary Table 4. Index sequences used in the single nucleus ATAC-seq.

Name	Num
Total number of molecules sequenced	75,925,182
Read count from sequencer	151,850,364
Read count successfully aligned	150,315,670
Read count after filtering for mapping quality	136,555,794
Read count after removing duplicate reads	130,680,670
Read count after removing mitochondrial reads	129,415,200
Final number of fragments	64,707,600

Supplementary Table 5. Quality control metrics for bulk ATAC-seq library.

References

1. Li, H. & Durbin, R. Fast and accurate short read alignment with Burrows-Wheeler transform. *Bioinformatics* **25**, 1754–1760 (2009).
2. Bravo González-Blas, C. *et al.* cisTopic: cis-regulatory topic modeling on single-cell ATAC-seq data. *Nat. Methods* **16**, 397–400 (2019).
3. Cusanovich, D. A. *et al.* A Single-Cell Atlas of In Vivo Mammalian Chromatin Accessibility. *Cell* **174**, 1309-1324.e18 (2018).
4. Cusanovich, D. A. *et al.* Multiplex single-cell profiling of chromatin accessibility by combinatorial cellular indexing. *Science* **348**, 910–914 (2015).
5. Kumar, S., Mohri, M. & Talwalkar, A. Ensemble Nystrom Method. in *Proceedings of the 22nd International Conference on Neural Information Processing Systems* 1060–1068 (2009).
6. Stuart, T. *et al.* Comprehensive Integration of Single-Cell Data. *Cell* **177**, 1888-1902.e21 (2019).
7. Schep, A. N., Wu, B., Buenrostro, J. D. & Greenleaf, W. J. chromVAR: inferring transcription-factor-associated accessibility from single-cell epigenomic data. *Nat. Methods* **14**, 975–978 (2017).
8. Pliner, H. A. *et al.* Cicero Predicts cis-Regulatory DNA Interactions from Single-Cell Chromatin Accessibility Data. *Mol. Cell* **71**, 858-871.e8 (2018).
9. Buenrostro, J. D. *et al.* Single-cell chromatin accessibility reveals principles of regulatory variation. *Nature* **523**, 486–490 (2015).
10. Tasic, B. *et al.* Adult mouse cortical cell taxonomy revealed by single cell transcriptomics. *Nat. Neurosci.* **19**, 335–346 (2016).
11. Rao, S. S. P. *et al.* A 3D Map of the Human Genome at Kilobase Resolution Reveals Principles of Chromatin Looping. *Cell* **159**, 1665–1680 (2014).
12. Lareau, C. A. *et al.* Droplet-based combinatorial indexing for massive-scale single-cell chromatin accessibility. *Nat. Biotechnol.* **37**, 916–924 (2019).
13. Korsunsky, I. *et al.* Fast, sensitive and accurate integration of single-cell data with Harmony. *Nat. Methods* **16**, 1289–1296 (2019).
14. Wolock, S. L., Lopez, R. & Klein, A. M. Scrublet: Computational Identification of Cell Doublets in Single-Cell Transcriptomic Data. *Cell Syst.* **8**, 281-291.e9 (2019).
15. Preissl, S. *et al.* Single-nucleus analysis of accessible chromatin in developing mouse forebrain reveals cell-type-specific transcriptional regulation. *Nat. Neurosci.* **21**, 432 (2018).
16. Heinz, S. *et al.* Simple Combinations of Lineage-Determining Transcription Factors Prime cis-Regulatory Elements Required for Macrophage and B Cell Identities. *Mol. Cell* **38**, 576–589 (2010).
17. Visel, A., Minovitsky, S., Dubchak, I. & Pennacchio, L. A. VISTA Enhancer Browser--a database of tissue-specific human enhancers. *Nucleic Acids Res.* **35**, D88–D92 (2007).
