# Supplementary material for: Comprehensive analysis of single cell ATAC-seq data with SnapATAC
Source: Nat Commun. 2021 Feb 26;12:1337. doi: 10.1038/s41467-021-21583-9 (PMC7910485; doi:10.1038/s41467-021-21583-9)
Supplement: Supplementary file 3 — Reporting Summary [file 41467_2021_21583_MOESM3_ESM.pdf]

nature research

Corresponding author(s): Bing Ren

Last updated by author(s): 01/04/2021

Reporting Summary

Nature Research wishes to improve the reproducibility of the work that we publish. This form provides structure for consistency and transparency in reporting. For further information on Nature Research policies, see Authors & Referees and the Editorial Policy Checklist .

Statistics

For all statistical analyses, confirm that the following items are present in the figure legend, table legend, main text, or Methods section.

n/a	Confirmed	
☐	☒	The exact sample size (*n*) for each experimental group/condition, given as a discrete number and unit of measurement
☐	☒	A statement on whether measurements were taken from distinct samples or whether the same sample was measured repeatedly
☐	☒	The statistical test(s) used AND whether they are one- or two-sided<br>*Only common tests should be described solely by name; describe more complex techniques in the Methods section.*
☐	☒	A description of all covariates tested
☐	☒	A description of any assumptions or corrections, such as tests of normality and adjustment for multiple comparisons
☐	☒	A full description of the statistical parameters including central tendency (e.g. means) or other basic estimates (e.g. regression coefficient) AND variation (e.g. standard deviation) or associated estimates of uncertainty (e.g. confidence intervals)
☐	☒	For null hypothesis testing, the test statistic (e.g. *F*, *t*, *r*) with confidence intervals, effect sizes, degrees of freedom and *P* value noted<br>*Give P values as exact values whenever suitable.*
☒	☐	For Bayesian analysis, information on the choice of priors and Markov chain Monte Carlo settings
☐	☒	For hierarchical and complex designs, identification of the appropriate level for tests and full reporting of outcomes
☐	☒	Estimates of effect sizes (e.g. Cohen's *d*, Pearson's *r*), indicating how they were calculated

Our web collection on statistics for biologists contains articles on many of the points above.

Software and code

Policy information about availability of computer code

Data collection: No software was used for data collection

Data analysis:
custom code used in this study is made available at https://github.com/r3fang/SnapATAC
The software and versions used in this study are:
BWA v0.7.13-r1126
bedtools v2.27.1
edgeR v3.18.1
HOMER v4.9.1
LIMMA v3.32.3
samtools v1.9
samtools v1.2
numpy v1.14.3
scipy v1.1.0
rhdf5 v2.20.0
python 2.7
R (3.4.0)
h5py v2.8.0
pybedtools v0.7.10
python-louvain v0.13
pysam v0.15.2
Seurat V3
chromVAR (1.12.0)
cisTopic (v3)

MACS2 (v2.1.1.20160309)
MACS2 (version 2.1.2)

For manuscripts utilizing custom algorithms or software that are central to the research but not yet described in published literature, software must be made available to editors/reviewers. We strongly encourage code deposition in a community repository (e.g. GitHub). See the Nature Research guidelines for submitting code & software for further information.

Data

Policy information about availability of data

All manuscripts must include a data availability statement. This statement should provide the following information, where applicable:
- Accession codes, unique identifiers, or web links for publicly available datasets
- A list of figures that have associated raw data
- A description of any restrictions on data availability

Raw and processed data generated during this study have been deposited to NCBI Gene Expression Omnibus with the accession number GSE126724. Previously published data used for this study are within Supplementary Table 1 as well as listed below:

VISTA Enhancer Browser - https://enhancer.lbl.gov/
ENCODE Database - https://www.encodeproject.org/
5K PBMC (10X) - https://support.10xgenomics.com/single-cell-atac/datasets/1.2.0/atac_pbmc_5k_nextgem
15K PBMC (10X) - https://support.10xgenomics.com/single-cell-atac/datasets/1.2.0/atac_pbmc_10k_nextgem
10K PBMC (10X scRNA) - http://cf.10xgenomics.com/samples/cell-exp/3.0.0/pbmc_10k_v3/pbmc_10k_v3_filtered_feature_bc_matrix.h5
Embryonic Mouse Brain (10X) - https://support.10xgenomics.com/single-cell-atac/datasets/1.2.0/atac_v1_E18_brain_fresh_5k
Human mouse mixture 1k (10X) - https://support.10xgenomics.com/single-cell-atac/datasets/1.2.0/atac_hgmm_1k_nextgem
Schep 2017 (C1) - https://www.ncbi.nlm.nih.gov/geo/query/acc.cgi?acc=GSE99172
Mouse Brain (10X) - https://support.10xgenomics.com/single-cell-atac/datasets/1.1.0/atac_v1_adult_brain_fresh_5k
Mouse Atlas (Cusanovich sciATAC) - https://www.ncbi.nlm.nih.gov/geo/query/acc.cgi?acc=GSE111586
Mouse Brain (Lareau BioRad) - https://www.ncbi.nlm.nih.gov/geo/query/acc.cgi?acc=GSE123581
Pastki cells (Cusanovich sciATAC) - https://www.ncbi.nlm.nih.gov/geo/query/acc.cgi?acc=GSE67446
Human Bonne Marrow - https://www.ncbi.nlm.nih.gov/geo/query/acc.cgi?acc=GSE123581
BCC TME (Satpathy 2019) - https://www.ncbi.nlm.nih.gov/geo/query/acc.cgi?acc=GSE129785

Field-specific reporting

Please select the one below that is the best fit for your research. If you are not sure, read the appropriate sections before making your selection.

☒ Life sciences ☐ Behavioural & social sciences ☐ Ecological, evolutionary & environmental sciences

For a reference copy of the document with all sections, see nature.com/documents/nr-reporting-summary-flat.pdf

Life sciences study design

All studies must disclose on these points even when the disclosure is negative.

Sample size	snATAC-seq libraries were prepared in two independent experiments to ensure the robustness of the snATAC-seq procedure and reproducibility of the data quality.
Data exclusions	No data was excluded.
Replication	All two batches of snATAC-seq experiments were performed independently
Randomization	The samples were not randomly allocated into experimental groups because every sample consisted of cells of the same cell types taken into experiment from the same biological and growth conditions.
Blinding	The investigators were not blinded during data collection and analysis because the snATAC-seq experiments were performed on the untreated mice grown in the same biological conditions.

Reporting for specific materials, systems and methods

We require information from authors about some types of materials, experimental systems and methods used in many studies. Here, indicate whether each material, system or method listed is relevant to your study. If you are not sure if a list item applies to your research, read the appropriate section before selecting a response.

Materials & experimental systems

n/a	Involved in the study
☒	☐ Antibodies
☒	☐ Eukaryotic cell lines
☒	☐ Palaeontology
☐	☒ Animals and other organisms
☒	☐ Human research participants
☒	☐ Clinical data

Methods

n/a	Involved in the study
☒	☐ ChIP-seq
☐	☒ Flow cytometry
☒	☐ MRI-based neuroimaging

Animals and other organisms

Policy information about studies involving animals; ARRIVE guidelines recommended for reporting animal research

Laboratory animals	Male C57BL/6J mice were purchased from Jackson laboratories at 8 weeks of age and maintained in the Salk animal barrier facility on 12-hr dark-light cycles with controlled temperature (20-22 °C range) and humidity (30-70% range), and food ad libitum for one week before dissection.
Wild animals	No wild animals were used in this study
Field-collected samples	No field-collected samples were used in this study
Ethics oversight	Animal protocols were approved by the Salk Institute Institutional Animal Care and Use Committee.

Note that full information on the approval of the study protocol must also be provided in the manuscript.

Flow Cytometry

Plots

Confirm that:

☐ The axis labels state the marker and fluorochrome used (e.g. CD4-FITC).

☐ The axis scales are clearly visible. Include numbers along axes only for bottom left plot of group (a 'group' is an analysis of identical markers).

☐ All plots are contour plots with outliers or pseudocolor plots.

☐ A numerical value for number of cells or percentage (with statistics) is provided.

Methodology

Sample preparation	NeuN: Nuclei were incubated with 1:1000 dilution1:1000 dilution of AlexaFluor488 conjugated anti-NeuN antibody (MAB377X, Millipore) at 4°C for 1 hour.<br>snATAC-seq: Nuclei were stained 3 μM Draq7 (#7406, Cell Signaling)
Instrument	NeuN: Influx (BD), 85 μm nozzle at 22.5 PSI sheath pressure, snATAC: Sony SH800
Software	NeuN: BD FACS snATAC: Sony SH800S software
Cell population abundance	NeuN; 63% NeuN positive and 36% NeuN negative nuclei based on fluorescence intensity form AlexaFluor488
Gating strategy	NeuN: Potential nuclei were first identified based on FSC/SSC gates. Next doublets were removed based on SSC and FSC signal width. Single nuclei were analysed<br>snATAC: Potential nuclei were first identified based on FSC/BSC gates. Next doublets were removed based on BSC and FSC signal width. DRAQ7 positive single nuclei with 2n count were sorted.

☒ Tick this box to confirm that a figure exemplifying the gating strategy is provided in the Supplementary Information.
